# Benchmarking the Physical Performance Qualities in Women’s Football: A Systematic Review and Meta-analysis Across the Performance Scale

**DOI:** 10.1007/s40279-025-02251-0

**Published:** 2025-09-01

**Authors:** Heidi R. Compton, Ric Lovell, Dawn Scott, Jo Clubb, Tzlil Shushan

**Affiliations:** 1 School of Biomedical Sciences and Pharmacy, University of Newcastle, Callaghan, Australia; 2 Applied Sport Science and Exercise Testing Laboratory, University of Newcastle, Ourimbah, Australia; 3 FIFA, Women’s Development Programme, Women’s Football Division, Zurich, Switzerland; 4 Faculty of Science, Medicine and Health, University of Wollongong, Wollongong, Australia; 5 Global Performance Insights Ltd, London, UK

## Abstract

**Background:**

There is a lack of data and its systematic organisation relating to the physical performance qualities of women’s football players across the performance scale (i.e. level of competition categorised into unique tiers).

**Objectives:**

(1) To establish meta-analytic estimates for select assessment protocols across seven physical qualities (cardiorespiratory fitness, sprint time, acceleration time, change of direction, lower limb power, lower limb strength, and maximal velocity), and (2) to investigate the moderating effect of participant tier; collectively providing normative benchmarking data.

**Methods:**

A systematic literature search of four databases (Pubmed, SportDiscuss, Scopus, and Web of Science) for studies published between 2003 and 2023 was conducted, with a secondary search for studies published until August 2024. Studies were considered for inclusion if they were published in English, the population was female football players with a minimum mean age of 16 years and the studies reported descriptive data from selected testing protocols. All study designs were eligible, excluding acute interventions such as training or supplementation. Performance scale (i.e. participant tier) of the study participants was categorised into four distinct levels (Tier 2 to Tier 5) using a modified version of the Participant Classification Framework, with Tier 5 representing world-class athletes. Study risk of bias assessment was conducted using an adapted version of the Downs and Black tool. Means and standard deviations were analysed using mixed-effects, multilevel hierarchical models to obtain pooled estimates, 90% confidence intervals (CIs) and prediction intervals (PIs). Meta-regression of modifying effects for participant tier was conducted, and comparisons were expressed as standardised mean differences.

**Results:**

The final dataset included 1855 estimates from 982 groups across 288 studies. The moderating effect of participant tier was assessed where adequate data permitted. Yo-Yo Intermittent Recovery Test Level 1 (YYIRL1) demonstrated moderate-to-large improvements when progressing from Tier 2 to Tiers 4 and 5 (combined; [*b* = 170 to 354 m]); similar improvements were observed for velocity attained during the 30–15 Intermittent Fitness Test when directly comparing Tier 2 to Tiers 4 and 5 (*b* = 2.5 km·h^−1^). Sprint time decreased when progressing between tiers, with a moderate-to-large reduction for both 20 m (*b* =  − 0.17 to − 0.22 s) and 30 m (*b* =  − 0.32 to − 0.47 s) time when comparing Tier 2 with Tier 3 and Tiers 4 and 5, respectively. Moderate-to-large improvements in jump height were observed for squat jump (SJ; restricted arm movement) between participant tiers (*b* = 3.6 to 6.2 cm) and similarly for countermovement jump (CMJ) when comparing Tier 3 with Tiers 4 and 5 for restricted (*b* = 3.3 cm) and unrestricted arm movement (*b* = 8.8 cm).

**Conclusions:**

Normative benchmarks that are useful for athlete profiling and development, talent identification, and training program design have been established from a very large sample of studies and athletes. These findings highlight the role of sprinting ability, lower limb power and intermittent aerobic capacity in differentiating athletes across the performance scale. Field-based tests, particularly those assessing intermittent fitness, demonstrated the greatest difference in performance between each of the participant tiers, suggesting that these pragmatic tests are effective at capturing the physical performance of women’s football players. A limitation of this study is the variability introduced by the heterogeneity across studies in testing protocols, sample sizes and competition levels, which may have influenced the results.

**Registration:**

Prospective protocol registration can be found in Open Science Framework and is available through: https://doi.org/10.17605/OSF.IO/8W3JH.

**Graphical abstract:**

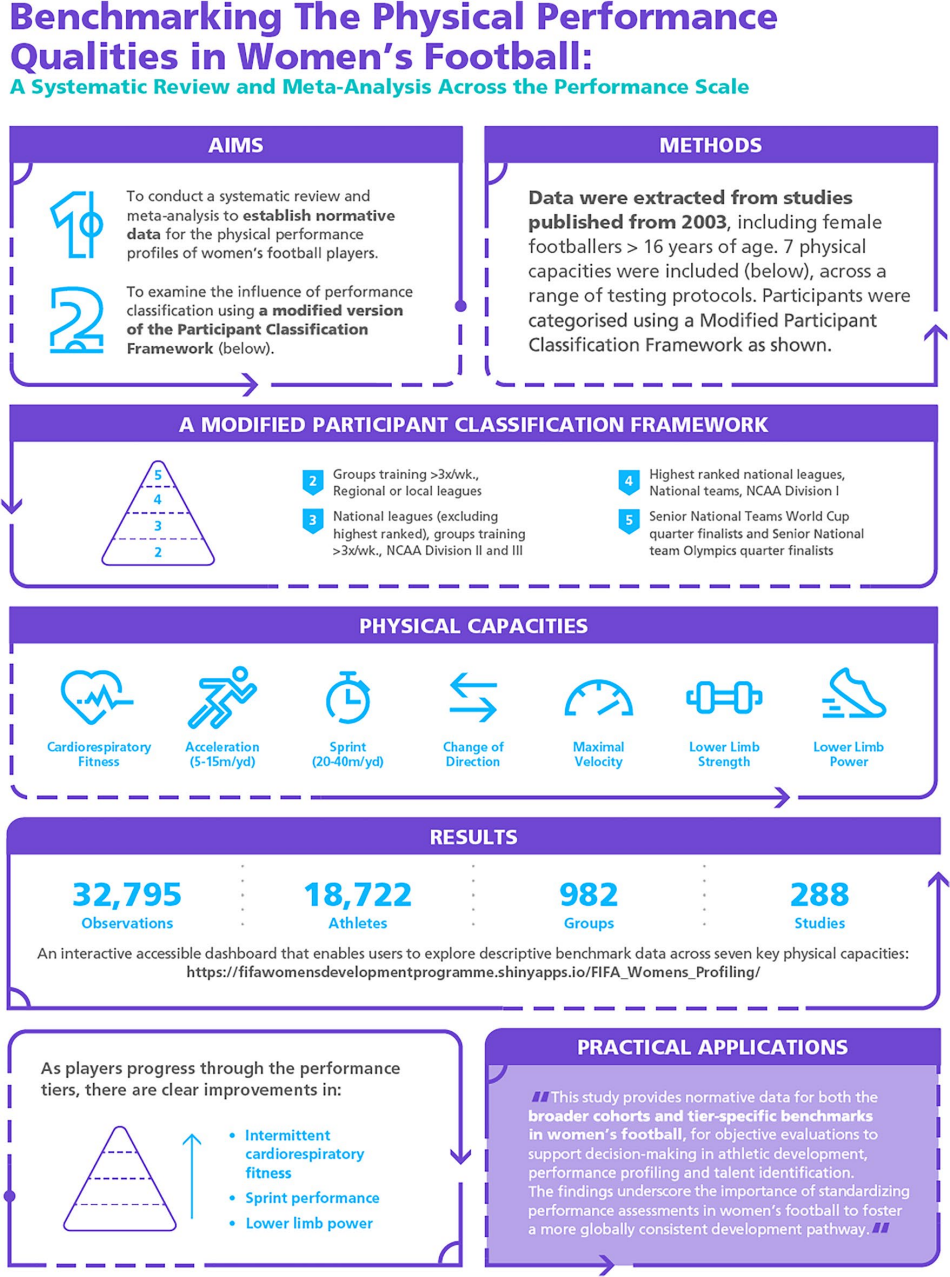

**Supplementary Information:**

The online version contains supplementary material available at 10.1007/s40279-025-02251-0.

## Key Points

**Table:** 

This systematic review meta-analysis establishes normative data for the physical performance profiles in women’s football, synthesising data from 18,722 athletes and 32,795 individual observations.
Intermittent aerobic capacity, sprint performance and lower limb power were greater in higher participant tiers (i.e. competition level), with the most pronounced differences observed between Tier 2 and Tiers 4 and 5.
Field-based assessments of intermittent aerobic capacity, including the YYIRL1 and the 30–15 Intermittent Fitness Test final velocity, effectively differentiated cardiorespiratory fitness across performance levels, with higher results observed in Tiers 4 and 5 compared with Tier 2.
The normative benchmarks generated from this analysis offer valuable insights for women’s football practitioners, allowing for more objective evaluations to support decision-making in the areas of athletic development, performance readiness and talent identification.
An interactive accessible dashboard has been created that enables users to explore descriptive data across seven key physical capacities, explore key findings, and access practical tools for benchmarking in women’s football that can be viewed (https://fifawomensdevelopmentprogramme.shinyapps.io/FIFA_Womens_Profiling/).

## Introduction

The global growth of women’s football has reached unprecedented heights, as demonstrated by the overwhelming success of the Fédération Internationale de Football Association (FIFA) Women’s World Cup Australia & New Zealand 2023™. This tournament not only broke records for ticket sales, broadcast viewership and digital media interactions [1–4] but also showcased the increasing physicality and intensity of women’s football matches at the highest level [5]. Further, the European Football Association (EUFA) EURO 2022 Championship marked a significant milestone in the progress of women’s football, where the final match had more attending fans than any other women’s European football championship in history for both men’s and women’s events [6]. However, with this rapid growth comes the responsibility to ensure that athlete development, welfare, training methods, medical provision and competitive standards evolve to match the increasing running demands of the game [8, 14, 15].

Elite-level football requires a diverse range of physical capacities to support both offensive and defensive actions [7, 8]. Aerobic and anaerobic endurance are essential for meeting the sport’s rigorous demands, where players are covering distances of between 9 km and 11.5 km per match [9], including substantial high-speed running (i.e. > 19 km h^−1^) [10]. Matches involve frequent high-intensity actions, requiring players to accelerate, decelerate and change direction rapidly [11]. Combined with the high volume of intense running [10], this demonstrates the need for repeated sprint ability and muscular endurance. Further, physical attributes, such as maximal velocity, lower-limb strength, and power, enhance explosive movements such as jumping, striking and tackling [12]. As professional women’s football leagues and international competitions become more prominent, it may be important to understand the typical physical qualities that define successful players, a central component of the ergonomic model [13]. This information about athletes’ physical qualities can be used for profiling purposes, guiding the design and evaluation of training programs, developing targeted injury prevention strategies and supporting informed decisions in team selection and tactical planning [13]. However, there remains a notable gap in literature regarding the physical profiling of female football players, particularly when compared with their male counterparts [14], highlighting the need for a systematic approach to synthesising existing research on these performance attributes.

Despite the strides made in professionalisation of women’s football, the variation in competitive levels across leagues globally presents challenges in drawing reliable comparisons of physical performance. For example, match running performance across four seasons was substantially higher in the Spanish first division compared with the second division [15]. Further, large disparities in financial investment and revenue generation between competition levels globally likely impact player professionalism and physical qualities. As an example, in the English Women’s Super League (WSL), the average revenue for clubs was GBP £4 million in the 2022–2023 season [16]. However, the top four revenue-generating clubs accounted for 66% of the total revenue, and there was a 16-fold difference between the lowest and highest revenue-generating clubs [16]. This variation in overall professionalism extends not only between elite and non-elite leagues but also within different tiers of competition, complicating the ability to benchmark performance standards [7, 13]. This issue of participant classification extends across all sports. Recently, a Participant Classification Framework proposed by McKay et al. [17] was developed that categorises athletes according to their training status and competitive calibre, helping to address these classification challenges. This framework has seen some application in recent women’s football research [18, 19]; however, a systematic review and/or meta-analysis using such a classification to understand physical qualities across performance levels in women’s football has yet to be undertaken.

Therefore, the primary aim of this study was to conduct a systematic review and meta-analysis to establish normative data for the physical performance profiles of women’s football players. A secondary aim was to examine the influence of performance classification using a modified version of the Participant Classification Framework [17]. By synthesising the available research, this research aims to provide practitioners, researchers and governing bodies with critical insights into the typical physical qualities of women’s football players across the competitive spectrum. Moreover, this research seeks to identify key differences in physical performance on the basis of player classification, thereby offering valuable data to inform training, talent identification and player welfare policies globally. The data obtained from this analysis are accessible through a user-friendly web-based application (https://fifawomensdevelopmentprogramme.shinyapps.io/FIFA_Womens_Profiling/), enabling practitioners to benchmark the physical performance qualities of their athletes against the findings of this study. In this review, ‘female’ refers to biological and physiological characteristics, while ‘women’ is used in social and gender-related contexts.

## Methods

### Registration and Protocol

This systematic review and meta-analysis adhered to the Preferred Reporting Items for Systematic Reviews and Meta-Analyses (PRISMA) guideline to ensure transparency [20], alongside the consensus statement for reviews in Exercise, Rehabilitation, Sport Medicine, and Sports Science (PERSiST) [21]. The review was prospectively registered in the Open Science Framework (OSF) 14 April 2023 (available at: https://osf.io/8w3jh). Checklist items are available in Supplementary Material 1 (Supplementary Table S1).

### Research Question and Search Strategy

The research question for this project was developed in collaboration with FIFA, following the identification of a significant gap in accessible normative data on the physical qualities of women’s football players. In 2021, FIFA launched the Women’s National Team Preparation Programme to provide evidence-based support for Member Associations in the physical preparation of female players for major international tournaments. However, the lack of comprehensive data made benchmarking across different competitive levels challenging. While attempts of collating data from the research had previously been made, the absence of systematic framework for organising data, along with the ambiguity regarding the competition level from which the data were collected, highlighted the requirement of this project. Between September 2022 and March 2023, several expert group meetings refined the project concept. On the basis of these consultations and preliminary search results, seven primary physical qualities were selected. A detailed outline of the individual assessment protocols, procedures, instruments and technologies is presented in Table 1 and Sect. 2.5. These physical performance capacities and corresponding assessment protocols were identified as essential components of a standard assessment battery for female players across FIFA member associations. The protocols were selected with consideration of the varying resources and access to technologies available to different associations, ensuring that the assessments could be feasibly implemented across diverse settings.

**Table 1:** The physical qualities, protocols, procedure, instrument and/or technology that were extracted

Physical quality	Protocol	Procedure, instrument and/or technology
Cardiorespiratory fitness	Maximal oxygen uptake	Laboratory treadmill Field-based portable metabolic cart
	Maximal aerobic speed	Laboratory treadmill Set time-trial (e.g. 6, 12 min) Set-distance trial (e.g. 1.2, 2 km) VAM-EVAL
	Field-based intermittent protocols	Yo-Yo Intermittent Recovery Test Level 1 Yo-Yo Intermittent Recovery Test Level 2 30–15 Intermittent Fitness Test
Acceleration time	5 and up to 15 m test (including yards)	Timing gates, laser, GPS, video analysis, stopwatch
Sprint time	20 and up to 40 m test (including yards)	Timing gates, laser, GPS, video analysis, stopwatch
Change of direction	5–0–5 test Illinois test *T*-test	Timing gates, laser, video analysis, stopwatch
Maximal velocity	Testing Training/competition observations	Timing gates, laser, GPS, video analysis
Lower limb strength	Measured or estimated (e.g. velocity and weight)	1RM back squat, 1RM deadlift
Lower limb power	Squat jump/countermovement jump (height) and broad jump (distance)	Force plate, optical device, contact mat, video analysis, tape measure, video analysis (e.g. mobile apps)

*GPS* global positioning system, *1RM* one repetition maximum

Two authors (Compton and Shushan) developed the search strategy, using a combination of relevant free-text terms. The systematic review accelerator tool [22], including the word frequency analyser, search refinery and polygon search, were then utilised to identify the most relevant terms, optimise search queries, and ensure comprehensive coverage of literature. Keywords included football, soccer, ‘football player*’, ‘soccer player*’, ‘football athlete*’, ‘soccer athlete*’, female, woman, women, lady, ladies, performance, physical, endurance, aerobic, cardio*, anaerobic, fitness, stamina, capacity, sprint*, agility, speed, strength, power, force, jump*, and plyo*. These terms were linked using the Boolean operators ‘OR’ and ‘AND’. The electronic databases PubMed, Scopus, SPORTDiscus and Web of Science were first searched on 22 April 2023, with a filter applied to include studies published from 2003 onwards to capture research from the last two decades. An updated search was conducted on 23 May 2024. In addition to these searches, we incorporated secondary search sources, including screening reference lists of studies and reviews, tracking additional publications using social networks (e.g. ResearchGate and X) and regular Google Scholar checks from the updated search to 1 August 2024. Detailed descriptions of the search strategy and results are provided in Supplementary Material 2.

### Screening and Study Selection

**Fig. 1:**
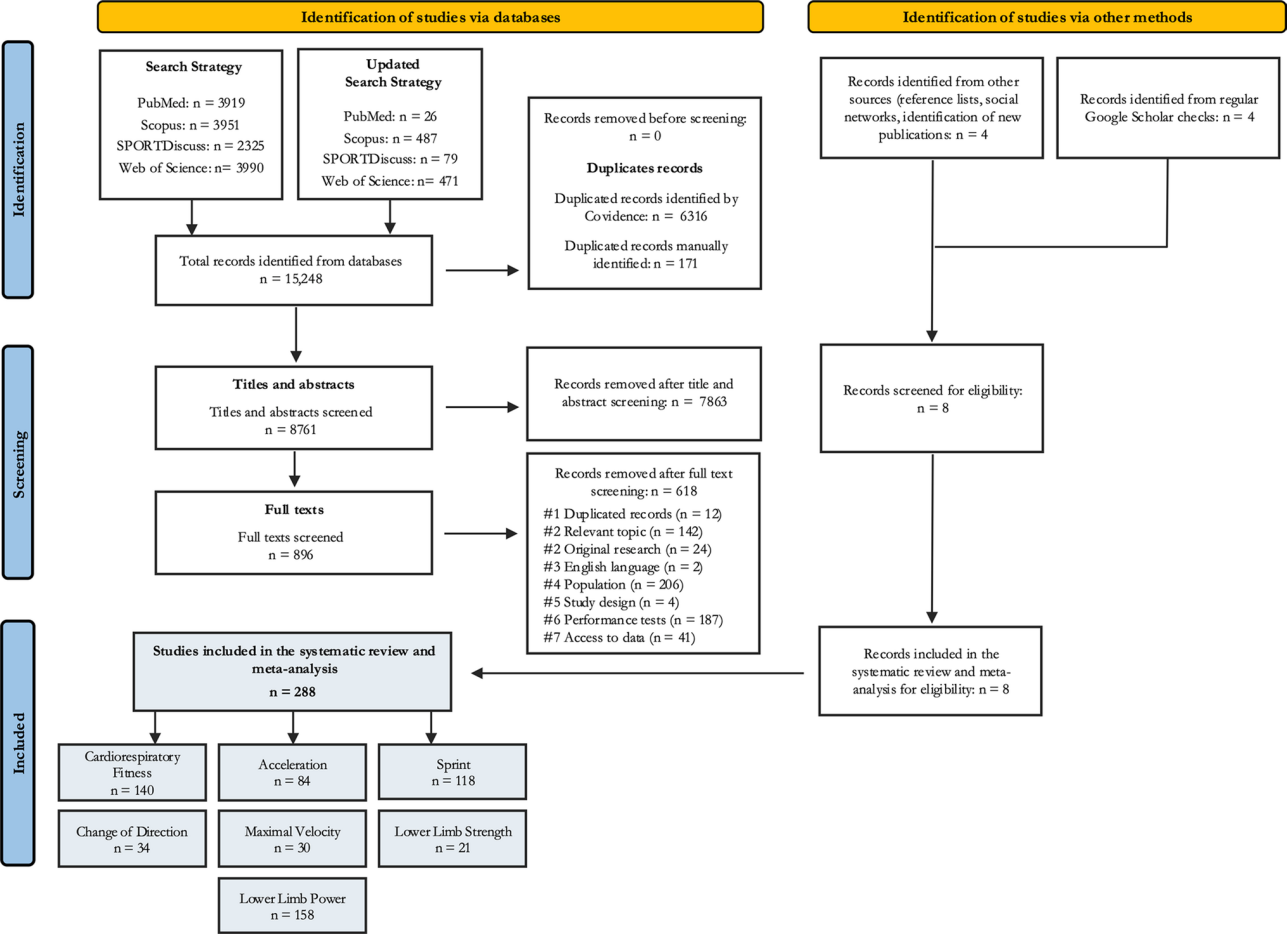
Preferred Reporting Items for Systematic reviews and Meta-Analyses (PRISMA) flow chart. The total number of studies included in the systematic review and meta-analysis was 287

Records from databases were downloaded to the reference management software EndNote (version 20.2.1.15749) and then imported to Covidence (Melbourne, Victoria, Australia) for initial screening. After excluding duplications, two authors (Compton and Shushan) screened titles and abstracts to determine eligibility. The full-text screening phase was conducted using a designated Excel spreadsheet. The same authors screened an approximately equal proportion of these studies using inclusion–exclusion criteria based on the Population, Intervention, Comparison, and Outcome (PICO) model, including the following conditions: (1) original research published in an English peer-reviewed journal; (2) full-text was accessible online or via direct request from the corresponding authors (3) in accordance with the most updated database searches (23 May 2024) or identified from secondary search methods up until 1 August 2024; (4) population included female football (soccer) players with a minimum mean group age of 16 years and no current medical condition or injury. While no definitive age criterion exists in literature, 16 years was deemed appropriate on the basis of observed physical development trends in female players [23] and to align with a comprehensive scoping review in women’s football [14]. Furthermore, an internal FIFA report highlighted that, compared with males, a significantly higher proportion of female players aged 16 years already compete in senior competitions. No restriction for competition level was applied as well as (5) no restriction for training interventions, except for studies incorporating acute interventions that may affect performance (e.g. acute supplementation, heat exposure, post-activation-potentiation, training-induced fatigue state). In such studies, data at baseline (pre-intervention) or control groups were included. All study designs were considered. Of note, studies incorporating chronic training interventions (e.g. strength and power, high-intensity interval training, pre-season training) or studies assessing physical performance in various female hormonal states (e.g. different phases of the menstrual cycle) were included. These studies reflect realistic conditions for data with female athletes, which may occur during different season phases, physical capacities and stages of the menstrual cycle in individual athletes. (6) Quantitative data (means and standard deviations [SDs]) from physical performance assessments were also included. The phases of searching and screening processes are shown in Fig. 1. A list of the excluded studies with justifications is available in the data repository (https://osf.io/d8twf/) in the Additional Files folder.

### Data Extraction and Coding

The following data were extracted from the included studies:

1. Study characteristics: lead author, year of publication, journal, title, group and effect size identification.
2. Population characteristics: sample size, mean age, age phase (developmental [e.g. U18, U20 squads], senior team), country, participant performance tier details as reported in the paper or provided by corresponding authors (Sect. 2.6) and playing position details (where available).
3. Study design (test–retest, correlational, observational with a single measure, observational with repeated measures, pre–post interventions, other) and main objectives.
4. Season phase: the phase of the season in relation to conducting the assessment.
5. Physical quality: assessment protocol and procedure, measurement unit, technology brand and details, surface, and additional notes.
6. Descriptive data: means and SDs of performance outcomes.

In this review, ‘estimate’ refers to the total data points collected across all studies, physical qualities and protocols, corresponding to the dataset’s total number of rows. ‘Observation’ accounts for the number of athletes contributing to each estimate, reflecting the dataset’s scale.

Supplementary Material 3 provides a detailed overview of the characteristics of all included studies. To ensure accuracy in the data extraction process, an independent cross-referencing screening of the main extracted data was performed on a random sample of 100 studies (approximately 350 data points) by four authors (Compton, Shushan, Lovell and Clubb). The inter-rater reliability, measured by percentage agreement and Cohen’s Kappa, was calculated as 92% and 0.92, respectively. Any discrepancies in the extracted data were discussed and resolved collaboratively to maintain consistency and precision.

While the authors (Compton and Shushan) endeavoured to extract as much relevant information as possible from the included studies, certain limitations should be acknowledged. In some cases, assumptions were made regarding variables that, while not directly impacting the analysis, provided important contextual information where explicit details were unavailable. These variables included: country, age phase, study design and season phase. Assumptions were made on the basis of indicators such as the location and affiliations of the authors, the involvement of university ethics committees, the timeframe of data collection and the reported mean age of athletes.

### Physical Performance Qualities

#### Cardiorespiratory Fitness

Cardiorespiratory fitness was assessed and characterised using both laboratory and field-based protocols. Only direct (laboratory) measurements of maximal oxygen consumption ($\dot{V}$ O_2_ max) were recorded. $\dot{V}$O_2_ max was attained during graded maximal exercise tests using treadmills (135 estimates/96 groups/63 studies), cycle ergometers (4/4/4), a field-based portable metabolic cart (1/1/1), or unspecified equipment (3/2/2). $\dot{V}$O_2_ max was determined from continuous spirometry, and all values were expressed relative to body mass (ml·min^−1^·kg^−1^). The protocols varied in starting velocities, step or incremental intensity methods, measurement tools (e.g. gas analysers) and analysis techniques. While this variability may be considered a limitation, it allowed for the inclusion of a larger body of existing research.

Maximal aerobic speed (MAS) was estimated using both direct laboratory measurements and indirect field-based protocols, with all results expressed in kilometres per hour (km·h^−1^). Direct MAS measurements were determined from the velocity attained during graded maximal exercise tests (_V_$\dot{V}$O_2_ max; 28/24/8). Many studies did not specify whether _V_$\dot{V}$O_2_ max was calculated as the lowest velocity at which $\dot{V}$O_2_ max was reached or final velocity at exhaustion. Indirect estimations of MAS from field-based protocols varied, including fixed-distance time trials (1200 m, 2/2/1; 1600 m, 2/1/1), fixed-time distance trials (12 min [Cooper test], 3/2/2), and the VAM-EVAL protocol (1/1/1; although removed from meta-analysis). In addition, these protocols differed in assessment formats, with some involving shuttle running and others using rectangular or circular tracks. Despite the variability in programming variables, we chose to meta-analyse them within a single dataset to provide practitioners with a practical range of estimated physical performance data from these low-cost, proxy assessments.

Field-based intermittent protocols included the Yo-Yo Intermittent Recovery Test Level 1 (YYIRL1; 146/107/66) and Yo-Yo Intermittent Recovery Test Level 2 (YYIRL2; 16/11/7), with the results reported as total distance achieved (m). For the 30–15 Intermittent Fitness Test (30–15_IFT_; 44/32/11), the final velocity (V_IFT_, expressed in km h^−1^) was recorded. Several authors were contacted to verify the test protocols (e.g. YYIRL1 and YYIRL2) and results to clarify outlier data presenting within the database. In cases where authors confirmed the accuracy of the protocols and results, data were retained; if discrepancies were identified, the database was corrected accordingly.

#### Acceleration Time

Acceleration time was assessed over distances of 5, 10 and 15 m, as well as 5, 10 and 15 yards (yd), with data reported in seconds. When studies reported mean velocity over these distances, values were converted to time (seconds). Acceleration measurements were obtained using either timing gates (271 estimates/197 groups/81 studies) or radar systems (7/5/3).

#### Sprint Time

Sprint time performance was assessed over distances of 20, 25, 30 and 40 m, as well as 20, 25 and 40 yd, with data presented in seconds. When studies reported mean velocity over these distances, the values were converted to seconds. Sprint measurements were obtained using timing gates (404 estimates/197 groups/105 studies), radar systems (3/3/2) and video analysis (3/2/2). Studies that used stopwatches (*n* = 7) were excluded from the meta-analysis owing to their presentation as outliers in the dataset.

#### Change of Direction

The 5–0–5 (33 estimates/21 groups/16 studies), Illinois agility (22/13/9) and T-test (12/8/7) protocols were selected to assess change of direction (COD) performance. During screening and data extraction, the T-test was included retrospectively given its prevalence in literature. As a result, studies initially screened for the 5–0–5 or Illinois agility tests, or those containing agility-related keywords in the title, abstract or notes, were re-screened to extract T-test data if available. These protocols were chosen in preference to others (e.g. arrowhead) owing to their higher prevalence in the initial screening and their more standardised testing procedures in terms of distance and methodology compared with other COD assessments. For all protocols, if studies reported separate data for dominant and non-dominant turns, the results were aggregated. Timing gates were the primary technology used to measure COD performance (66 estimates/39 groups/29 studies), while studies using stopwatches (*n* = 3 studies) were excluded from the meta-analysis.

#### Maximal Velocity

Maximum velocity was recorded during either discrete testing sessions (39 estimates/31 groups/18 studies) or through observations during training (1/1/1) or competition (34/33/12). For discrete testing, measurements were taken during 30–60 m maximal effort sprints. The technology used included global and local positioning system (GPS and LPS, respectively) devices (42/38/15), timing gates (21/18/11), foot-mounted accelerometers (2/1/1), radar devices (7/6/3) and video analysis (MySprint App; 2/1/1). Of the GPS- and LPS-enabled devices used, only one of these recorded at 5 Hz (4/4/1; although interpolated to 15 Hz) and the remaining recorded at 10 Hz. The MySprint App was considered appropriate for assessing maximal velocity and sprint performance, as it demonstrated almost perfect agreement with reference tools such as timing gates and radar devices [24]. To obtain pooled estimates for each testing procedure (measured and observed maximal velocity), these categories were analysed separately, with test procedure included as a dichotomous moderator in the baseline meta-analysis model.

#### Lower Limb Strength

Lower limb strength assessments included one repetition maximum (1RM) protocols, including the back squat (44 estimates/32 groups/21 studies) and deadlift (1/1/1; although removed from meta-analysis owing to limited data). Strength assessments included either true 1RM measurements, estimation based on multiple repetitions (e.g. 3RM or 5RM), or derived from force–velocity profiling. Data reported in pounds were converted to kilograms. Although we initially considered analysing studies with performance outcomes from multiple repetitions, this information was infrequently reported and primarily used to estimate 1RM. No other strength assessments, such as maximal isometric contractions (e.g. isometric mid-thigh pull and groin squeeze) or isokinetic protocols, were included after thorough discussion among the authors and consideration of preliminary search findings. Excluding these protocols minimised variations in testing procedures (e.g. body/joint position, angle, and velocity), technology, and derived metrics, which could introduce confounding factors. Furthermore, many of these excluded studies fell outside the scope of this review, such as those focussed on acute interventions (e.g. post-match recovery kinetics) or those conducted among athletes in rehabilitation or with medical conditions (e.g. lower-limb injury). By focusing solely on 1RM assessments, this study aims to provide practitioners with readily applicable and easily reproducible protocols that do not require extensive equipment.

#### Lower Limb Power

Lower limb power measures included the squat jump (SJ; 94 estimates/63 groups/44 studies) height, countermovement jump (CMJ; 366/237/151) height and broad jump (BJ; 67/43/19) distance, all recorded in centimetres (cm). No other jump-derived metrics, such as power calculations, were included owing to the extensive range of metrics available, differing methods of estimation and potential challenges in interpretability. To maintain the study’s focus on providing practical and easily reproducible testing data, only jump height and distance were considered.

The technology used to measure SJ and CMJ performance included (1) optical devices (e.g. Optojump), contact mats (e.g. Chronojump) and mobile apps for video analysis (e.g. MyJump; 359/228/135); and (2) force plates (96/73/52). For analysis, the first group was collectively categorised as ‘optical or contact devices’. Two testing protocols were used for SJ and CMJ: restricted arm movement (hands fixed on hips, or in some cases on shoulders with a wooden dowel), and unrestricted arm movement (e.g. arm swing [Abalakov jump]). Data were meta-analysed separately for these categories. Studies using manual methods (e.g. Vertec device, wall measurements) were excluded from the meta-analysis. The BJ was recorded using a tape measure and required arm swing. Studies that did not report testing procedures or technology were followed up with author contact. If information was not provided, the study was excluded from the meta-analysis. Several authors were contacted to verify testing protocols or results that appeared as outliers in the dataset, with a small number providing updated data.

### Participant Tier Classification

The performance level (tier) of the participants within the included studies was categorised into four unique tiers (Tier 2 to Tier 5) using a modified version of the Participant Classification Framework by McKay et al. [17] (Table 2). This modification was specifically designed for this systematic review and meta-analysis after discrepancies were identified during data coding, where the original framework did not accurately reflect the context of women’s football. After multiple meetings between the authors, consultations with technical-tactical and performance experts from the FIFA Women’s Football Development Program and incorporating objective data from FIFA’s latest benchmarking project of global league rankings, minor adjustments were made to the framework. The key modification involved elevating the top professional women’s football leagues, namely the English Women’s Super League, American National Women’s Soccer League, Spanish Primera División de la Liga, French Première Ligue, and German Frauen-Bundesliga, into Tier 4, even when teams competed solely at the national level. In addition, senior national teams that reached the quarterfinals of the FIFA World Cup or Olympics were classified as Tier 5. For studies where this information was not explicitly stated, efforts were made to align data collection with the performance of national teams in the most recent tournament, either by the year of data collection (when specified) or publication. A substantial number of eligible studies lacked the necessary information to categorise their participant’s tier. To address this, the authors were contacted to clarify the performance tier (as outlined in Table 2). If no response was received or the information provided was insufficient, the authors discussed and reached a consensus on the appropriate tier classification. If consensus could not be achieved, the study was excluded from the meta-regression analyses.

**Table 2:** The modified framework that was used to classify participants’ performance level on the basis of the Participant Classification Framework previously established by McKay et al. [17]

Tier 2	• Groups competing in regional or local leagues/tournaments
	• Typically train ≤ 3 times for a single match during a typical training week
Tier 3	• Groups competing in national leagues, excluding the most professional senior leagues worldwide
	• Typically train > 3 times for a single match during a typical training week
	• Groups competing at the NCAA Division II and III levels
Tier 4	• Groups competing in the most professional senior leagues worldwide
	• Groups competing in international club tournaments
	• Groups belonging to the national team squads
	• Groups competing at the NCAA Division I level
Tier 5	• Groups belonging to the senior national team squads who made the Women’s FIFA World Cup quarter final stage
	• Groups belonging to the senior national team squads who made the Women’s Olympics quarter final stage

*NCAA* National Collegiate Athletics Association

### Handling Missing Data

For studies with missing or unclear information (e.g. testing procedures and technology), corresponding authors were contacted via email or social networks (e.g. ResearchGate, X and LinkedIn) to request the necessary data. These requests pertained to sample characteristics, including sample size, age, and country, as well as clarification on the participant tier, assessment protocols, technology used and descriptive data (means and SDs) from physical performance assessments. In total, 194 authors (representing separate studies) were contacted. If no response was received within a reasonable timeframe (approximately 4 weeks), a first follow-up was sent. If still no response was received, a second follow-up was sent, often including co-authors. We received responses from 107 authors, with 104 of these providing either additional information or full raw data. In cases where no response was received and the study lacked critical information, the study was excluded. For studies where descriptive data were only presented in figures (eight studies), graph digitiser software [22] was used to extract means and SDs. In cases where only means and standard error (SE) were reported, SE was converted to SD by multiplying it by the square root of the sample size. If the SE was not available, authors were contacted for SD or SE; studies were excluded if no response was received. In some instances, discrepancies arose between the original reported sample size and the sample size reported for physical performance observations, often due to players being unable to complete the entire test battery. When updated sample characteristics (e.g. mean age) were unavailable, the original reported information was used.

### Risk of Bias Assessment

Risk of bias was assessed using a modified Downs and Black [25] scale, adapted in line with previous comparable research [26]. To outline the key reporting criteria domains and scale modifications, we incorporated the STrengthening the Reporting of OBservational studies in Epidemiology (STROBE) checklist [27]. The modified scale and summary criteria are detailed in Supplementary Material 4. From the original 27 Downs and Black criteria, 10 were selected on the basis of their relevance to studies included in this review, corresponding to four key domains: reporting (1–3, 6), external validity (11), internal validity (15, 20) and selection bias (22, 26). Responses were rated as ‘yes’, ‘no’, ‘N/A’ or ‘unable to determine’. Modifications were made to the original scale to accommodate the diverse study designs in this review, with the majority including observational studies. Therefore, to ensure a comprehensive and systematic assessment of bias relevant to our aims (synthesising physical quality performance outcomes), the integration of the Downs and Black and STROBE checklist was deemed appropriate.

### Statistical Methods and Data Analysis

All data analyses were performed using R Studio (version 4.3.0) [28] utilising the *metafor* [29] *clubSandwich* [30], and *orchaRd* [31] packages. Data wrangling and visualisations were conducted with the *tidyverse*, *KableExtra*, *formattable*, and *ggplot2* extension packages. A detailed outline of the statistical processes and the code used for conducting the analysis for one specific protocol (20-m sprint) are available in Supplementary Material 5. Further, code for all protocols is available via OSF (https://osf.io/d8twf/).

To provide practitioners with practical tools for comparing the performance of their players against the results of this benchmarking systematic review and meta-analysis, meta-analyses were conducted on both means and SDs. Including SDs in the analysis offers insights into the between-athlete variability (dispersion) in performance outcomes, in addition to central tendency. By pooling estimates for both means and SDs, practitioners can use common tools in the field (e.g. *Z*-scores, standardised 1–10 [STEN] scores, and percentiles) to make informed decisions on profiling, norm comparisons and within-individual training effects, all while considering uncertainty in the meta-analysis. Meta-analyses of means were conducted using raw data, with the square of the standard error (derived from SD and sample size) representing the sampling variance [29]. For SD meta-analyses, effect estimates were log-transformed, with sampling variance calculated on the basis of sample size [32, 33]. Pooled estimates were then back-transformed to their original values for ease of interpretation, with a bias correction applied for sample size.

Many studies provided multiple effect estimates from distinct groups (e.g. repeated measures and pre- and post-intervention data) or subgroups (e.g. different competition levels, position-specific groups or intervention versus control groups). Given the hierarchical (nested) nature of the data and potential for statistical dependency, particularly within-group repeated measures, data were analysed using multi-level mixed-effects meta-analysis models combined with robust variance estimation, clustering at the study level [34, 35]. This approach allows for the assessment of variance across multiple levels [36], offering a robust method to account for the dependency of effect estimates derived from common samples [37]. In cases where studies reported multiple effect estimates from same group, we addressed the dependency by incorporated the full variance–covariance matrix of the estimates (‘V matrix’) in place of sampling variance [35]. Since most studies did not report the correlations between effect estimates from the same group, we assumed a constant correlation (*ρ*) of 0.7 for the analysis. A minimum of three studies per physical performance outcome was required for inclusion in the meta-analysis. Initial (baseline) models for physical performance outcomes were fitted using restricted maximum likelihood method, with coefficient based on the *t*-distribution.

Uncertainty in meta-analyses was expressed using 90% compatibility (confidence) intervals (CIs) and 90% prediction intervals (PIs). The CIs indicate the range of performance outcomes compatible with our models and assumptions, whereas the PIs provide insight into the likely range of performance outcomes in a new, similar study [38]. Heterogeneity in individual analyses was assessed at all levels (study, group, and effect size) using Sigma (*σ*), expressed in SD units, and the *I*^*2*^ statistic, which represents the proportion of total variability attributable to true differences rather than sampling error [39, 40]. These data are presented in Supplementary Material 6.

To address the secondary objective of this study, the moderating effects of participant tier were evaluated through meta-regression, yielding tier-specific mean and SD (where applicable). Studies were coded according to their tiers (Sect. 2.6). Moderating effects were incorporated into baseline models when there were at least ten effect estimates and six independent samples in each tier level [41, 42]. For each tier, mean and SD values were calculated to provide a clear benchmark for performance at different competitive levels. Owing to the limited number of studies involving Tier 5 participants, Tiers 4 and 5 were pooled for the meta-regression analysis. To interpret the differences between tiers, rather than relying on traditional null hypothesis significance testing and dichotomising results based on the presence or absence of an effect, the moderating effects were interpreted across the entire range of the CIs. This approach focused on the point estimates, reflecting values consistent with our models and assumptions.

Although anchor-based values are often preferred for evaluating group differences or individual training effects in physical performance owing to their ecological validity and relevance to operational outcomes [43], they pose several challenges. These include the substantial variation in the proposed thresholds for ‘real-world’ changes (e.g. 1–5% in sprint time [44, 45]) and a lack of empirical evidence validating such thresholds. In addition, the determination of these thresholds often involves subjective decisions, potentially introducing bias and reducing the objectivity of their application [43]. Moreover, comparable anchor-based values for several key performance outcomes (e.g. $\dot{V}O_2$ max and set distance/time-trial protocols) were not available for this meta-analysis. Given these limitations, we opted to assess meta-regression comparisons as standardised mean differences, referenced against the pooled between-individual SD with qualitative effect size descriptions. Thresholds of > 0.2, > 0.6, and > 1.2 were used to denote small, moderate, and large effect sizes, respectively [46]. Preliminary analysis with proposed thresholds in literature indicated that these values align with the range of our comparisons (typically around a moderate effect size). Effects were deemed substantial when the CIs predominantly or entirely extended beyond the trivial range. If the CIs spanned both trivial and substantial regions in one direction, the effect was classified as both trivial and substantial. Conversely, when the CIs overlapped with substantial regions in both positive and negative directions, the effect was deemed inconclusive.

Small-study effects and potential publication bias were visually assessed using counter-enhanced funnel plots [47], presented in Supplementary Material 7. To complement visual inspection, Egger’s regression test with precision was conducted [47], incorporating the inverse standard error of the sampling variance as a moderator [48]. This approach accounts for study size effects, with larger studies contributing greater weight to the regression model [49]. For sensitivity analysis, we followed recent recommendations [35], testing a range of plausible correlation values (*ρ* = 0.3, 0.5 and 0.9), alongside the selected correlation (0.7), to assess the impact of within-group covariance changes on pooled estimates and variance components (Supplementary Material 8). These analyses consistently yielded identical pooled estimates and uncertainty.

## Results

### Study Characteristics

After screening, the final dataset included 1855 estimates from 982 groups across 288 studies, spanning seven physical capacities (Fig. 1). The distribution of studies included for each physical capacity was as follows: cardiorespiratory fitness (*n* = 140 studies, 49%), acceleration (*n* = 84, 29%), sprint performance (*n* = 118, 41%), COD (*n* = 34, 12%), maximal velocity (*n* = 30, 10%), lower limb strength (*n* = 21, 7%) and lower limb power (*n* = 158, 55%). Collectively, these studies represented an overall sample size of 18,722 athletes, contributing 32,795 individual observations. A summary of all included studies is displayed in Supplementary Material 3 (Supplementary Table S3). To facilitate interpretation of these extensive datasets, we developed an interactive dashboard that allows users to explore the descriptive data for each physical capacity, review key findings and access practical applications for benchmarking [50].

### Overall Meta-Analyses

Tables 3, 4, 5, 6, 7, 8, 9 provide a summary of the overall meta-analysis results for each physical capacity and their respective assessment protocols, including the pooled mean, pooled SDs and corresponding 90% CIs and PIs. Owing to limited data, meta-analyses could not be conducted for certain protocols, including VAM-EVAL; 5-yd, 10-yd, and 15-yd acceleration; 25-yd sprint; 1RM deadlift for lower limb strength; and SJ with unrestricted arm movement performed using both optical/contact devices and force plates for lower limb power. Notable heterogeneity was observed across all models and estimates, with most of this heterogeneity occurring at the study level (i.e. differences in effect sizes between studies), as detailed in Supplementary Material 6 (Supplementary Table S5).

**Table 3:** Meta-analysis of cardiorespiratory fitness

Physical performance	Number of:			Pooled mean			Pooled SD	
	Estimates	Groups	Studies	Estimate	90% CIs (lower to upper)	90% PIs (lower to upper)	Estimate	90% CIs (lower to upper)
$\dot{V}$O_2_ max	135	96	61	49.0	47.6 to 50.4	39.7 to 58.3	4.1	3.7 to 4.5
YYIRL1	146	107	66	1143	1060 to 1227	560 to 1727	275	248 to 305
*V*_IFT_	44	32	11	18.0	16.8 to 19.2	14.5 to 21.5	1.1	0.8 to 1.5
_*V*_$\dot{V}$O_2_ max	28	24	8	14.6	13.6 to 15.6	12.2 to 17.1	0.9	0.6 to 1.3
YYIRL2	16	11	7	446	331 to 560	134 to 757	91	60 to 139
Set distance/time trial	8	6	5	12.7	10.9 to 14.5	9.4 to 16.0	1.4	0.6 to 3.4

$\dot{V}$*O*_*2*_* max* maximal oxygen uptake (ml·min^−1^·kg^−1^), *YYIRL1* Yo-Yo Intermittent Recovery Test Level 1 (m), V_IFT_ final velocity attained during 30–15 Intermittent Fitness Test (km·h^−1^), _*V*_ $\dot{V}$ *O*_*2*_* max* final velocity attained during graded maximal exercise tests (km h^−1^), *YYIRL2* Yo-Yo Intermittent Recovery Test Level 2 (m), set distance/time trial (km h^−1^), *CIs* confidence intervals, *PIs* prediction intervals, *SD* standard deviation

**Table 4:** Meta-analysis of acceleration time

Physical performance	Number of:			Pooled mean			Pooled SD	
	Estimates	Groups	Studies	Estimate	90% CIs (lower to upper)	90% PIs (lower to upper)	Estimate	90% CIs (lower to upper)
5 m	72	53	28	1.18	1.13–1.24	0.95–1.42	0.07	0.05–0.08
10 m	167	129	75	1.99	1.96–2.01	1.77–2.20	0.09	0.09–0.10
15 m	22	13	8	2.81	2.59–3.03	2.77–3.36	0.10	0.08–0.14
5 yards	7	4	2	–	–	–	–	–
10 yards	4	4	1	–	–	–	–	–
15 yards	6	3	1	–	–	–	–	–

All estimates are expressed in s

*CIs* confidence intervals, *PIs* prediction intervals, *SD* standard deviation

**Table 5:** Meta-analysis of sprint time

Physical performance	Number of:			Pooled mean			Pooled SD	
	Estimates	Groups	Studies	Estimate	90% CIs (lower to upper)	90% PIs (lower to upper)	Estimate	90% CIs (lower to upper)
20 m	157	106	58	3.45	3.40–3.50	3.12–3.79	0.15	0.13–0.17
25 m	16	7	3	4.13	3.84–4.42	3.69–4.58	0.12	0.09–0.15
30 m	157	113	65	4.88	4.81–4.95	4.39–5.37	0.21	0.19–0.22
40 m	54	38	23	6.11	5.99–6.24	5.61–6.62	0.27	0.25–0.31
20 yards	7	6	3	3.30	3.10–3.50	3.02–2.57	0.09	0.02–0.42
25 yards	6	3	1	–	–	–	–	–
40 yards	24	9	4	5.62	4.65–6.59	4.01–7.23	0.24	0.09–0.63

All estimates are expressed in s

*CIs* confidence intervals, *PIs* prediction intervals, *SD* standard deviation

**Table 6:** Meta-analysis of change of direction time

Physical performance	Number of:			Pooled mean			Pooled SD	
	Estimates	Groups	Studies	Estimate	90% CIs (lower to upper)	90% PIs (lower to upper)	Estimate	90% CIs (lower to upper)
5-0-5	33	21	16	2.57	2.50–2.64	2.33–2.82	0.13	0.11–0.15
Illinois	22	13	9	17.88	17.20–18.57	16.13–19.64	0.62	0.45–0.85
*T*-test	12	8	7	11.47	10.29–12.66	8.84–14.11	0.58	0.40–0.83

All estimates are expressed in s

*CIs* confidence intervals, *PIs* prediction intervals, *SD* standard deviation

**Table 7:** Meta-analysis of maximal velocity

Physical performance	Number of:			Pooled mean			Pooled SD	
	Estimates	Groups	Studies	Estimate	90% CIs (lower to upper)	90% PIs (lower to upper)	Estimate	90% CIs (lower to upper)
Measured	39	31	18	26.2	24.7–27.7	21.7–30.7	1.7	1.2–2.3
Observed	35	34	13	26.9	25.4–28.5	22.2–31.7	1.5	1.4–1.7

All estimates are expressed in kilometres per hour (km h^−1^)

*CIs* confidence intervals, *PIs* prediction intervals, *SD* standard deviation

**Table 8:** Meta-analysis of lower limb strength

Physical performance	Number of:			Pooled mean			Pooled SD	
	Estimates	Groups	Studies	Estimate	90% CIs (lower to upper)	90% PIs (lower to upper)	Estimate	90% CIs (lower to upper)
1RM back squat	44	32	21	87.4	78.4–96.3	52.0–122.7	13.7	11.5–16.3
1RM deadlift	1	1	1	–	–	–	–	–

*1RM* one repetition maximum (kg), *CIs* confidence intervals, *PIs* prediction intervals, *SD* standard deviation

**Table 9:** Meta-analysis of lower limb strength

Physical performance	Technology/instrument	Procedure	Number of:			Pooled mean			Pooled SD	
			Estimates	Groups	Studies	Estimate	90% Cis (lower to upper)	90% PIs (lower to upper)	Estimate	90% Cis (lower to upper)
SJ	Force plate	Restricted	14	12	11	27.6	24.9–30.3	20.1–35.1	3.3	2.3–4.6
		Unrestricted	–	–	–	–	–	–	–	–
	Optical or contact device	Restricted	75	47	29	28.9	27.2–30.6	21.2–36.6	3.7	3.4–4.0
		Unrestricted	2	1	1	–	–	–	–	–
CMJ	Force plate	Restricted	72	53	35	28.3	27.0–29.5	22.1–34.4	3.7	3.4–4.1
		Unrestricted	10	8	6	33.0	29.7–36.2	26.3–39.7	4.0	3.1–5.2
	Optical or contact device	Restricted	229	139	82	31.1	29.7–32.6	19.9–42.3	3.6	3.3–4.0
		Unrestricted	53	41	21	37.6	32.8–42.4	19–56.2	4.7	4.1–5.4
BJ	Tape	Unrestricted	63	41	18	189.3	180.4–198.2	157.3–221.3	11.7	9.7–14.1

All estimates are expressed in cm

*SJ* squat jump, *CMJ* countermovement jump, *BJ* broad jump, *CIs* confidence intervals, *PIs* prediction intervals, *SD* standard deviation

### Moderating Effect of Participant Tier

**Fig. 2:**
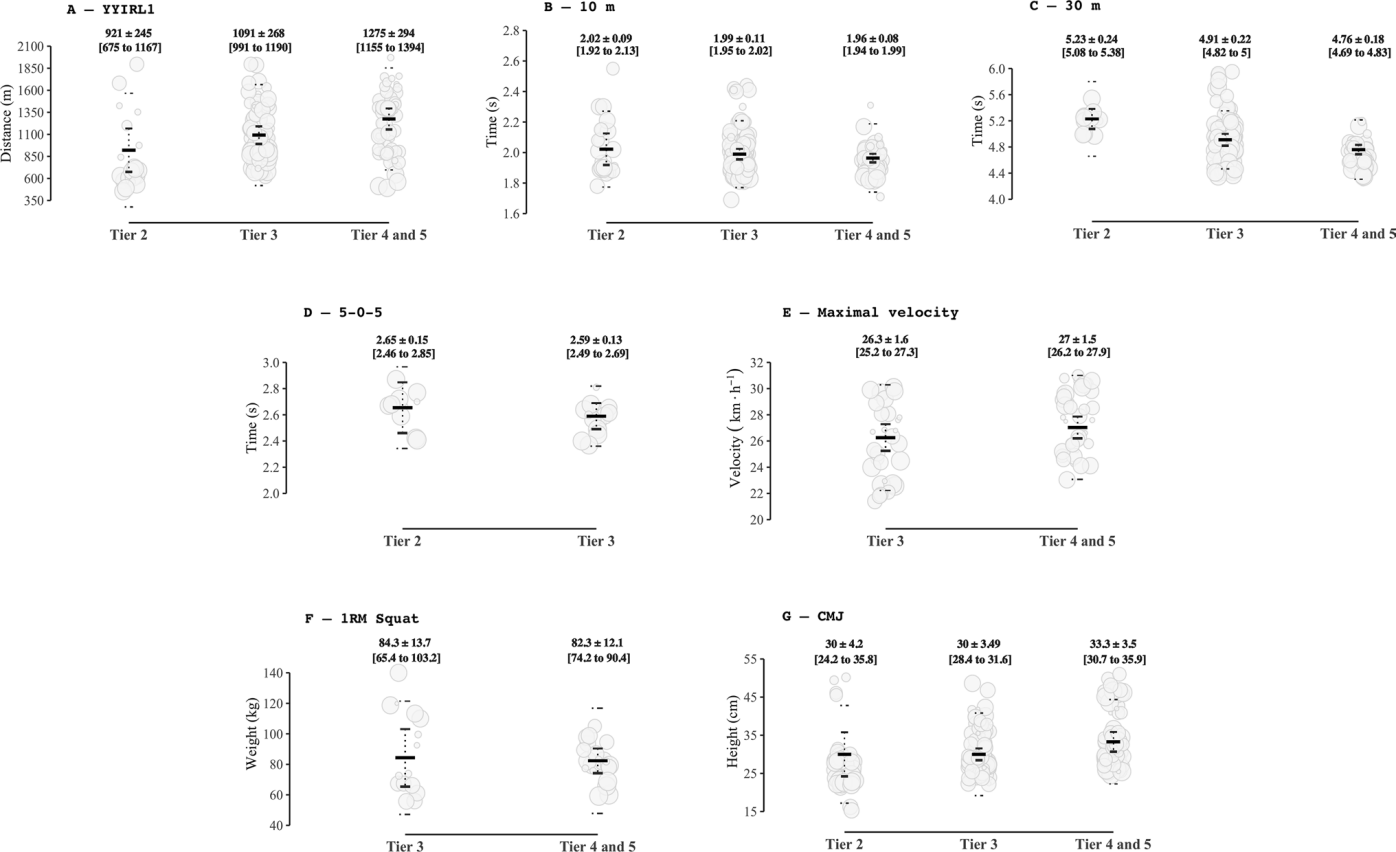
Bubble plots from mixed-effects meta-regression illustrating the moderating influence of participant tier on the model’s estimates. A single protocol was selected for each physical capacity to create the bubble plots (**A**–**G**). Data points represent individual effect estimates included in our meta-analysis, with their sizes reflecting their respective weights. Solid error bar lines represent the estimate of the modifying effect. Dashed and dotted lines represent 95% confidence and prediction limits, respectively. *YYIRL1* Yo-Yo Intermittent Recovery Test Level 1, *CMJ* countermovement jump

The moderating effect of participant tier was evaluated across multiple physical capacities, including three cardiorespiratory fitness tests ($\dot{V}$O_2_ max, YYIRL1 and V_IFT_), two acceleration tests (5 m and 10 m), two sprint tests (20 m and 30 m), one COD test (5–0–5) as well as maximal velocity and lower limb power (using two CMJ, two SJ, and BJ protocols). To enhance clarity while avoiding excessive detail, Fig. 2 presents meta-regression bubble plots for a single assessment from each physical capacity. All meta-regression results and visualisations are available via the interactive dashboard [50], with detailed model output statistics available in Supplementary Material 9 (Supplementary Table S7).

#### Cardiorespiratory Fitness

The moderating effects of participant tier on cardiorespiratory fitness performance were assessed for $\dot{V}$O_2_ max, YYIRL1 and V_IFT_. For $\dot{V}$O_2_ max, the comparison between Tier 3 versus Tiers 4 and 5 showed an inconclusive moderating effect (*b* = 0.8 ml min^−1^ kg^−1^ [90% CIs  − 2.1 to 3.7]; small). In contrast, substantial effects were observed for YYIRL1 performance (Fig. 2, Panel A), with large increases when advancing from Tier 2 to Tiers 4 and 5 (*b* = 354 m [90% CIs 141 to 567]) and moderate increases from Tier 3 to Tiers 4 and 5 (*b* = 184 m [90% CIs 66 to 302]). The comparison between Tier 2 and Tier 3 was compatible with both trivial and substantial effects (*b* = 170 m [90% CIs  − 25 to 364]; moderate). For V_IFT_, both a trivial and substantial (large) effect was observed when progressing from Tier 2 to Tier 3 (*b* = 2.0 km h^−1^ [90% CIs  − 0.2 to 4.2]; large), and a substantial effect was observed when comparing Tier 2 to Tiers 4 and 5 (*b* = 2.5 km h^−1^ [90% CIs 0.4 to 4.5]; large). The differences between Tier 3 and Tiers 4 and 5 were inconclusive (90% CI  − 1.9 to 2.9).

#### Acceleration Time

The moderating effects of participant tier on acceleration performance were assessed for both 5-m and 10-m assessments. Comparisons across Tier 2 and Tier 3 (*b* = 0.02 cm [90% CIs  − 0.11 to 0.14) and Tier 2 and Tiers 4 and 5 (*b* =  − 0.01 cm [90% CIs – 0.14 to 0.13) demonstrated inconclusive effects. The difference between Tier 3 and Tiers 4 and 5 was compatible with trivial and substantial effects (*b* =  − 0.03 s [90% CIs  − 0.04 to 0.00]; small). For 10-m acceleration (Fig. 2, Panel B), the progression from Tier 2 to Tier 3 (*b* =  − 0.03 s [90% CIs  − 0.12 to 0.05]) and from Tier 2 to Tiers 4 and 5 (*b* =  − 0.06 s [90% CIs  − 0.14 to 0.02]) also demonstrated inconclusive effects. However, the difference between Tier 3 and Tiers 4 and 5 was compatible with both trivial and substantial effects (*b* =  − 0.03 s [90% CIs  − 0.05 to 0.00]; small).

#### Sprint Time

The moderating effect of participant tier on sprint performance was assessed for both 20-m and 30-m distances. Progressing through the performance tiers demonstrated substantial effects across all comparisons, ranging from small to large. For the 20-m sprint, advancing from Tier 2 to Tier 3 resulted in a moderate reduction in time (*b* =  − 0.17 s [90% CIs  − 0.28 to − 0.06]; moderate), while the progression from Tier 2 to Tiers 4 and 5 had a larger effect.

(*b* =  − 0.22 [90% CIs  − 0.32 to − 0.11]; large). The comparison between Tier 3 and Tiers 4 and 5 showed a substantially small effect (*b* =  − 0.04 s [90% CIs  − 0.08 to − 0.01]; small). For the 30-m sprint (Fig. 2, Panel C), large effects were observed when transitioning from Tier 2 to Tier 3 (*b* =  − 0.32 s [90% CIs  − 0.46 to – 0.18]) and from Tier 2 to Tiers 4 and 5 (*b* =  − 0.47 s [90% CIs  − 0.60 to − 0.34]; large). The difference between Tiers 3 and Tiers 4 and 5 showed a moderate effect (*b* =  − 0.15 s [90% CIs  − 0.24 to − 0.05]; moderate).

#### Change of Direction

Sufficient data were available to assess COD performance using the 5–0–5 test for comparisons between Tier 2 and Tier 3 (Fig. 2, Panel D). The analysis showed an inconclusive moderating effect between these tiers (*b* =  − 0.06 s 90% CIs  − 0.20 to 0.07]).

#### Maximum Velocity

Owing to limited data, separate meta-regressions for measured and observed velocity testing procedures could not be conducted. However, since the overall (baseline) model showed no differences between the two test procedures, a meta-regression was conducted on the combined dataset (Fig. 2, Panel E). This allowed for the assessment of the moderating effect of competitive tier across Tier 3 and Tiers 4 and 5. The progression from Tier 3 to Tiers 4 and 5 was compatible with both a trivial and substantial effect (*b* =  − 0.8 km h^−1^ [90% CIs  − 0.1 to 1.7]; small).

#### Lower Limb Strength

The distribution of data across athlete tiers within the 1RM back squat dataset allowed for an evaluation of the moderating effect between Tier 3 and Tiers 4 and 5 (Fig. 2, Panel F). The meta-regression demonstrated an inconclusive effect when progressing from Tier 3 to Tiers 4 and 5 (*b* =  − 2.0 kg [90% CIs  − 17.5 to 13.6]).

#### Lower Limb Power

The moderating effects of participant tier on lower limb power were evaluated across several assessments and procedures: (1) SJ using optical or contact devices with restricted arm movement; (2) CMJ using optical or contact devices with both restricted and unrestricted arm movement; (3) CMJ using a force plate with restricted arm movement; and (4) BJ (Supplementary Material 9, Supplementary Table S7).

For the SJ measured via optical or contact devices and with restricted arm movement, advancing from Tier 2 to Tier 3 had an inconclusive effect on jump performance (*b* = 2.6 cm [90% CIs  − 1.1 to  6.3 cm]; moderate), while players classified in Tiers 4 and 5 demonstrated a substantial increase compared with both Tier 2 (*b* = 6.2 cm [90% CIs 2.7 to 9.7]; large) and Tier 3 (*b* = 3.6 cm [90% CIs 0.7 to 6.4]; moderate).

For the CMJ using an optical or contact device with restricted arm movement, the moderating effect was inconclusive when comparing Tier 2 with Tier 3 (*b* = 0.01 cm [90% CIs  − 4.9 to 4.9) and Tier 2 to Tiers 4 and 5 (*b* = 3.3 cm [90% CIs  − 3.6 to 10.2]; moderate). However, progressing from Tier 3 to Tiers 4 and 5 showed substantial increases in CMJ performance using both restricted (*b* = 3.3 cm [90% CIs 1.3 to 5.3]; moderate) and unrestricted (*b* = 8.8 cm [90% CIs 2.5 to 15.1]; large) arm movement procedures. For CMJ using a force plate with restricted arm movement, the differences between Tier 3 and Tiers 4 and 5 were trivial (*b* = 0.2 cm [90% CIs  − 3.1 to 3.4]). Finally, comparisons of the BJ across all tiers showed trivial effects on performance (Supplementary Material 9, Supplementary Table S7).

### Risk of Bias Assessment

The risk of bias assessment for all studies is available in the data repository (https://osf.io/d8twf/) in the Additional Files folder. Supplementary Material 10 (Supplementary Fig. S8) presents a summary of the overall distribution of responses (across all studies) for each domain. Of all studies, 100% clearly reported the study objectives, while 86% provided sufficient details on the testing protocols, procedures, and technology used, and 76% clearly reported the main findings (mean and SD). Areas that were identified as lacking transparency included the reporting of the validity and reliability of outcomes assessed, where 67% of studies did not report this information. In addition, 25% of studies did not provide sufficient details on athlete characteristics, requiring further clarification or follow-up with authors. Further, 36% of studies did not disclose funding sources or potential conflicts of interest.

### Benchmarking

**Fig. 3:**
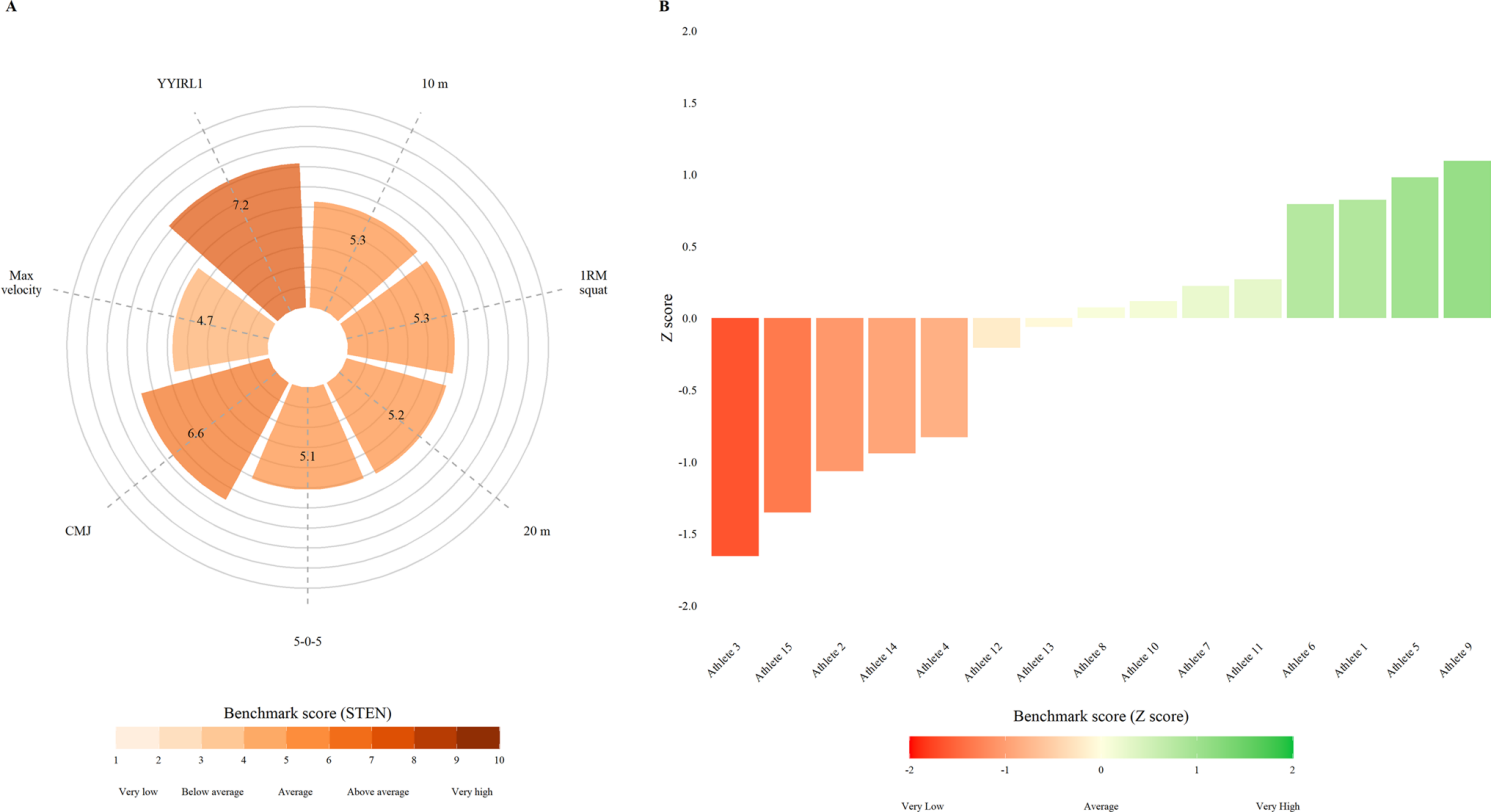
Example applications of meta-analysis data for benchmarking purposes in applied settings; **A** benchmarking scores presented at a team level. In this example, each team is compared against the meta-analysis data, and the benchmark is expressed as a standardised ten score (STEN; 1–10 scale); **B** benchmarking scores presented at an individual level. In this example, all athletes are compared against the meta-analysis data for a single protocol (i.e. 10-m acceleration), and the benchmark is expressed as a *Z*-score (− 2 to 2 standard deviations) *YYIRL1* Yo-Yo Intermittent Recovery Test Level 1, *CMJ* countermovement jump, *1RM* one repetition maximum

Figure 3 illustrates how the data from this systematic review and meta-analysis can be applied practically for benchmarking, with an interactive version available in the R shiny application [50]. Specifically, Fig. 3 (Panel A) shows a rose chart summarising team-level benchmarking (using a mock dataset), comparing the squad’s performance across assessments of seven physical qualities. These results are displayed using a STEN score, which compares the squad’s result against the meta-analysis pooled estimate and SD. The STEN score is calculated by converting a *Z*-score (reflecting how many SDs the result deviates from the mean) using the formula: STE*N* = 2 *Z* + 5.5. Each STEN unit corresponds to a SD of 0.5, with a score of 5.5 representing the mean (*Z*-score of 0). Scores below 5.5 indicate a ‘very low’ to ‘average’ performance relative to the meta-analysis data, while scores above 5.5 reflect ‘above average’ to ‘very high’ performance.

Figure 3 (Panel B) shows individual-level comparisons to the meta-analysis data using CMJ performance as an example. Individual athlete’s performances are presented using *Z*-scores, with positive values indicating above-average results and negative values indicating below-average results. In this example, athletes with *Z*-scores on the negative axis (displayed in red) performed below the meta-analysis estimate, while those on the positive axis (displayed in green) performed above the meta-analysis result. Both STEN and *Z*-scores are commonly used in sports settings to interpret and contextualise performance data [51].

## Discussion

The primary aim of this systematic review and meta-analysis was to establish normative data for the physical qualities of women’s football players. The secondary aim was to investigate the effect of performance classification (i.e. participant tier) on these physical qualities using a modified version of the Participant Classification Framework [17]. By synthesising data from 288 studies and 982 unique groups, these findings provide an evidence-based foundation for understanding seven physical capacities (cardiorespiratory fitness, acceleration, sprint, change of direction, maximum velocity, lower limb strength, and lower limb power) within women’s football. These findings reveal substantial differences in physical performance across participant tiers, with clear improvements in intermittent aerobic capacity, sprint performance and lower limb power as players progress from sub-elite to elite levels. Field-based assessments, particularly the YYIRL1, were more effective at differentiating cardiorespiratory fitness between participant tiers versus laboratory-based measures such as $\dot{V}O_2$ max. These findings provide evidence-based benchmarks that can be used to provide insights for athlete profiling and development, talent identification and training program design in women’s football.

Providing benchmarks for physical qualities enables women’s football practitioners to assess athlete’s physical capacities relative to established norms, allowing for more objective evaluations and facilitating comparisons between athletes at different stages of their careers [52, 53]. Previous work in this area employed generalised additive models to develop age-specific reference intervals for physical performance in youth male football players on the basis of longitudinal data from a single population [52]. The meta-analytic approach used in this study allows for the aggregation of data from a wide range of studies, enhancing the generalisability of the findings. However, variability in testing protocols, study designs and the classification of performance levels across the included studies introduced heterogeneity, which may affect the precision of the results. Despite these challenges, the results of this meta-analysis provide robust, data-driven insights to benchmark key physical qualities that can guide practice globally.

The results of this study revealed distinct trends in the cardiorespiratory fitness of athletes across participant tiers. While the effect of participant tier on $\dot{V}O_2$ max was inconclusive when comparing Tier 3 with Tiers 4 and 5, the data suggest that YYIRL1 and V_IFT_ performance substantially improves with increasing tier levels. Specifically, the moderate-to-large effects observed when advancing from Tier 2 to higher tiers for YYIRL1 and V_IFT_ indicate that athletes at higher competitive levels exhibit superior intermittent aerobic capacity. These results align with existing research that highlights the increasing physicality and intensity of women’s football, particularly at the elite level (Tier 4 and 5) [10, 54]. Studies have shown that elite female athletes cover greater distances at high speeds and engage in more repeated sprints during matches compared with players at lower levels [55–57]. In addition, the large differences in field-based cardiorespiratory fitness assessments echo prior research in women’s football that emphasises the role of intermittent fitness in distinguishing between performance levels [58, 59], talent development [53] and physical match performances [58]. These differences may stem from training exposure, higher match demands and professionalisation, with elite players benefitting from superior physical preparation, sport science integration and structured conditioning programs.

In contrast, laboratory-based outcome measures of $\dot{V}O_2$ max presented smaller, inconclusive differences between tiers, perhaps attributed to differences in test protocols and spirometry data processing techniques across studies, emphasising the need for standardised testing procedures in future research (for example [60]). Notwithstanding this heterogeneity, an interesting outcome of the meta-analysis was the notable difference between measures of maximal aerobic speed when determined directly in a laboratory setting (*v* $\dot{V}$O_2_ max: 14.6 [90% CIs 13.6 to 15.6 km h^−1^]) or via proxy measures in the field (set distance/time trials: 12.7 [90% CIs 10.9 to 14.5 km·h^−1^]). While expected, considering the longer duration and differing nature (incremental versus fixed intensity) of these assessments, the magnitude of this difference has implications for exercise intensity prescription based on the construct of maximal aerobic speed and warrants consideration when selecting cardiorespiratory fitness assessments. It is noteworthy that our analysis could not compare across participant tiers for several cardiorespiratory fitness tests (YYIR2, *v* $\dot{V}$O_2_ max, set distance/time trial, and VAM-EVAL), highlighting the need for normative data, particularly in lower tier participants (e.g. Tier 2). Overall, the findings suggests that intermittent fitness assessments may be more sensitive to distinguishing between competitive levels than traditional $\dot{V}$O_2_ max assessments, likely due to their sport-specific features and test familiarity. However, the composite nature of intermittent assessments means that they also assess factors such as muscular endurance, power, and recovery capacity, which may obscure inferences solely related to cardiorespiratory fitness [61].

The findings of this study highlight important nuances in how anaerobic performance metrics differentiate athletes across competitive tiers, particularly in acceleration and sprint time, COD and maximal velocity. While acceleration over short distances (5 m and 10 m) showed inconclusive results, sprint performance over longer distances (20 m and 30 m) demonstrated clearer differentiations between participant tiers. Specifically, moderate-to-large effects were observed as athletes progressed through higher tiers, particularly between Tiers 2 and Tiers 4 and 5. Maximal velocity followed a similar trend, with higher-tier athletes generally displaying greater top-end speeds, though the effect size was smaller compared with sprint performance over shorter distances. This suggests that while maximal velocity is an important differentiator of participant tier, it may not be as sensitive as sprint performance. The consistency of these findings across both sprint distances (i.e. 20 and 30 m) highlights the robustness of sprint measures, albeit clearer differences were observed in 30 m compared with 20 m trials. Notably, the inconclusive results for acceleration performance may stem from higher measurement error in shorter acceleration distances (e.g. 5 m), which could be attributed to factors such as test procedures (e.g. starting position) or technology (e.g. photocells type, height) limitations [45]. This could subsequently reduce the signal-to-noise ratio and obscure true differences when comparing athletes across tiers, particularly when relying on a single assessment. The 5–0–5 test was the only COD measure that could be compared across tiers. Similar to acceleration time, it showed no clear differences between Tier 2 to Tier 3, suggesting that COD may be less sensitive to participant tier. However, we were unable to compare Tier 4 and 5 athletes owing to a lack of data among these levels, making it uncertain whether similar trends would hold at higher tiers. Overall, sprint performance and maximum velocity emerged as the most reliable differentiators of performance level, emphasising the critical importance of sprinting speed as a key performance indicator in women’s football. While our results show inconclusive evidence of acceleration differentiating between participant tiers, acceleration performance is a crucial physical quality for successful football performance [11].

The analysis of lower limb performance, encompassing both strength and power metrics, reveals distinct trends across competitive tiers. Lower limb strength, measured by 1RM back squat performance, showed inconclusive effects between Tier 3 and Tiers 4 and 5. In contrast, lower limb power, particularly assessed using SJ and CMJ, demonstrated clearer differences across tiers. While the progression from Tier 2 to Tier 3 showed inconclusive effects, athletes in Tiers 4 and 5 generally exhibited substantial improvements in jump height, especially for SJ and CMJ with unrestricted arm movement. This indicates that power-based assessments may be more sensitive than maximal strength tests in differentiating between competitive levels. This finding may reflect the inability of less experienced athletes (i.e. Tier 2) to effectively use maximal strength during dynamic lower limb power assessments, perhaps due to lower technical proficiency or neuromuscular efficiency compared with more experienced (i.e. Tier 4–5) athletes. Similar trends have been observed in men’s football, where elite players had a moderately greater CMJ height versus sub-elite [62]. However, some jump assessments, such as BJ, showed less consistent results (albeit only comparing Tier 3 with Tiers 4 and 5), potentially due to variations in testing protocols, technique and/or test familiarity. In addition, there were clear differences in performance results when the same protocols were assessed using optical or contact devices versus force plates. Optical or contact devices typically overestimated jump performance by 1.3 to 4.6 cm compared with force plates, with higher discrepancies observed in CMJ and unrestricted arm movement. Therefore, practitioners should be aware of the discrepancies that can arise when using different technologies and even when deriving jump height from different metrics. Overall, while lower limb strength may plateau across competitive tiers (at least when reaching Tier 3), lower limb power remains a key differentiator, particularly when measured with standardised, reproducible protocols.

The findings from this meta-analysis have several practical applications. First, the normative data can serve as a benchmarking tool for women’s football practitioners, allowing them to assess the physical status or development of their athletes at various competitive levels. The substantial differences in intermittent cardiorespiratory fitness, sprint and lower limb power across tiers suggest that these qualities should be prioritised in training programs aimed at progressing athletes to higher levels of competition. These data also have direct implications for talent identification, as practitioners can use the physical profiles of higher-tier players to set performance benchmarks for emerging talent. Finally, the meta-regression results, accessible through the web-based dashboard, provide an interactive platform for practitioners to compare their athletes across the broader cohorts of women’s football, as well as against tier-specific benchmarks [50]. This tool enhances the practical utility of our findings, making them readily applicable in day-to-day training environments.

Future research is warranted to further our understanding of physical qualities in women’s football by expanding data collation beyond non-peer-reviewed sources and to incorporate other important factors, such as the evolving professionalism, position-specific demands, female health, and development status. Although the competitive nature of elite football is perceived a barrier to data sharing, valuable information about physical qualities is collected routinely by practitioners globally; collating this information with appropriate ethical safeguarding and data-quality guidelines would provide a more comprehensive and reproducible benchmarking data-set. An ongoing synthesis of peer-reviewed, grey-literature and practitioner/player-donated physical quality data would also capture future evolution in the women’s game as the investment and resourcing grows. This may be particularly relevant considering the global differences in training and playing opportunities afforded to developing female players [63]. The influence of positional roles on physical performance was not explicitly examined in this meta-analysis, although it is well established that different positions require distinct physical attributes [10]. The inclusion of position as a modifying factor was not feasible, as only a small number of studies reported data at the positional level. Furthermore, inconsistencies in how positional groups were defined (e.g. variations in categorisation across studies) further restricted our ability to investigate positional group effects. In addition, rigorous studies exploring the impact of the systemic hormonal effects and female health factors on physical performance could provide insights into optimising athletic preparation and readiness for female players. Moreover, future studies could explore the longitudinal development of physical qualities in female footballers (e.g. Datson et al. [53]), tracking their progression across different stages of their career. The sex data gap is accentuated in the realm of talent development [64], and the development of physical qualities is likely to have a different trajectory owing to differences in physiology and biological maturation [65].

The risk of bias assessment identified areas where improved transparency would enhance the interpretation and applicability of findings. While not a primary aim, our analysis highlighted reporting gaps that can be addressed to facilitate clearer physical benchmarks in future studies. Participant demographics and performance classification can be improved by consistent reporting of mean age, competition details (i.e. country, league name, and division) and position-specific data. These details would refine athlete classification using the Modified Participant Framework and allow for more meaningful comparisons, given the positional differences in match demands [10, 66]. Providing contextual details, such as location timing (i.e. year/season), and stage of season are particularly warranted in women’s football, where the increasing professionalism likely influences physical qualities over time. Measurement accuracy and protocol details were also frequently underreported. Specifying test conditions (i.e. whether arm movement was restricted in CMJ assessments) is critical to interpret and compare findings. Improved reporting practices will strengthen the comparability, applicability and the systematic organisation of data, ensuring that physical performance data are applicable for player monitoring and development.

Despite the strengths of our study, several limitations must be acknowledged. First, the heterogeneity across studies in terms of testing protocols, sample sizes and competition levels introduced variability that may have influenced our results. Although we accounted for these differences using multiple strategies, such as adopting universally recognised categories for test protocols and technologies, employing multi-level meta-analysis to model heterogeneity at multiple levels, and performing meta-regression to evaluate participant tier differences, the variation in protocols, particularly for tests such as $\dot{V}$O_2_ max and CMJ, may still affect the generalisability of the findings. In addition, the classification of athletes into participant tiers relied on a modified version of the Participant Classification Framework [17]. While this framework was developed in consultation with experts and incorporated several rounds, some subjectivity was involved in assigning tiers, especially for studies that lacked detailed information about the competitive level of the participants. Furthermore, the data available for Tier 5 athletes, representing senior national teams that reach the quarterfinals and later stages of international competitions, remained limited and constrained the depth of analysis at the highest performance level. This meta-analysis focused primarily on seven physical capacities and a range of testing protocols that while deemed relevant in a practical setting at the time of this research being conducted, in the future may be redundant if more gender-specific protocols are utilised. While the physical capacities selected in this research are critical for football performance, other factors such as technical skills, decision-making ability, chronological and training age, and psychological resilience also play crucial roles in athletes’ success but were not examined in this study. Another limitation to acknowledge is that the inclusion of research in English only introduces publication bias. Lastly, the increasing professionalism of the women’s game must be acknowledged as a limitation in the context of the data synthesised in this study. Although we excluded studies prior to 2003, the increased density and intensity denoted in matches over the last decade [54] would likely be accompanied by physical quality development. However, given the potential lag in publishing data and the lack of available detail on assessment scheduling in manuscripts, we were unable to explore the impact of professionalisation.

## Conclusions

This systematic review and meta-analysis provide a comprehensive overview of the physical performance qualities of women’s football athletes across varying levels of competition. By synthesising data from a wide array of studies, this research has established normative benchmarks that highlight the critical role of sprinting ability, lower limb power and intermittent cardiorespiratory fitness in differentiating players across the performance scale. Field-based tests, particularly those assessing intermittent fitness, demonstrated the greatest difference in performance between each of the participant tiers. This may suggest that these pragmatic tests are effective at capturing the physical performance of women’s football players. These findings offer valuable insights for football practitioners (coaches, sport scientists and medical staff), enabling more tailored and evidence-based approaches to athlete development, talent identification, and training design. Although there are inherent limitations due to the variability in testing protocols and classification systems, the study underscores the importance of standardising performance assessments in women’s football to foster a more globally consistent development pathway.

## Supplementary Information

Below is the link to the electronic supplementary material.

Supplementary file1 (DOCX 31 KB)

Supplementary file2 (DOCX 17 KB)

Supplementary file3 (DOCX 109 KB)

Supplementary file4 (DOCX 24 KB)

Supplementary file5 (PDF 761 KB)

Supplementary file6 (DOCX 28 KB)

Supplementary file7 (DOCX 74923 KB)

Supplementary file8 (DOCX 31 KB)

Supplementary file9 (DOCX 28 KB)

Supplementary file10 (PDF 400 KB)

## Declarations

### Availability of Data and Material

Supplementary materials for this paper are also available on the Open Science Framework at https://osf.io/d8twf/.

### Code Availability

The authors have provided a code sample and output for one protocol (20-m sprint) available in Supplementary Material 5. In addition, all relevant codes related to data processing, figures, tables and statistical analyses are available at https://osf.io/d8twf/.
