# Correction: Benchmarking the Physical Performance Qualities in Women’s Football: A Systematic Review and Meta-analysis Across the Performance Scale

**DOI:** 10.1007/s40279-025-02337-9

**Published:** 2025-10-29

**Authors:** Heidi R. Compton, Ric Lovell, Dawn Scott, Jo Clubb, Tzlil Shushan

**Affiliations:** 1 School of Biomedical Sciences and Pharmacy, University of Newcastle, Callaghan, Australia; 2 Applied Sport Science and Exercise Testing Laboratory, University of Newcastle, Ourimbah, Australia; 3 FIFA, Women’s Development Programme, Women’s Football Division, Zurich, Switzerland; 4 Faculty of Science, Medicine and Health, University of Wollongong, Wollongong, Australia; 5 Global Performance Insights Ltd, London, UK

**Correction: Sports Med (2025)** 10.1007/s40279-025-02251-0

The following errors were identified after publication:

- In the Graphical Abstract, Modified Participant Classification Framework section, Tier 2 was listed as ‘Groups training > 3x/wk…’; this should have read ‘ ≤ 3x/wk’.
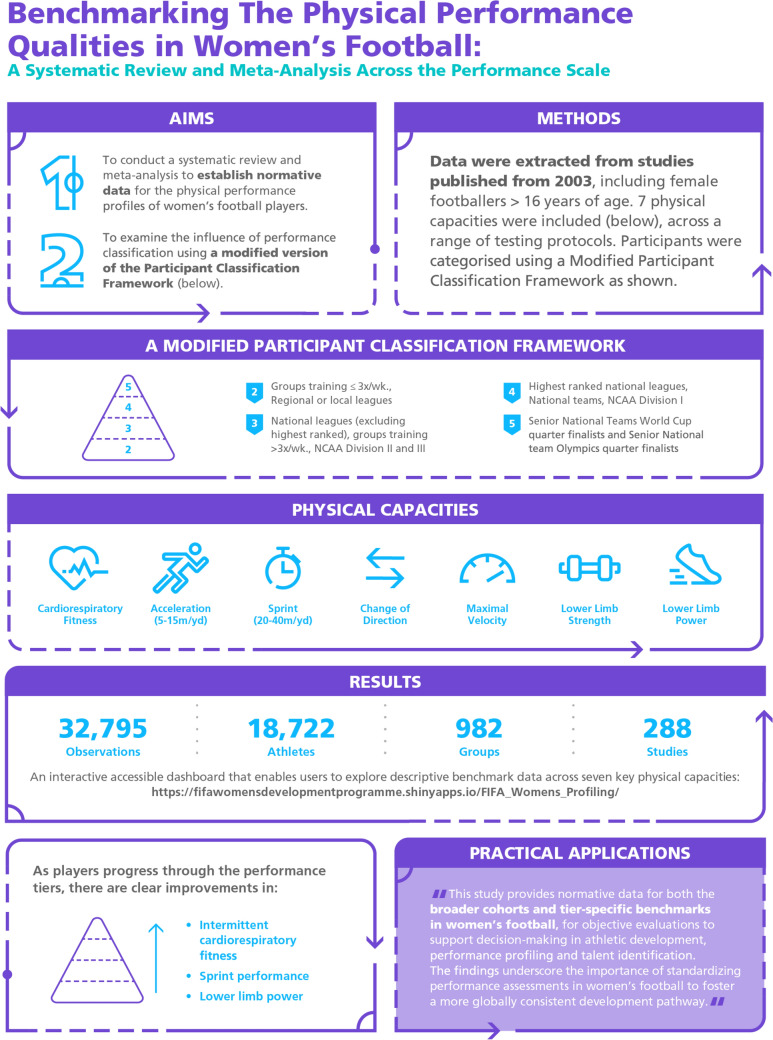
- The caption for Fig. 1 referred to the inclusion of 287 studies in the systematic review and meta-analysis; the correct number was 288.
- Section 3.3.2 Acceleration Time referred to ‘Comparisons across Tier 2 and Tier 3 (*b* = 0.02 cm [90% CIs − 0.11 to 0.14) and Tier 2 and Tiers 4 and 5 (*b* =  − 0.01 cm [90% CIs –0.14 to 0.13)’. The correct unit for this measurement was 's' (seconds) not 'cm'.

The original article has been corrected.

