# Supplementary material for: Benchmarking the Physical Performance Qualities in Women’s Football: A Systematic Review and Meta-analysis Across the Performance Scale
Source: Sports Med. 2025 Sep 1;56(Suppl 1):127–55. doi: 10.1007/s40279-025-02251-0 (PMC13314896; doi:10.1007/s40279-025-02251-0)
Supplement: Supplementary file 2 — Supplementary file2 (DOCX 17 KB) [file 40279_2025_2251_MOESM2_ESM.docx]

**Title:** Benchmarking The Physical Performance Qualities in Women’s Football: A Systematic Review and Meta-Analysis Across the Performance Scale

**Authors:**

Heidi R. Compton^1,2,3^ - 0000-0002-5818-4450

Ric Lovell^3,4^ - 0000-0001-5859-0267

Dawn Scott^3^ - 0009-0000-6763-1235

Jo Clubb^3,5^ - 0000-0002-6509-7531

Tzlil Shushan^3,4^ - 0000-0002-0544-1986

**Affiliations:**

^1^ School of Biomedical Sciences and Pharmacy, University of Newcastle, Australia;

^2^ Applied Sport Science and Exercise Testing Laboratory, University of Newcastle, Ourimbah, Australia;

^3^ FIFA, Women’s Development Programme, Women’s Football Division, Zurich, Switzerland;

^4^ Faculty of Science, Medicine and Health, University of Wollongong, Australia;

^5^ Global Performance Insights Ltd, London, United Kingdom

**Corresponding author:**

Heidi Compton

[Heidi.compton@newcastle.edu.au](mailto:Heidi.thornton@newcastle.edu.au)

University of Newcastle

Callaghan, Australia

**Table S2.** Searching strategy syntaxes.

| **Pubmed (search 1)** |
| --- |
| ((Football OR soccer OR "football player*" OR "soccer player* "OR"football athlete*" OR "soccer athlete*") AND (Female OR woman OR women OR lady OR ladies)) AND (Performance OR physical OR endurance OR aerobic OR cardio* OR anaerobic OR fitness OR stamina OR capacity OR sprint* OR agility OR speed OR strength OR power OR force OR jump* OR plyo*) Filters: Full text, English, from 2003 - 2023 |
| **Pubmed (updated search)** |
| ((Football OR soccer OR "football player*" OR "soccer player* "OR"football athlete*" OR "soccer athlete*") AND (Female OR woman OR women OR lady OR ladies)) AND (Performance OR physical OR endurance OR aerobic OR cardio* OR anaerobic OR fitness OR stamina OR capacity OR sprint* OR agility OR speed OR strength OR power OR force OR jump* OR plyo*) Filters: Full text, English, from 2023 - 2024 Sort by: Most Recent |
| **SportDiscuss (search 1)** |
| (Football OR soccer OR “football player*” OR “soccer player*” OR “football athlete*” OR “soccer athlete*” ) AND ( Female OR woman OR women OR lady OR ladies ) AND ( Performance OR physical OR endurance OR aerobic OR cardio* OR anaerobic OR fitness OR stamina OR capacity OR sprint* OR agility OR speed OR strength OR power OR force OR jump* OR plyo* )  **Limiters** - Publication Date: 20030422-; Language: English; Publication Type: Academic Journal  **Expanders** - Apply related words  **Search modes** - Proximity |
| **SportDiscuss (updated search)** |
| (Football OR soccer OR “football player*” OR “soccer player*” OR “football athlete*” OR “soccer athlete*”) AND (Female OR woman OR women OR lady OR ladies) AND (Performance OR physical OR endurance OR aerobic OR cardio* OR anaerobic OR fitness OR stamina OR capacity OR sprint* OR agility OR speed OR strength OR power OR force OR jump* OR plyo*)  **Limiters** - Peer Reviewed; Published Date: 20230422-20240531; References Available; Publication Type: Academic Journal; Document Type: Article  **Expanders** - Apply equivalent subjects  **Search modes** - Proximity |
| **Scopus (search 1)** |
| ( TITLE-ABS-KEY ( football  OR  soccer  OR  "football player*"  OR  "soccer player*"  OR  "football athlete*"  OR  "soccer athlete*" )  AND  TITLE-ABS-KEY ( female  OR  woman  OR  women  OR  lady  OR  ladies )  AND  TITLE-ABS-KEY ( performance  OR  physical  OR  endurance  OR  aerobic  OR  cardio*  OR  anaerobic  OR  fitness  OR  stamina  OR  capacity  OR  sprint*  OR  agility  OR  speed  OR  strength  OR  power  OR  force  OR  jump*  OR  plyo* ) )  AND  PUBYEAR  >  2002  AND  ( LIMIT-TO ( DOCTYPE ,  "ar" ) )  AND  ( LIMIT-TO ( LANGUAGE ,  "English" ) )  AND  ( LIMIT-TO ( PUBSTAGE ,  "final" ) ) |
| **Scopus (updated search)** |
| ( TITLE-ABS-KEY ( ( football OR soccer OR "football player*" OR "soccer player*" OR "football athlete*" OR "soccer athlete*" ) ) AND TITLE-ABS-KEY ( ( female OR woman OR women OR lady OR ladies ) ) AND TITLE-ABS-KEY ( ( performance OR physical OR endurance OR aerobic OR cardio* OR anaerobic OR fitness OR stamina OR capacity OR sprint* OR agility OR speed OR strength OR power OR force OR jump* OR plyo* ) ) ) AND PUBYEAR > 2022 AND PUBYEAR < 2024 AND ( LIMIT-TO ( DOCTYPE , "ar" ) ) AND ( LIMIT-TO ( LANGUAGE , "English" ) ) AND ( LIMIT-TO ( EXACTKEYWORD , "Female" ) ) AND ( LIMIT-TO ( SRCTYPE , "j" ) ) |
| **Web of Science (search 1)** |
| Football OR soccer OR “football player*” OR “soccer player*” OR “football athlete*” OR “soccer athlete*” (All Fields) and Female OR woman OR women OR lady OR ladies (All Fields) and Performance OR physical OR endurance OR aerobic OR cardio* OR anaerobic OR fitness OR stamina OR capacity OR sprint* OR agility OR speed OR strength OR power OR force OR jump* OR plyo* (All Fields) and Article (Document Types) and English (Languages) |
| **Web of Science (updated search)** |
| Football OR soccer OR “football player*” OR “soccer player*” OR “football athlete*” OR “soccer athlete*” (All Fields) and Female OR woman OR women OR lady OR ladies (All Fields) and Performance OR physical OR endurance OR aerobic OR cardio* OR anaerobic OR fitness OR stamina OR capacity OR sprint* OR agility OR speed OR strength OR power OR force OR jump* OR plyo* (All Fields) and Article (Document Types) and English (Languages) |
