# Supplementary material for: Benchmarking the Physical Performance Qualities in Women’s Football: A Systematic Review and Meta-analysis Across the Performance Scale
Source: Sports Med. 2025 Sep 1;56(Suppl 1):127–55. doi: 10.1007/s40279-025-02251-0 (PMC13314896; doi:10.1007/s40279-025-02251-0)
Supplement: Supplementary file 3 — Supplementary file3 (DOCX 109 KB) [file 40279_2025_2251_MOESM3_ESM.docx]

**Title:** Benchmarking The Physical Performance Qualities in Women’s Football: A Systematic Review and Meta-Analysis Across the Performance Scale

**Authors:**

Heidi R. Compton^1,2,3^ - 0000-0002-5818-4450

Ric Lovell^3,4^ - 0000-0001-5859-0267

Dawn Scott^3^ - 0009-0000-6763-1235

Jo Clubb^3,5^ - 0000-0002-6509-7531

Tzlil Shushan^3,4^ - 0000-0002-0544-1986

**Affiliations:**

^1^ School of Biomedical Sciences and Pharmacy, University of Newcastle, Australia;

^2^ Applied Sport Science and Exercise Testing Laboratory, University of Newcastle, Ourimbah, Australia;

^3^ FIFA, Women’s Development Programme, Women’s Football Division, Zurich, Switzerland;

^4^ Faculty of Science, Medicine and Health, University of Wollongong, Australia;

^5^ Global Performance Insights Ltd, London, United Kingdom

**Corresponding author:**

Heidi Compton

Heidi.compton@newcastle.edu.au

University of Newcastle

Callaghan, Australia

| **Table S3.** Summary of all study characteristics. | | | | | | | | | | | | | | |
| --- | --- | --- | --- | --- | --- | --- | --- | --- | --- | --- | --- | --- | --- | --- |
| **Covidence** | **Reference** | **Lead Author and Year of Publication** | **Sample Size** | **Country** | **Tier** | **Playing Position** | **Physical Performance Quality** | | | | | | | |
|  |  |  |  |  |  |  | **CRF** | **Acc** | **Sprint** | **CoD** | **LL Power** | **LL Strength** | **Max velocity** | |
| #10012 | [67] | Barrera, J., 2022 | 52 | Chile | Tier 3 | Outfield + GK | ✓ |  |  |  |  |  |  | |
| #4272 | [68] | Alexiou, H., 2008 | 15 | UK | Tier 4 |  | ✓ |  |  |  |  |  |  | |
| #18920 | [69] | Alimoradi, M., 2023 | 60 | Iran | Tier 2 |  |  |  |  | ✓ |  |  |  | |
| #13767 | [70] | Almonroeder, T., 2020 | 10 | USA | Tier 4 |  |  |  |  |  | ✓ |  |  | |
| #18921 | [71] | Aloy, A., 2024 | 84 | Spain | Tier 3 |  |  |  |  |  | ✓ |  |  | |
| #4378 | [72] | Amundsen, R., 2022 | 320 | Norway | Tier 3 |  |  | ✓ | ✓ |  | ✓ |  |  | |
| #4387 | [12] | Andersen, E., 2018 | 102 | USA | Tier 3 |  |  | ✓ | ✓ | ✓ | ✓ | ✓ |  | |
| #4422 | [73] | Andersson, H., 2008 | 51 | Sweden and Norway | Tier 3 | Outfield | ✓ |  | ✓ |  | ✓ |  |  | |
| #4491 | [74] | Arazi, H., 2017 | 32 | Iran | Tier 2 |  | ✓ |  |  |  |  |  |  | |
| #4507 | [75] | Archiza, B., 2018 | 36 | Brazil | Tier 3 |  | ✓ |  |  |  |  |  |  | |
| #4503 | [76] | Archiza, B., 2020 | 16 | Brazil | Tier 3 | Outfield | ✓ |  |  |  |  |  |  | |
| #4522 | [77] | Armada-Cortés, E., 2020 | 32 | Spain | Tier 3 |  |  |  |  |  | ✓ |  |  | |
| #4524 | [78] | Armada-Cortés, E., 2022 | 24 | Spain | Tier 3 |  |  |  |  |  | ✓ |  |  | |
| #4527 | [79] | Armada-Cortés, E., 2022 | 19 | Spain | Tier 3 |  |  |  |  |  | ✓ |  |  | |
| #4587 | [80] | Asgari, M., 2023 | 128 | Iran | Tier 2 |  |  | ✓ | ✓ | ✓ | ✓ |  |  | |
| #200001 | [81] | Aspin, G., 2024 | 91 | UK | Tier 2 |  | ✓ |  | ✓ |  |  |  | ✓ | |
| #4778 | [82] | Baptista, I., 2022 | 108 | Norway | Tier 3 | Outfield |  |  |  |  |  |  | ✓ | |
| #4237 | [83] | Bartolini, A., 2011 | 36 | USA | Tier 4 |  |  | ✓ | ✓ |  |  |  |  | |
| #4912 | [84] | Baumgart, C., 2014 | 14 | Germany | Tier 4 |  | ✓ |  |  |  |  |  |  | |
| #4907 | [85] | Baumgart, C., 2018 | 70 | Germany | Tier 4 |  |  | ✓ | ✓ |  |  |  | ✓ | |
| #200007 | [86] | Belamjahad, A., 2024 | 384 | Morocco | Tier 3 | Outfield | ✓ | ✓ | ✓ | ✓ | ✓ | ✓ |  | |
| #5034 | [87] | Bendiksen, M., 2013 | 34 | Norway | Tier 3 |  | ✓ |  |  |  |  |  |  | |
| #5181 | [88] | Bishop, C., 2019 | 48 | UK | Tier 3 |  |  | ✓ | ✓ | ✓ |  |  |  | |
| #5308 | [89] | Bonilla, A., 2020 | 14 | Spain | Tier 3 |  |  |  |  |  | ✓ |  |  | |
| #5320 | [90] | Booysen, M., 2021 | 179 | South Africa | Tier 3 | Outfield + GK | ✓ | ✓ | ✓ |  | ✓ |  |  | |
| #5384 | [91] | Bozzini, B., 2020 | 11 | USA | Tier 4 | Outfield | ✓ |  |  |  |  |  |  | |
| #5380 | [92] | Bozzini, B., 2021 | 120 | USA | Tier 4 |  | ✓ |  |  |  | ✓ |  |  | |
| #5391 | [93] | Bradley, P., 2014 | 38 | N/A | Tier 2 |  | ✓ |  |  |  |  |  |  | |
| #5516 | [94] | Broodryk, A., 2020 | 43 | Africa | Tier 2 |  | ✓ |  |  |  |  |  |  | |
| #5703 | [95] | Busko, K., 2020 | 26 | NA | Tier 2 |  |  |  |  |  | ✓ |  |  | |
| #5781 | [96] | Çakir, E., 2019 | 90 | N/A | Tier 2 |  |  |  | ✓ | ✓ |  |  |  | |
| #5807 | [97] | Campa, F., 2022 | 160 | Italy | Tier 3 |  |  |  | ✓ |  | ✓ |  |  | |
| #5820 | [98] | Campo, S., 2009 | 80 | Spain | Tier 4 | Outfield + GK |  |  |  |  | ✓ |  |  | |
| #5825 | [99] | Can, F., 2004 | 34 | Turkey | Tier 3 |  |  |  |  |  | ✓ |  |  | |
| #200002 | [100] | Can, I., 2019 | 66 | Turkey | Tier 3 |  | ✓ | ✓ | ✓ | ✓ | ✓ |  |  | |
| #18959 | [101] | Carvalho, G., 2024 | 70 | Brazil | Tier 3 |  | ✓ | ✓ | ✓ |  | ✓ |  |  | |
| #5951 | [102] | Castagna, C., 2013 | 82 | Italy | Tier 4 | Outfield |  |  |  |  | ✓ |  |  | |
| #5956 | [103] | Castagna, C., 2020 | 25 | N/A | Tier 4 | Outfield | ✓ |  |  |  |  |  |  | |
| #18275 | [104] | Chen, T., 2023 | 65 | Taiwan | Tier 3 |  | ✓ |  | ✓ | ✓ | ✓ |  |  | |
| #6214 | [105] | Chou, T., 2021 | 12 | China | Tier 3 |  | ✓ |  |  |  |  |  |  | |
| #6238 | [106] | Christensen, B., 2020 | 72 | USA | Tier 4 | Outfield + GK |  | ✓ | ✓ |  | ✓ |  |  | |
| #6289 | [107] | Clark, I., 2013 | 32 | USA | Tier 3 |  | ✓ |  |  |  |  |  |  | |
| #6305 | [108] | Clark, M., 2003 | 56 | USA | Tier 4 |  | ✓ |  |  |  | ✓ |  |  | |
| #6571 | [109] | Costa, J., 2022 | 32 | Portugal | Tier 3 | Outfield | ✓ |  |  |  |  |  |  | |
| #6575 | [110] | Costa, J., 2022 | 17 | Portugal | Tier 3 | Outfield | ✓ |  |  |  |  |  |  | |
| #6628 | [111] | Čović, N., 2016 | 51 | N/A | Tier 3 |  | ✓ |  |  |  |  |  |  | |
| #6727 | [112] | Curtis, R., 2021 | 139 | US | Tier 4 |  | ✓ |  |  |  |  |  |  | |
| #18297 | [113] | da Silva, V., 2024 | 126 | Portugal | Tier 3 |  |  |  |  |  | ✓ | ✓ |  | |
| #6844 | [114] | Daria, D., 2022 | 60 | Indonesia | Tier 2 | Outfield |  |  | ✓ | ✓ |  |  |  | |
| #6870 | [115] | Datson, N., 2022 | 315 | UK | Tier 3, Tier 4 | Outfield + GK | ✓ | ✓ | ✓ |  | ✓ |  |  | |
| #5860 | [116] | de Araújo, M., 2020 | 174 | Germany | Tier 4 | Outfield |  | ✓ | ✓ |  | ✓ |  |  | |
| #6924 | [117] | De Bruin, M., 2021 | 34 | South Africa | Tier 2 |  |  |  | ✓ |  | ✓ |  |  | |
| #18313 | [118] | De Oliveira, F., 2023 | 105 | Brazil | Tier 3 |  | ✓ | ✓ |  | ✓ | ✓ |  |  | |
| #6964 | [119] | de Oliveira, M., 2021 | 18 | USA | Tier 4 | Outfield | ✓ |  |  |  |  |  |  | |
| #7078 | [120] | Delextrat, A., 2018 | 126 | N/A | Tier 2 | Outfield | ✓ |  | ✓ |  | ✓ |  |  | |
| #7112 | [121] | Dent, J., 2015 | 8 | N/A | Tier 3 |  | ✓ |  |  |  |  |  |  | |
| #7131 | [122] | Devismes, M., 2021 | 63 | Belgium | Tier 3, Tier 2 |  |  |  |  |  |  |  | ✓ | |
| #18328 | [123] | Díaz-Ochoa, A., 2023 | 210 | Mexico | Tier 2 | Outfield | ✓ |  | ✓ |  | ✓ | ✓ |  | |
| #7194 | [124] | Diaz-Seradilla, E., 2022 | 17 | Spain | Tier 4 | Outfield |  |  |  |  |  |  | ✓ | |
| #7229 | [125] | Dillern, T., 2012 | 64 | Norway | Tier 2 | Outfield + GK | ✓ |  |  |  |  |  |  | |
| #7308 | [126] | Dolci, F., 2021 | 34 | Australia | Tier 2 | Outfield + GK | ✓ |  |  |  |  |  |  | |
| #7353 | [127] | Dos'Santos, T., 2018 | 30 | UK | Tier 2 |  |  | ✓ |  | ✓ |  |  |  | |
| #7359 | [128] | Douchet, T., 2021 | 38 | France | Tier 4 | Outfield |  |  |  |  | ✓ |  |  | |
| #7375 | [129] | Doyle, B., 2020 | 100 | Ireland | Tier 4 |  | ✓ | ✓ | ✓ |  |  |  |  | |
| #7376 | [130] | Doyle, B., 2021 | 275 | Ireland | Tier 4 | Outfield + GK | ✓ | ✓ | ✓ |  | ✓ |  |  | |
| #7621 | [131] | Emmonds, S., 2019 | 60 | England | Tier 4 |  |  | ✓ | ✓ | ✓ | ✓ |  |  | |
| #7707 | [132] | Esco, M., 2014 | 30 | USA | Tier 3 |  | ✓ |  |  |  |  |  |  | |
| #7712 | [133] | Esco, M., 2014 | 40 | USA | Tier 3 |  | ✓ |  |  |  |  |  |  | |
| #7703 | [134] | Esco, M., 2016 | 18 | USA | Tier 4 |  | ✓ |  |  |  |  |  |  | |
| #7783 | [135] | Falatic, J., 2015 | 34 | USA | Tier 4 |  | ✓ |  |  |  |  |  |  | |
| #7879 | [136] | Favero, T., 2016 | 32 | N/A | Tier 4 |  | ✓ |  |  |  |  |  |  | |
| #7914 | [137] | Fernandes, R., 2022 | 10 | Portugal | Tier 3 | Outfield |  |  |  |  |  |  | ✓ | |
| #8038 | [138] | Fischerova, P., 2021 | 136 | Poland | Tier 3 | Outfield + GK |  |  |  |  | ✓ |  |  | |
| #8041 | [139] | Fischetti, F., 2019 | 56 | Italy | Tier 3 |  |  |  |  |  | ✓ |  |  | |
| #8065 | [140] | Flatt, A., 2017 | 8 | USA | Tier 4 |  | ✓ |  |  |  |  |  |  | |
| #19007 | [141] | Fu, H., 2023 | 17 | N/A | Tier 3 |  |  |  | ✓ |  |  |  |  | |
| #8282 | [142] | Gabrys, T., 2019 | 46 | Poland | Tier 4 |  | ✓ |  |  |  |  |  |  | |
| #8322 | [143] | Gao, F., 2023 | 120 | China | NA |  |  |  | ✓ |  | ✓ |  |  | |
| #8592 | [144] | Goncalves, L., 2021 | 330 | Portugal | Tier 3 | Outfield + GK | ✓ | ✓ | ✓ |  | ✓ |  |  | |
| #8589 | [145] | Gonçalves, L., 2021 | 440 | Portugal | Tier 3 | Outfield + GK | ✓ | ✓ | ✓ |  | ✓ |  | ✓ | |
| #8595 | [146] | Gonçalves, L., 2021 | 286 | Portugal | Tier 3 |  | ✓ | ✓ | ✓ |  | ✓ |  |  | |
| #8606 | [147] | Gonzalez-Fernandez, F., 2022 | 32 | Spain | Tier 2 |  |  |  | ✓ |  | ✓ |  |  | |
| #8609 | [148] | González-Fernández, F., 2022 | 108 | Spain | Tier 3 | Outfield |  |  | ✓ |  | ✓ |  | ✓ | |
| #19020 | [149] | González-Fernández, F., 2024 | 64 | Spain | Tier 3 |  | ✓ |  |  |  | ✓ |  |  | |
| #8614 | [150] | Gonzalez-Garcia, J., 2019 | 72 | Spain | Tier 2, Tier 3 |  | ✓ | ✓ |  | ✓ |  |  |  | |
| #8647 | [151] | Goranovic, K., 2021 | 60 | Serbia | Tier 3 | Outfield |  |  |  |  | ✓ |  |  | |
| #8667 | [152] | Goulart, K. N., 2020 | 100 | Brazil | Tier 3 |  | ✓ | ✓ | ✓ |  | ✓ |  |  | |
| #8671 | [153] | Goulart, K., 2020 | 90 | Brazil | Tier 3 | Outfield |  | ✓ | ✓ |  | ✓ | ✓ |  | |
| #8709 | [154] | Gravina, L., 2017 | 21 | N/A | Tier 3 |  | ✓ |  | ✓ |  | ✓ |  |  | |
| #8729 | [155] | Green, M., 2013 | 39 | USA | Tier 4 |  | ✓ |  |  |  |  |  |  | |
| #8765 | [156] | Grieco, C., 2012 | 22 | USA | Tier 4 |  | ✓ |  |  |  |  |  |  | |
| #8883 | [157] | Guthrie, B., 2021 | 322 | USA | Tier 3 |  |  |  |  |  | ✓ | ✓ |  | |
| #8970 | [158] | Hamilton, A., 2008 | 20 | USA | Tier 4 |  |  |  |  |  | ✓ |  |  | |
| #8984 | [159] | Hammami, M., 2020 | 144 | Tunisia | Tier 4 |  | ✓ | ✓ | ✓ |  | ✓ |  |  | |
| #18414 | [160] | Hammami, M., 2023 | 336 | Tunisia | Tier 3 |  | ✓ | ✓ | ✓ | ✓ | ✓ |  |  | |
| #9198 | [161] | Haugen, T., 2012 | 165 | Norway | Tier 5, Tier 3 |  |  |  |  | ✓ |  |  |  | |
| #9181 | [162] | Haugen, T., 2014 | 66 | Norway | Tier 3 |  | ✓ |  |  |  | ✓ |  |  | |
| #9194 | [163] | Haugen, T., 2014 | 370 | Norway | Tier 5, Tier 3, Tier 4 |  | ✓ |  |  |  |  |  |  | |
| #9189 | [164] | Haugen, T., 2020 | 1043 | Norway | Tier 4, Tier 3, Tier 5 |  | ✓ | ✓ |  |  |  | ✓ |  | |
| #9193 | [165] | Haugen, T., 2021 | 95 | Norway | Tier 4 |  |  |  |  |  | ✓ |  |  | |
| #9358 | [166] | Hewitt, A., 2014 | 30 | Australia | Tier 5 | Outfield |  |  | ✓ |  | ✓ |  |  | |
| #9413 | [167] | Hirose, N., 2015 | 80 | Japan | Tier 3 |  |  | ✓ |  |  |  |  |  | |
| #9704 | [168] | Idrizovic, K., 2014 | 140 | Montenegro | Tier 4 | Outfield + GK | ✓ | ✓ | ✓ |  | ✓ |  |  | |
| #18449 | [169] | Igonin, P., 2024 | 165 | France | Tier 3 |  |  | ✓ | ✓ |  | ✓ |  |  | |
| #9751 | [170] | Ingebrigtsen, J., 2011 | 29 | Norway | Tier 3 | Outfield | ✓ |  |  |  |  |  |  | |
| #9764 | [171] | Inoue, Y., 2017 | 22 | Japan | Tier 2 |  | ✓ |  | ✓ |  |  |  |  | |
| #4176 | [172] | Ishida, A., 2021 | 12 | USA | Tier 4 |  |  |  |  |  | ✓ |  |  | |
| #9775 | [173] | Ishida, A., 2021 | 36 | USA | Tier 4 | Outfield |  |  |  |  | ✓ |  |  | |
| #9882 | [174] | Jakobsson, S., 2020 | 10 | US | Tier 2 |  |  |  |  | ✓ |  |  |  | |
| #9979 | [175] | Jeras, N., 2020 | 32 | Germany | Tier 3 |  |  |  |  |  | ✓ |  |  | |
| #9997 | [176] | Jimenez-Reyes, P., 2019 | 78 | Spain | Tier 4, Tier 3 |  | ✓ | ✓ |  |  |  |  |  | |
| #10019 | [177] | Johnson, A., 2013 | 64 | USA | Tier 2 |  |  |  | ✓ |  |  |  |  | |
| #10059 | [178] | Jones, M., 2010 | 56 | USA | Tier 3 |  |  |  |  |  |  | ✓ |  | |
| #19050 | [179] | Juillard, E., 2024 | 72 | France | Tier 3 | Outfield |  |  |  | ✓ |  |  |  | |
| #10129 | [180] | Julian, A., 2016 | 72 | Germany | Tier 3 | Outfield + GK |  | ✓ | ✓ |  | ✓ |  |  | |
| #10188 | [181] | Kammoun, M., 2020 | 225 | Tunisia | Tier 3 | Outfield + GK | ✓ |  | ✓ |  | ✓ |  |  | |
| #10609 | [182] | Kobal, R., 2021 | 96 | Brazil | Tier 4 | Outfield | ✓ |  |  |  | ✓ |  |  | |
| #10615 | [183] | Kobal, R., 2021 | 34 | Brazil | Tier 4 |  |  |  |  |  | ✓ |  |  | |
| #18501 | [184] | Köklü, Y., 2024 | 16 | Turkey | Tier 2 |  | ✓ |  |  |  |  |  |  | |
| #10753 | [185] | Krawczyk, M., 2021 | 12 | NA | Tier 3 |  |  |  |  |  | ✓ |  |  | |
| #10777 | [186] | Križaj, J., 2020 | 36 | Slovenia | Tier 3 | Outfield |  |  | ✓ |  | ✓ |  |  | |
| #10816 | [59] | Krustrup, P., 2005 | 14 | Denmark | Tier 3 | Outfield | ✓ |  |  |  |  |  |  | |
| #10828 | [187] | Krustrup, P., 2010 | 46 | Denmark | Tier 3 | Outfield | ✓ |  |  |  | ✓ |  |  | |
| #10901 | [188] | Kutlu, M., 2017 | 136 | N/A | Tier 2 |  |  |  | ✓ | ✓ |  |  |  | |
| #10913 | [189] | La Torre, A., 2007 | 117 | NA | Tier 2 | Outfield + GK |  |  |  |  | ✓ |  |  | |
| #18518 | [190] | Lee, Y., 2024 | 152 | South Korea | Tier 2 |  |  | ✓ | ✓ |  |  |  |  | |
| #11218 | [191] | Lewandowski, A., 2019 | 24 | Poland | Tier 3 |  |  |  |  |  | ✓ |  |  | |
| #11403 | [192] | Lockie, R. G., 2020 | 114 | USA | Tier 4 |  | ✓ | ✓ | ✓ | ✓ |  | ✓ |  | |
| #11398 | [193] | Lockie, R., 2017 | 60 | USA | Tier 4 |  |  | ✓ | ✓ |  |  |  |  | |
| #11399 | [194] | Lockie, R., 2017 | 105 | USA | Tier 4 | Outfield | ✓ | ✓ | ✓ |  |  |  |  | |
| #11393 | [195] | Lockie, R., 2018 | 171 | USA | Tier 4, Tier 3 | Outfield |  | ✓ |  | ✓ | ✓ |  |  | |
| #11407 | [196] | Lockie, R., 2018 | 156 | USA | Tier 4 | Outfield + GK | ✓ | ✓ | ✓ |  | ✓ |  |  | |
| #11475 | [197] | Lopez-Fernandez, J., 2018 | 144 | Spain | Tier 2 | Outfield |  |  |  |  | ✓ |  |  | |
| #11515 | [198] | Loturco, I., 2018 | 116 | Brazil | Tier 4 |  |  |  |  |  | ✓ |  |  | |
| #11510 | [199] | Loturco, I., 2019 | 32 | Brazil | Tier 4 |  |  |  |  |  | ✓ |  |  | |
| #11622 | [200] | Lyle, M., 2015 | 14 | NA | Tier 2 |  |  |  |  |  | ✓ |  |  | |
| #11637 | [201] | Lyons, M., 2021 | 20 | Australia | Tier 3, Tier 2 | Outfield | ✓ |  |  |  |  |  |  | |
| #18549 | [202] | Magal, M., 2023 | 84 | USA | Tier 3 | Outfield | ✓ |  | ✓ |  |  |  |  | |
| #11738 | [203] | Magrini, M., 2018 | 18 | USA | Tier 4 |  |  |  |  |  | ✓ |  |  | |
| #13814 | [204] | Mainer Pardos, E., 2019 | 230 | Spain | Tier 3 |  |  | ✓ | ✓ |  | ✓ |  |  | |
| #11750 | [205] | Mainer-Pardos, E., 2021 | 115 | Spain | Tier 3 |  |  | ✓ | ✓ |  |  |  | ✓ | |
| #11867 | [206] | Manson, S., 2014 | 33 | New Zealand | Tier 4 |  | ✓ |  |  |  |  |  |  | |
| #11872 | [207] | Manson, S., 2021 | 234 | Australia | Tier 5 | Outfield |  | ✓ | ✓ |  | ✓ |  | ✓ | |
| #11889 | [208] | Mara, J., 2015 | 272 | Australia | Tier 3 | Outfield | ✓ | 8 | ✓ |  |  |  |  | |
| #12006 | [209] | Martinez-Lagunas, V., 2014 | 54 | Germany | Tier 3 |  | ✓ |  |  |  |  |  |  | |
| #18563 | [210] | Martín-Moya, R., 2023 | 42 | Spain | Tier 2 |  | ✓ | ✓ |  | ✓ |  |  |  | |
| #12174 | [211] | McCormack, W., 2014 | 20 | USA | Tier 4 | Outfield | ✓ |  |  |  |  |  | ✓ | |
| #12189 | [212] | McCurdy, K., 2010 | 45 | USA | Tier 4 |  |  | ✓ |  |  | ✓ |  |  | |
| #12205 | [213] | McFadden, B., 2020 | 126 | USA | Tier 4 | Outfield + GK | ✓ |  |  |  | ✓ |  |  | |
| #12211 | [214] | McFadden, B., 2020 | 32 | USA | Tier 4 |  | ✓ |  |  |  | ✓ |  |  | |
| #12207 | [215] | McFadden, B., 2022 | 125 | USA | Tier 4 |  | ✓ |  |  |  | ✓ |  |  | |
| #12203 | [216] | McFadden, B., 2023 | 204 | USA | Tier 4 |  | ✓ |  |  |  | ✓ |  |  | |
| #12217 | [217] | McFarland, I., 2016 | 64 | USA | Tier 3 |  |  | ✓ | ✓ |  | ✓ |  |  | |
| #12220 | [218] | McGawley, K., 2006 | 9 | Australia | Tier 3 |  | ✓ |  |  |  |  |  |  | |
| #12225 | [219] | McGawley, K., 2015 | 8 | Australia | Tier 3 |  | ✓ |  |  |  |  |  |  | |
| #12413 | [220] | Merino-Muñoz, P., 2021 | 36 | Chile | Tier 3, Tier 4 |  | ✓ | ✓ |  | ✓ |  |  |  | |
| #12454 | [221] | Meylan, C., 2017 | 60 | Australia | Tier 4 |  |  | ✓ | ✓ |  |  |  | ✓ | |
| #12523 | [222] | Milanovic, Z., 2012 | 22 | Serbia | Tier 4 | Outfield + GK | ✓ |  |  |  |  |  |  | |
| #12525 | [223] | Milanovic, Z., 2012 | 88 | Serbia | Tier 4 | Outfield + GK | ✓ |  |  |  | ✓ |  |  | |
| #12565 | [224] | Miller, T., 2007 | 104 | USA | Tier 4 |  | ✓ |  |  |  |  |  |  | |
| #19106 | [225] | Moen, F., 2023 | 21 | Norway | Tier 3 |  |  |  |  |  |  |  | ✓ | |
| #12677 | [226] | Mohr, M., 2022 | 30 | N/A | Tier 3 | Outfield |  |  | ✓ |  |  |  |  | |
| #12759 | [227] | Montgomery, M., | 69 | USA | Tier 4 |  |  |  |  | ✓ |  |  |  | |
| #12789 | [228] | Morales, J., 2019 | 64 | Spain | Tier 4 |  | ✓ |  |  |  |  |  |  | |
| #200005 | [58] | Mujika, I., 2009 | 102 | Spain | Tier 4, Tier 3 | Outfield | ✓ |  |  |  | ✓ |  |  | |
| #13053 | [57] | Myhill, N., 2022 | 282 | UK | Tier 4, Tier 3 | Outfield + GK | ✓ |  |  |  |  |  | ✓ | |
| #13148 | [229] | Nayiroglu, S., 2022 | 288 | Turkey | Tier 2 | Outfield | ✓ | ✓ | ✓ | ✓ | ✓ |  |  | |
| #13155 | [230] | Nealer, A., 2017 | 96 | USA | Tier 4, Tier 2 |  | ✓ | ✓ |  |  |  |  |  |  |
| #13159 | [231] | Nebil, G., 2014 | 98 | Tunisia | Tier 3 |  | ✓ | ✓ | ✓ |  |  |  |  | |
| #13170 | [232] | Nedrehagen, E., 2015 | 30 | N/A | Tier 3 | Outfield | ✓ |  |  |  |  |  |  | |
| #13214 | [233] | Nesser, T., 2009 | 48 | USA | Tier 4 |  |  |  | ✓ |  | ✓ | ✓ |  | |
| #13355 | [234] | Nonnato, A., 2022 | 192 | UK | Tier 3 | Outfield |  | ✓ | ✓ | ✓ | ✓ |  |  | |
| #13494 | [235] | Oberacker, L., 2012 | 114 | USA | Tier 3 | Outfield + GK |  | ✓ | ✓ |  | ✓ |  |  | |
| #18631 | [236] | Oliveira, R., 2023 | 140 | Portugal | Tier 3 | Outfield | ✓ |  | ✓ | ✓ | ✓ |  |  | |
| #19127 | [237] | Oliveira, R., 2023 | 108 | Brazil | Tier 4 | Outfield | ✓ |  |  |  | ✓ |  |  | |
| #13694 | [238] | Ozbar, N., 2014 | 108 | Turkey | Tier 3 | Outfield |  |  | ✓ |  | ✓ |  |  | |
| #13692 | [239] | Ozbar, N., 2015 | 200 | Turkey | Tier 3 | Outfield |  | ✓ | ✓ |  | ✓ |  |  | |
| #13719 | [240] | Pacholek, M., 2020 | 156 | Slovakia | Tier 4 |  |  |  |  |  | ✓ | ✓ |  | |
| #13744 | [241] | Painter, K., 2022 | 24 | USA | Tier 4 |  |  |  |  |  | ✓ |  |  | |
| #19134 | [242] | Papaevangelou, E., 2023 | 114 | N/A | Tier 3 |  | ✓ |  |  |  |  |  |  | |
| #200004 | [243] | Paravlic, A., 2024 | 105 | Slovenia | Tier 4 |  |  |  |  |  | ✓ |  | ✓ | |
| #13817 | [244] | Pardos-Mainer, E, 2022 | 72 | Spain | Tier 3 |  |  | ✓ | ✓ |  |  |  |  | |
| #13820 | [245] | Pardos-Mainer, E., 2020 | 228 | Spain | Tier 3 |  |  | ✓ | ✓ |  | ✓ |  |  | |
| #13836 | [246] | Park, L., 2019 | 54 | USA | Tier 5 | Outfield | ✓ |  |  |  |  |  | ✓ | |
| #13864 | [247] | Parpa, K., 2022 | 19 | N/A | Tier 3 | Outfield | ✓ |  |  |  |  |  |  | |
| #13933 | [248] | Paulsen, K., 2023 | 72 | N/A | Tier 3 |  | ✓ |  |  |  |  |  |  | |
| #13962 | [249] | Peart, A., 2018 | 180 | USA | Tier 3 |  | ✓ |  |  |  | ✓ |  |  | |
| #13966 | [250] | Pecci, J., 2023 | 80 | Spain | Tier 2, Tier 3 | Outfield + GK |  |  | ✓ |  | ✓ |  |  | |
| #13987 | [251] | Pedersen, S., 2019 | 460 | Norway | Tier 2 |  |  | ✓ |  |  | ✓ | ✓ |  | |
| #13990 | [252] | Pedersen, S., 2021 | 72 | Norway | Tier 2 |  |  | ✓ |  |  | ✓ |  |  | |
| #13993 | [253] | Pedersen, S., 2021 | 222 | Norway | Tier 2 | Outfield |  | ✓ |  |  | ✓ | ✓ | ✓ | |
| #19142 | [254] | Perrotta, A., 2023 | 40 | NA | Tier 3 |  |  |  |  |  | ✓ |  |  | |
| #19140 | [255] | Perrotta, A., 2024 | 216 | NA | Tier 3 |  | ✓ | ✓ |  |  | ✓ |  |  | |
| #14108 | [256] | PerutȚ-Florin, T., 2021 | 42 | Romania | Tier 3 |  |  |  |  |  | ✓ |  |  | |
| #14230 | [257] | Poehling, R., 2021 | 1008 | NA | Tier 4, Tier 5 | Outfield | ✓ | ✓ | ✓ |  | ✓ |  | ✓ | |
| #14281 | [258] | Polman, R., 2004 | 324 | UK | Tier 2 | Outfield + GK |  |  | ✓ |  | ✓ |  |  | |
| #18673 | [259] | Pompeo, A., 2024 | 42 | Portugal | Tier 4 |  |  |  |  |  | ✓ |  |  | |
| #14319 | [260] | Porrati-Paladino, G., 2021 | 30 | Spain | Tier 2 |  |  |  |  |  | ✓ |  |  | |
| #14348 | [261] | Pournemati, P., 2009 | 12 | N/A | NA |  | ✓ |  |  |  |  |  |  | |
| #14386 | [262] | Preissler, A., 2023 | 33 | Brazil | Tier 3 | Outfield |  |  | ✓ |  |  |  |  | |
| #14439 | [263] | Prudholme, D., 2022 | 18 | USA | Tier 4 | Outfield |  |  |  |  |  |  | ✓ | |
| #14460 | [264] | Purdom, T., 2020 | 55 | USA | Tier 4 |  | ✓ |  |  |  |  |  |  | |
| #14471 | [265] | Putnam, A., 2012 | 30 | USA | Tier 2 |  |  |  | ✓ |  |  |  |  | |
| #14505 | [266] | Queiróz, A., 2013 | 25 | Brazil | Tier 2 |  | ✓ |  |  |  |  |  |  | |
| #18691 | [267] | Raeder, C., 2024 | 72 | Germany | Tier 4 | Outfield |  | ✓ |  |  |  |  |  | |
| #200006 | [268] | Rajkumar, S., 2015 | 50 | India | Tier 3, Tier 2 |  |  |  |  | ✓ |  |  |  |  |
| #14585 | [269] | Ramirez-Campillo, R., 2016 | 180 | Chile | Tier 2 | Outfield + GK |  |  | ✓ |  | ✓ |  |  | |
| #14595 | [270] | Ramirez-Campillo, R., 2016 | 304 | NA | Tier 3 | Outfield + GK |  |  | ✓ | ✓ | ✓ |  |  | |
| #14582 | [271] | Ramirez-Campillo, R., 2018 | 138 | Chile | Tier 2 | Outfield + GK | ✓ | ✓ |  |  | ✓ |  |  | |
| #14599 | [272] | Ramos, G., 2021 | 555 | Brazil | Tier 4 |  | ✓ |  |  |  | ✓ |  |  | |
| #19156 | [273] | Reyes-Laredo, F., 2024 | 216 | N/A | Tier 2 |  |  |  | ✓ |  | ✓ |  |  | |
| #14818 | [274] | Rhea, M., 2009 | 40 | N/A | Tier 3 |  | ✓ |  |  |  |  |  |  | |
| #14833 | [275] | Ribeiro, R., 2020 | 96 | Brazil | Tier 4 |  | ✓ |  | ✓ |  |  |  |  | |
| #14896 | [276] | Risso, F., 2017 | 132 | USA | Tier 4 | Outfield | ✓ | ✓ | ✓ |  | ✓ |  |  | |
| #14965 | [277] | Rodriguez, C., 2018 | 60 | Mexico | Tier 3 |  | ✓ |  |  |  | ✓ |  |  | |
| #200000 | [278] | Rodríguez, L., 2024 | 25 | Spain | Tier 2 |  | ✓ |  |  |  |  |  |  | |
| #15056 | [279] | Rosas, F., 2017 | 150 | NA | Tier 2 |  |  |  | ✓ |  | ✓ |  |  | |
| #18732 | [280] | Roso-Moliner, A., 2023 | 190 | Spain | Tier 3 |  |  | ✓ | ✓ | ✓ |  |  |  | |
| #18734 | [281] | Roso-Moliner, A., 2023 | 532 | Spain | Tier 3 |  |  | ✓ | ✓ | ✓ | ✓ |  |  | |
| #15110 | [282] | Rowan, A., 2012 | 88 | USA | Tier 2, Tier 3 |  | ✓ |  |  |  |  |  |  |  |
| #15170 | [283] | Ruscello, B., 2023 | 19 | Italy | Tier 3 |  | ✓ |  |  |  |  |  |  | |
| #15293 | [284] | Sanchez, M., 2022 | 84 | N/A | Tier 3 | Outfield + GK | ✓ |  | ✓ |  | ✓ |  |  | |
| #15292 | [285] | Sánchez, M., 2022 | 72 | Spain | Tier 2 |  |  |  | ✓ |  | ✓ |  |  | |
| #15324 | [286] | Sanders, G., 2017 | 10 | USA | Tier 4 |  | ✓ |  |  |  |  |  |  | |
| #18752 | [287] | Savolainen, E., 2023 | 75 | Finland | Tier 3 | Outfield | ✓ |  | ✓ |  | ✓ |  |  | |
| #15426 | [288] | Sayers, A., 2008 | 80 | NA | Tier 3 |  |  | ✓ | ✓ |  |  |  |  | |
| #15523 | [289] | Schons, P., 2023 | 308 | Brazil | Tier 3 |  | ✓ | ✓ | ✓ |  | ✓ |  |  | |
| #15574 | [290] | Scott, D., 2018 | 154 | USA | Tier 5 |  | ✓ | ✓ | ✓ |  |  |  | ✓ | |
| #15571 | [10] | Scott, D., 2020 | 1540 | USA | Tier 4, Tier 3 | Outfield + GK | ✓ | ✓ | ✓ |  | ✓ |  | ✓ | |
| #15578 | [291] | Scott, D., 2020 | 72 | USA | Tier 4 | Outfield | ✓ |  |  |  |  |  | ✓ | |
| #18757 | [292] | Scott, D., 2024 | 80 | USA | Tier 5 | Outfield | ✓ | ✓ | ✓ |  |  |  |  | |
| #15591 | [293] | Sedan, S., 2009 | 190 | Spain | Tier 2, Tier 4 | Outfield + GK |  |  |  |  | ✓ |  |  | |
| #15674 | [294] | Shalfawi, S., 2013 | 160 | Norway | Tier 3 |  |  |  | ✓ |  | ✓ |  |  | |
| #15679 | [295] | Shalfawi, S., 2013 | 136 | Norway | Tier 3 |  | ✓ |  | ✓ |  | ✓ |  |  | |
| #15678 | [296] | Shalfawi, S., 2014 | 120 | Norway | Tier 3 |  |  |  | ✓ |  | ✓ |  |  | |
| #15776 | [297] | Siegler, J., 2003 | 68 | USA | Tier 2 |  |  |  |  |  | ✓ |  |  | |
| #18781 | [298] | Singha, P., 2023 | 120 | India | Tier 2 |  | ✓ |  |  |  |  |  |  | |
| #15908 | [299] | Sjökvist, J., 2011 | 112 | USA | Tier 4 |  | ✓ |  | ✓ |  | ✓ |  |  | |
| #19190 | [300] | Snyder, B., 2024 | 20 | USA | Tier 4 |  |  |  |  |  | ✓ |  |  | |
| #16020 | [301] | Somboonwong, J., 2015 | 26 | Thailand | Tier 3 |  | ✓ |  | ✓ |  |  |  |  | |
| #16074 | [302] | Souglis, A., 2023 | 96 | Greece | Tier 3 | Outfield | ✓ |  |  |  |  |  |  | |
| #16081 | [303] | Souza, F., 2018 | 12 | Brazil | Tier 2 |  | ✓ |  |  |  |  |  |  | |
| #16119 | [304] | Sporiš, G., 2011 | 96 | Croatia | Tier 3 | Outfield + GK | ✓ |  |  |  |  | ✓ |  | |
| #16155 | [305] | Stanković, M., 2022 | 360 | Serbia | Tier 3 |  | ✓ | ✓ | ✓ | ✓ | ✓ |  |  | |
| #200003 | [306] | Stanković, M., 2023 | 120 | Serbia | Tier 3 | Outfield |  |  | ✓ | ✓ | ✓ |  |  | |
| #16154 | [307] | Stankoviƒá, M., 2022 | 96 | Serbia | Tier 3 |  |  | ✓ | ✓ |  | ✓ |  |  | |
| #16185 | [308] | Steffen, K., 2008 | 62 | Norway | Tier 3 |  |  |  | ✓ |  | ✓ |  |  | |
| #16223 | [309] | Stepinski, M., 2020 | 216 | Poland | Tier 4 | Outfield |  | ✓ | ✓ |  | ✓ |  |  | |
| #16226 | [310] | Stevens, T., 2016 | 16 | Netherlands | Tier 3 | Outfield | ✓ |  |  |  |  |  |  | |
| #16237 | [311] | Stieg, J., 2011 | 85 | USA | Tier 4 |  |  |  |  |  | ✓ |  |  | |
| #16308 | [312] | Struzik, A., 2019 | 14 | Poland | Tier 3 |  |  |  |  |  | ✓ |  |  | |
| #16327 | [313] | Suchomel, T. J., 2015 | 22 | USA | Tier 4 |  |  |  |  |  | ✓ |  |  | |
| #16330 | [314] | Suchomel, T., 2016 | 24 | USA | Tier 4 |  |  |  |  |  | ✓ |  |  | |
| #18811 | [315] | Sun, D., 2023 | 36 | China | Tier 3 |  |  |  |  |  | ✓ | ✓ |  | |
| #19197 | [18] | Sydney, M., 2024 | 15 | Australia | Tier 2 | Outfield | ✓ |  |  |  |  |  |  | |
| #16431 | [316] | Szulc, A., 2017 | 45 | Poland | Tier 2, Tier 3 |  |  |  |  | ✓ |  |  |  |  |
| #16643 | [317] | Thomas, C., 2021 | 11 | UK | Tier 2 | Outfield |  |  |  |  | ✓ |  |  | |
| #16648 | [318] | Thomas, G., 2007 | 14 | USA | Tier 4 |  |  |  |  |  |  | ✓ |  | |
| #16714 | [319] | Timothy, M., 2023 | 44 | Ghana | Tier 2 |  |  |  |  | ✓ |  |  |  | |
| #16757 | [320] | Toro-Roman, V., 2023 | 96 | Spain | Tier 3 | Outfield + GK | ✓ |  |  |  | ✓ |  |  | |
| #18833 | [321] | Toro-Román, V., 2023 | 24 | Spain | Tier 3 |  | ✓ |  |  |  |  |  |  | |
| #16782 | [322] | Tounsi, M., 2018 | 66 | Tunisia | Tier 3 |  | ✓ |  |  |  |  |  |  | |
| #16802 | [323] | Trapp, J., 2021 | 80 | USA | Tier 4 | Outfield | ✓ |  |  |  |  |  | ✓ | |
| #18836 | [324] | Tseng, W., 2024 | 96 | Taiwan | Tier 3 |  | ✓ |  | ✓ |  | ✓ |  |  | |
| #16938 | [325] | Unveren, A., 2015 | 140 | NA | Tier 3 |  |  | ✓ | ✓ | ✓ |  |  |  | |
| #16944 | [326] | Upton, D., 2011 | 216 | USA | Tier 4 |  |  | ✓ | ✓ |  |  |  |  | |
| #18844 | [327] | Vagle, M., 2023 | 428 | Norway | Tier 3 | Outfield + GK |  |  | ✓ |  | ✓ |  |  | |
| #17210 | [328] | Vescovi, J., 2006 | 256 | USA | Tier 4 | Outfield + GK |  | ✓ | ✓ |  | ✓ |  |  | |
| #17195 | [329] | Vescovi, J., 2008 | 51 | USA | Tier 4 |  |  |  |  |  | ✓ |  |  | |
| #17238 | [330] | Vescovi, J., 2011 | 113 | USA | Tier 4 |  |  |  |  |  | ✓ |  |  | |
| #17191 | [23] | Vescovi, J., 2012 | 100 | USA | Tier 3 | Outfield |  |  |  |  |  |  | ✓ | |
| #17233 | [331] | Vescovi, J., 2021 | 580 | USA | Tier 3 |  |  | ✓ | ✓ |  |  |  | ✓ | |
| #17280 | [332] | Villaseca-Vicun ̃a, R., 2022 | 190 | Chile | Tier 4 | Outfield + GK | ✓ | ✓ | ✓ |  | ✓ | ✓ |  | |
| #17275 | [333] | Villaseca-Vicuna, R., 2021 | 300 | Chile | Tier 4 | Outfield + GK | ✓ | ✓ | ✓ | ✓ | ✓ | ✓ |  | |
| #17277 | [334] | Villaseca-Vicuna, R., 2021 | 182 | Chile | Tier 4 | Outfield | ✓ | ✓ | ✓ | ✓ | ✓ | ✓ | ✓ | |
| #17274 | [335] | Villaseca-Vicuña, R., 2021 | 275 | Chile | Tier 4 | Outfield | ✓ | ✓ | ✓ |  | ✓ | ✓ |  | |
| #19212 | [336] | Villaseca-Vicuna, R., 2024 | 34 | Chile | Tier 4 |  |  |  |  |  |  |  | ✓ | |
| #17361 | [337] | Wagganer, J., 2014 | 24 | USA | Tier 3 |  |  |  | ✓ |  |  |  |  | |
| #17394 | [338] | Wallace, B., 2008 | 33 | USA | Tier 3 |  |  |  |  |  | ✓ | ✓ |  | |
| #17401 | [339] | Wallmann, H., 2008 | 12 | US | Tier 4 |  |  |  |  | ✓ |  |  |  | |
| #17476 | [340] | Watson, A., 2017 | 6 | USA | Tier 4 |  | ✓ |  |  |  |  |  |  | |
| #18876 | [341] | Winther, A., 2024 | 100 | Norway | Tier 3 | Outfield |  |  |  |  |  |  | ✓ | |
| #18886 | [342] | Xing, Y., 2023 | 50 | China | Tier 3 | Outfield | ✓ |  |  |  |  |  |  | |
| #18887 | [343] | Xing, Y., 2023 | 6 | China | Tier 3 |  | ✓ |  |  |  |  |  |  | |
| #17942 | [344] | You, S., 2023 | 42 | China | Tier 3 |  |  |  |  |  | ✓ |  |  | |
| #17982 | [345] | Zabaloy, S., 2022 | 182 | Chile | Tier 4 | Outfield | ✓ | ✓ | ✓ |  | ✓ | ✓ |  | |
| #18088 | [346] | Zhang, Q., 2021 | 92 | France | Tier 3 |  |  | ✓ | ✓ |  |  |  |  | |
| #18082 | [347] | Zhang, Q., 2022 | 200 | France | Tier 2 |  |  | ✓ |  | ✓ |  |  | ✓ | |

CRF: cardiorespiratory fitness, CoD: change of direction, LL: lower limb
