# Supplementary material for: Benchmarking the Physical Performance Qualities in Women’s Football: A Systematic Review and Meta-analysis Across the Performance Scale
Source: Sports Med. 2025 Sep 1;56(Suppl 1):127–55. doi: 10.1007/s40279-025-02251-0 (PMC13314896; doi:10.1007/s40279-025-02251-0)
Supplement: Supplementary file 4 — Supplementary file4 (DOCX 24 KB) [file 40279_2025_2251_MOESM4_ESM.docx]

**Title:** Benchmarking The Physical Performance Qualities in Women’s Football: A Systematic Review and Meta-Analysis Across the Performance Scale

**Authors:**

Heidi R. Compton^1,2,3^ - 0000-0002-5818-4450

Ric Lovell^3,4^ - 0000-0001-5859-0267

Dawn Scott^3^ - 0009-0000-6763-1235

Jo Clubb^3,5^ - 0000-0002-6509-7531

Tzlil Shushan^3,4^ - 0000-0002-0544-1986

**Affiliations:**

^1^ School of Biomedical Sciences and Pharmacy, University of Newcastle, Australia;

^2^ Applied Sport Science and Exercise Testing Laboratory, University of Newcastle, Ourimbah, Australia;

^3^ FIFA, Women’s Development Programme, Women’s Football Division, Zurich, Switzerland;

^4^ Faculty of Science, Medicine and Health, University of Wollongong, Australia;

^5^ Global Performance Insights Ltd, London, United Kingdom

**Corresponding author:**

Heidi Compton

[Heidi.compton@newcastle.edu.au](mailto:Heidi.thornton@newcastle.edu.au)

University of Newcastle

Callaghan, Australia

| Table S4. Study quality assessment characteristics modified using the Downs and Black [1] tool, and the STROBE (STrengthening the Reporting of OBservational studies in Epidemiology) checklists [2]. Studies were assessed and rated as; Yes (1), No (0), N/A (/) or Unable to determine (0) | | | | |
| --- | --- | --- | --- | --- |
| Item n. | **Scale Item n.** | **Item question** | **Descriptions and further details** | **Dimension** |
| 1 | B&D (1) / STROBE (3) | Is the hypothesis/aim/objective of the study clearly described? | Authors provide clear aims/objectives for the study in the abstract or introduction sections | Reporting |
| 2 | B&D (2) / STROBE (7) | Are the main outcomes to be measured clearly described in the Introduction or Methods section? | Authors provide adequate information regarding the all testing protocols, procedures, and technology used.  Example 1: For acceleration and sprint time protocols, authors provide details on the technology used (e.g., timing gates and brand), testing procedures (e.g., starting position, distance from the gate), and cueing instructions (e.g., self-start, countdown cue).  Example 2: For jump protocols, authors provide details on the technology used (e.g., force plate, jimp mat and brand), testing procedure (e.g., SJ versus CMJ, hands position), and any cueing instruction implemented. | Reporting |
| 3 | B&D (3) / STROBE (14) | Are the characteristics of the subjects included in the study clearly described? | Authors report relevant athlete characteristics, which were assessed against the inclusion criteria of the Population component in the PICO model. Participant tier classification are clearly identified using the modified participant framework (Table 2 in the paper). Reported details per group or subgroups include gender, mean age and category, country, and relevant information related to training schedules or competition participation. | Reporting |
| 4 | B&D (6) / STROBE (15) | Are the main findings of the study clearly described? | Authors clearly report descriptive data (mean and standard deviation) for all testing protocols used in our database for performance outcomes. | Reporting |
| 5 | B&D (11) | Were the subjects asked to participate in the study representative of the entire population from which they were recruited? | Authors clearly report whether the recruited subjects were representative of the population from which they were drawn. This includes details on whether the entire squad was recruited, whether positional groups were reported to ensure balanced representation, and any selection criteria or exclusions that may have influenced the sample composition (e.g., availability, medical status, multiple participant tiers). | External validity |
| 6 | B&D (20) / STROBE (8) | Were the main outcome measures used accurate (valid and reliable)? | Authors discuss the measurement properties (validity, reliability) of all selected testing protocols and measurement tools, typically within the Methods section. This is done either by presenting results within the study itself (e.g., test-retest reliability) or by referencing previous research that has validated similar procedures. | Internal validity |
| 7 | B&D (22) / STROBE (5) | Does the authors outline the setting, locations, and relevant dates, including periods of recruitment, exposure, follow-up, and data collection. | Authors provide adequate information regarding the study settings and environment, including factors that may impact performance outcomes (e.g., indoor vs. outdoor conditions, environmental data). Authors specify the season phase (e.g., preseason vs. inseason) and clearly outline the exposure timeframes (e.g., longitudinal observation or repeated measures design.  Example: For field-based cardiorespiratory protocols, authors provide details on testing locations (e.g., outdoor natural grass), relevant environmental conditions, and season phase. | Internal validity |
| 8 | B&D (26) / STROBE (12) | Were losses of subjects to follow‐up taken into account? | Authors report whether the originally recruited sample is accurately reflected in the final findings (e.g., single and repeated observations), including any dropouts or changes in sample size throughout the study. This ensures accurate data retention and allows for appropriate weighting of each effect in the analysis. | Internal validity |
| 9 | STROBE (22) | Funding and potential conflict of interest | Authors declare whether a funding source was involved in the research and report any potential conflicts of interest. This includes disclosure of financial support, sponsorships, or affiliations with technology providers, test protocol creators, or any other entities that may have influenced the testing protocol and/or performance outcomes | Other |

**References**

1. Downs SH, Black N. The feasibility of creating a checklist for the assessment of the methodological quality both of randomised and non-randomised studies of health care interventions. Journal of Epidemiology and Community Health. 1998;52(6):377-84.

2. Von Elm E, Altman DG, Egger M, Pocock SJ, Gøtzsche PC, Vandenbroucke JP. The Strengthening the Reporting of Observational Studies in Epidemiology (STROBE) statement: guidelines for reporting observational studies. The Lancet. 2007;370(9596):1453-7.
