# Supplementary material for: Benchmarking the Physical Performance Qualities in Women’s Football: A Systematic Review and Meta-analysis Across the Performance Scale
Source: Sports Med. 2025 Sep 1;56(Suppl 1):127–55. doi: 10.1007/s40279-025-02251-0 (PMC13314896; doi:10.1007/s40279-025-02251-0)
Supplement: Supplementary file 5 — Supplementary file5 (PDF 761 KB) [file 40279_2025_2251_MOESM5_ESM.pdf]

Title: Benchmarking The Physical Performance Qualities in Women's Football: A Systematic Review and Meta-Analysis Across the Performance Scale

Authors:

Heidi R. Compton[1,2,3] - 0000-0002-5818-4450

Ric Lovell[3,4] - 0000-0001-5859-0267

Dawn Scott[3] - 0009-0000-6763-1235

Jo Clubb[3,5] - 0000-0002-6509-7531

Tzlil Shushan[3,4] - 0000-0002-0544-1986

Affiliations:

[1] School of Biomedical Sciences and Pharmacy, University of Newcastle, Australia;

[2] Applied Sport Science and Exercise Testing Laboratory, University of Newcastle, Ourimbah, Australia;

[3] FIFA, Women's Development Programme, Women's Football Division, Zurich, Switzerland;

[4] Faculty of Science, Medicine and Health, University of Wollongong, Australia;

[5] Global Performance Insights Ltd, London, United Kingdom

Corresponding author:

Heidi Compton

Heidi.compton@newcastle.edu.au

University of Newcastle

Callaghan, Australia

Benchmarking Physical Performance Qualities in Women's Football: A Systematic Review and Meta-Analysis Across the Performance Scale

Tzlil Shushan

2024-09-18

The following overview presents the code used in this meta-analysis paper, focusing on an example of the 20 m sprint time meta-analysis. It outlines the key phases of the statistical processes and provides a guide to understanding the methodological approach

---

Load Libraries

```
knitr::opts_chunk$set(echo = T)
library(readxl)
library(tidyverse)
library(metafor)
library(clubSandwich)
library(orchaRd)
library(ggh4x)
library(ggthemes)
library(ggridges)
library(knitr)
library(plotly)
library(kableExtra)
```

Read Data

```
TS_data <- read_xlsx("/Users/tshushan/Dropbox/Research & Collaboration Projects/Women's Football Review FIFA/Physical Qualities Workflow/Tzlil_Physical Qualities Women's Football Data.xlsx", sheet = "Data Coding Sprint", skip = 1)

HT_data <- read_xlsx("/Users/tshushan/Dropbox/Research & Collaboration Projects/Women's Football Review FIFA/Physical Qualities Workflow/Heidi_Physical Qualities Womens Football Data.xlsx", sheet = "Data Coding Sprint", skip = 1)

TS_data <- TS_data %>%
  mutate(Group.id = `Group ID`) # rename group id

HT_data <- HT_data %>%
  mutate(Group.id = `Group ID` + max(TS_data$`Group ID`)) # rename group id and add to the max value in the first dataset

extended_data <- rbind(TS_data, HT_data)

colnames(extended_data) <- gsub(" ", ".", colnames(extended_data)) # rename columns using replace spaces with dots
```

Explore the data

First, we explore the overall dataset. The summary includes the number of distinct studies, the number of unique groups, and the total sample size (accounting for unique participants across groups). Additionally, we report the total number of individual observations.

```
data_summary <- data.frame(
  studies = nrow(table(extended_data$`Covidence.#`)), # overall number of studies
  groups = nrow(table(extended_data$Group.id)), # overall number of groups
  sample.size = extended_data %>%
  group_by(Group.id) %>%
  summarise(max_n = max(Sample.Size)) %>% # overall sample size
  summarise(sum(max_n)),
  n = sum(extended_data$Sample.Size)) # overall individual observations

kable(
  data_summary,
  caption = "Summary table of sprint performance",
  align = c("c","c","c","c","c","c"),
  col.names = c("No. Studies",
                "No. Groups",
                "Sample Size",
                "Overall Obs.")) %>%
  kable_styling(bootstrap_options = c("hover"), full_width = F)
```

Summary table of sprint performance

No. Studies	No. Groups	Sample Size	Overall Obs.

117	196	3334	7327

Then, we explore individual assessments included in the dataset. Within each assessment protocol, we explore the number of estimates, number of unique groups, and number of unique studies. Regarding sample size, the median, min, and max are presented, as well as the overall sample size.

```
test_summary <- extended_data %>%
  group_by(Test) %>% # unique tests
  summarise(
    es = n(), # number of data estimates
    groups = n_distinct(Group.id), # number of groups
    studies = n_distinct(`Covidence.#`), # number of studies
    median_n = median(Sample.Size, na.rm = T), # median sample size
    min_n = min(Sample.Size, na.rm = T), # min sample size
    max_n = max(Sample.Size, na.rm = T), # max sample size
    overall_n = sum(Sample.Size, na.rm = T)) %>% # overall individual observations
  mutate(Test = factor(Test, levels = c("20 m", "20 yards", "25 m", "25 yards", "30
m", "40 m", "40 yards"))) %>% # custom levels table
    arrange(Test) # rearrange rows

kable(
  test_summary,
  caption = "Summary table of sprint performance within test protocol",
  align = c("c","c","c","c","c","c"),
  col.names = c("Test Protocol",
                "No. Estimates",
                "No. Groups",
                "No. Studies",
                "Median Group n",
                "Min. Group n",
                "Max. Group n",
                "Overall n")) %>%
  column_spec(1, bold = T, background = "#f0f0f0") %>%
  kable_styling(bootstrap_options = c("hover"), full_width = F)
```

Summary table of sprint performance within test protocol

Test Protocol	No. Estimates	No. Groups	No. Studies	Median Group n	Min. Group n	Max. Group n	Overall n
**20 m**	158	107	59	12.5	6	116	2609
**20 yards**	7	6	3	17.0	8	21	110
**25 m**	16	7	3	12.0	8	17	202
**25 yards**	6	3	1	9.0	9	9	54

**30 m**	164	116	68	15.0	3	116	2915
**40 m**	55	39	24	15.0	8	94	1144
**40 yards**	24	15	8	12.0	8	21	293

After preliminary analysis and data exploration, we excluded data points measured using stopwatch due to their identifications as notable outliers. The final dataset after exclusion of these data points is presented below:

```
extended_data <- extended_data %>%
  filter(Technology != "Stopwatch" & Technology != "NA")

test_summary_meta <- extended_data %>%
  group_by(Test) %>% # unique tests
  summarise(
    es = n(), # number of data estimates
    groups = n_distinct(Group.id), # number of groups
    studies = n_distinct(`Covidence.#`), # number of studies
    median_n = median(Sample.Size, na.rm = T), # median sample size
    min_n = min(Sample.Size, na.rm = T), # min sample size
    max_n = max(Sample.Size, na.rm = T), # max sample size
    overall_n = sum(Sample.Size, na.rm = T)) %>% # overall individual observations
  mutate(Test = factor(Test, levels = c("20 m", "20 yards", "25 m", "25 yards", "30
m", "40 m", "40 yards"))) %>% # custom levels table
    arrange(Test) # rearrange rows



kable(
  test_summary_meta,
  caption = "Summary table of sprint performance within test protocol",
  align = c("c","c","c","c","c","c"),
  col.names = c("Test Protocol",
                "No. Estimates",
                "No. Groups",
                "No. Studies",
                "Median Group n",
                "Min. Group n",
                "Max. Group n",
                "Overall n")) %>%
  column_spec(1, bold = T, background = "#f0f0f0") %>%
  kable_styling(bootstrap_options = c("hover"), full_width = F)
```

Summary table of sprint performance within test protocol

Test Protocol	No. Estimates	No. Groups	No. Studies	Median Group n	Min. Group n	Max. Group n	Overall n
**20 m**	157	106	58	12.0	6	116	2589
**20 yards**	7	6	3	17.0	8	21	110
**25 m**	16	7	3	12.0	8	17	202
**25 yards**	6	3	1	9.0	9	9	54
**30 m**	154	110	64	15.5	3	116	2790
**40 m**	54	38	23	15.5	8	94	1133
**40 yards**	13	9	4	9.0	8	21	162

20 m sprint meta-analysis

The first analysis in our meta focuses on 20 m sprint. To conduct this analysis, we begin by filtering the dataset to include only studies that measure sprint

```
data <- extended_data %>%
  filter(Test == "20 m") %>% # subset for test
  mutate(Final.Outcome = as.numeric(Final.Outcome), # rewrite final outcome
         Final.SD = as.numeric(Final.SD)) # rewrite final sd
```

To explore the distribution of data points (means) and identify potential outliers, we visualise a rain cloud plot with a histogram

```
ggplot(data, aes(x = Final.Outcome)) +
  geom_density(aes(y = ..density.. * max(..count..) / max(..density..)/20),
```

```
             color = "#08519c",
             fill = "#deebf7",
             alpha = 0.2,
             size = 0.6) +
geom_histogram(aes(y = ..count..),  # histogram
               binwidth = .02,
               color = "#252525",
               fill = "#deebf7",
               alpha = 0.6,
               position = "identity") +  # Ensure it's positioned correctly
geom_boxplot(aes(y = 0),
             fill = "white",
             color = "#08519c",
             alpha = 0.8,
             width = 0.5,
             position = position_dodge(width = 0.5),
             outlier.size = 0) +
geom_jitter(aes(y = 0),
            shape = 21,
            fill = "grey80",
            color = "#252525",
            width = 0.2,
            height = 0.1,
            alpha = 0.6) +
theme_minimal() +
labs(x = "Final.Outcome",
     y = "Count")
```

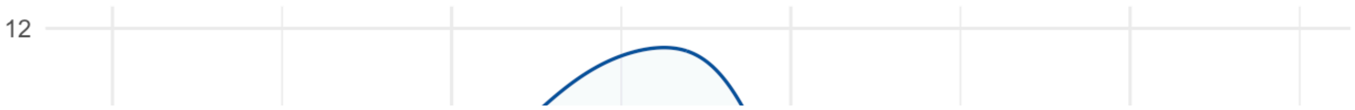

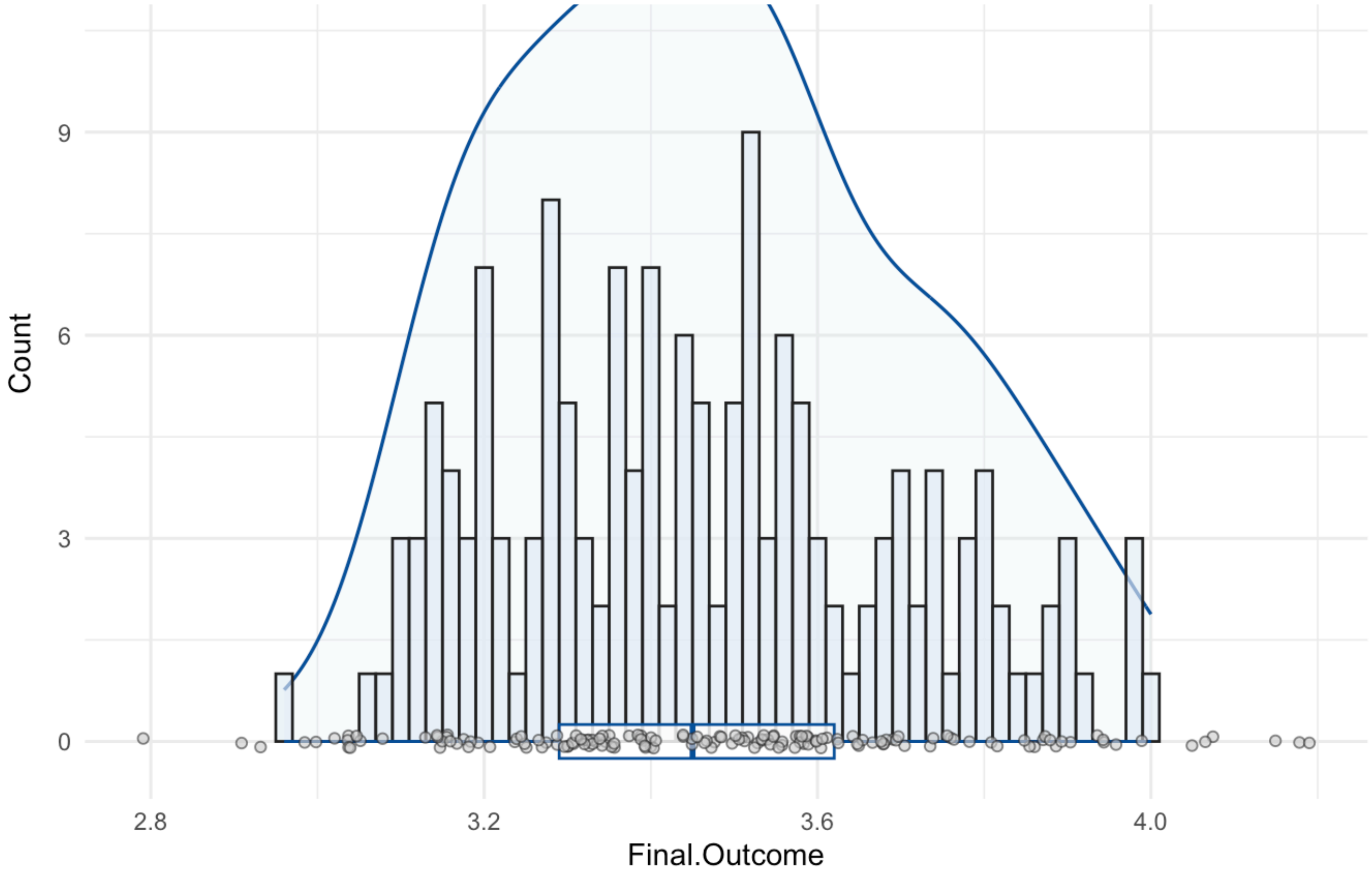

meta-analysis of the means

Using metafor we need to use the escalc() function to add yi (mean) and vi (variance) columns. Then we add another column (es.id) which indicates the number of data points in the dataset

```
data_means <- escalc(measure = "MN", # MN for meta analysis of means
               mi = Final.Outcome, # final outcome
               sdi = Final.SD, # final sd
               ni = Sample.Size, # sample size (n)
               data = data)  # specify the dataset

data_means$ES.id = 1:nrow(data_means) # add column with point etimate number
```

We use clubSandwich package to attain the covariance matrices considering the nested dataset (some studies have multiple groups, some groups provide multiple data points) assuming a correlation of r = 0.7

```
V_means <- impute_covariance_matrix(vi = data_means$vi,
                              cluster = data_means$Group.id, # using groups followi
ng J Pustejovsky discussion
```

```
                              r = .7,
                              smooth_vi = T)
```

We analyse the data with multilevel mixed effects meta analysis and robust estimate using the metafor package

```
rma_means_model <- rma.mv(yi, # overall three level model
                   V_means,
                   random = list(~ 1 | Covidence../Group.id/ES.id),
                   digits = 2,
                   data = data_means,
                   method = "REML",
                   test = "t",
                   level = .9,
                   control=list(optimizer="optim", optmethod="Nelder-Mead"))

robust_means_model <- robust.rma.mv(rma_means_model,
                              cluster = data_means$Covidence..,
                              adjust = T,
                              clubSandwich = T)
```

Model's summary

```
summ_robust_means_model <- summary(robust_means_model)
summ_robust_means_model
```

```
##
## Multivariate Meta-Analysis Model (k = 157; method: REML)
##
## [illegible]
```

```
##   logLik  Deviance        AIC        BIC       AICc
##    91.21   -182.42   -174.42   -162.22   -174.15
##
## Variance Components:
##
##             estim  sqrt  nlvls  fixed                       factor
## sigma^2.1   0.03  0.18     58     no                  Covidence..
## sigma^2.2   0.00  0.04    107     no         Covidence../Group.id
## sigma^2.3   0.00  0.06    157     no  Covidence../Group.id/ES.id
##
## Test for Heterogeneity:
## Q(df = 156) = 6739.31, p-val < .01
##
## Number of estimates:   157
## Number of clusters:    58
## Estimates per cluster: 1-14 (mean: 2.71, median: 2)
##
## Model Results:
##
## estimate    se¹    tval¹     df¹  pval¹  ci.lb¹  ci.ub¹
##     3.45  0.03   134.45   56.79   <.01    3.41    3.50   ***
##
## ---
## Signif. codes:  0 '***' 0.001 '**' 0.01 '*' 0.05 '.' 0.1 ' ' 1
##
## 1) results based on cluster-robust inference (var-cov estimator: CR2,
##    approx t-test and confidence interval, df: Satterthwaite approx)
```

Model's estimate, 90% confidence internals (90%CI) and prediction intervals (%90PI)

```
est_robust_means_model <- predict.rma(robust_means_model, digits = 2, level = .9)
est_robust_means_model
```

```
##
##  pred   se ci.lb ci.ub pi.lb pi.ub
##  3.45 0.03  3.40  3.50  3.12  3.79
```

Explore variance using sigma (σ) estimates

```
var_studies <- round(sqrt(robust_means_model$sigma2[1]),2)
var_groups <- round(sqrt(robust_means_model$sigma2[2]),2)
var_es <- round(sqrt(robust_means_model$sigma2[3]),2)
```

```
var_studies
```

```
## [1] 0.18
```

```
var_groups
```

```
## [1] 0.04
```

```
var_es
```

```
## [1] 0.06
```

Explore heterogeneity using I2 estimates

```
het_studies <- round(i2_ml(robust_means_model)[2],1)
het_groups <- round(i2_ml(robust_means_model)[3],1)
het_es <- round(i2_ml(robust_means_model)[4],1)

het_studies
```

```
## I2_Covidence..
##           84.2
```

```
het_groups
```

```
## I2_Covidence../Group.id
##                        4
```

```
het_es
```

```
## I2_Covidence../Group.id/ES.id
##                           9.8
```

meta-analysis of the standard deviations (SDs)

To analyse the variance between players (i.e., between-athlete variability), we employ a meta-analysis of the standard deviations (SDs) using log-transformed SD and adjusting for sample size

To explore the distribution of data points (SDs) and identify potential outliers, we visualise a rain cloud plot

To explore the distribution of data points (SDs) and identify potential outliers, we visualise a rain cloud plot with a histogram

```
ggplot(data, aes(x = Final.SD)) +
  geom_density(aes(y = ..density.. * max(..count..) / max(..density..)/20),
               color = "#08519c",
               fill = "#deebf7",
               alpha = 0.2,
               size = 0.6) +
  geom_histogram(aes(y = ..count..),  # histogram
                 binwidth = .02,
                 color = "#252525",
                 fill = "#deebf7",
                 alpha = 0.6,
                 position = "identity") +  # Ensure it's positioned correctly
  geom_boxplot(aes(y = 0),
               fill = "white",
               color = "#08519c",
               alpha = 0.8,
               width = 0.5,
               position = position_dodge(width = 0.5),
               outlier.size = 0) +
  geom_jitter(aes(y = 0),
              shape = 21,
              fill = "grey80",
              color = "#252525",
              width = 0.2,
              height = 0.1,
              alpha = 0.6) +
  theme_minimal() +
  labs(x = "Final.SD",
       y = "Count")
```

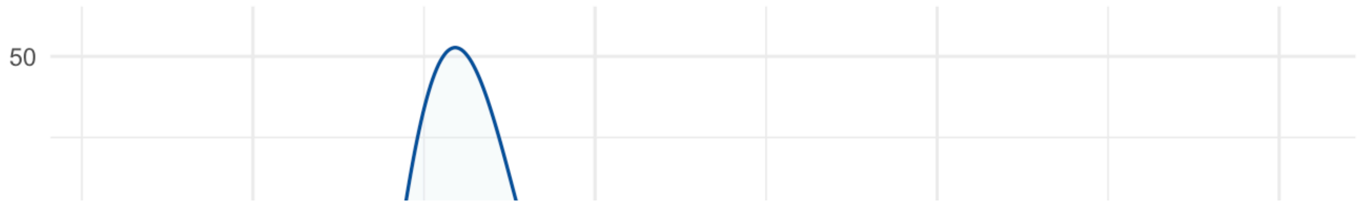

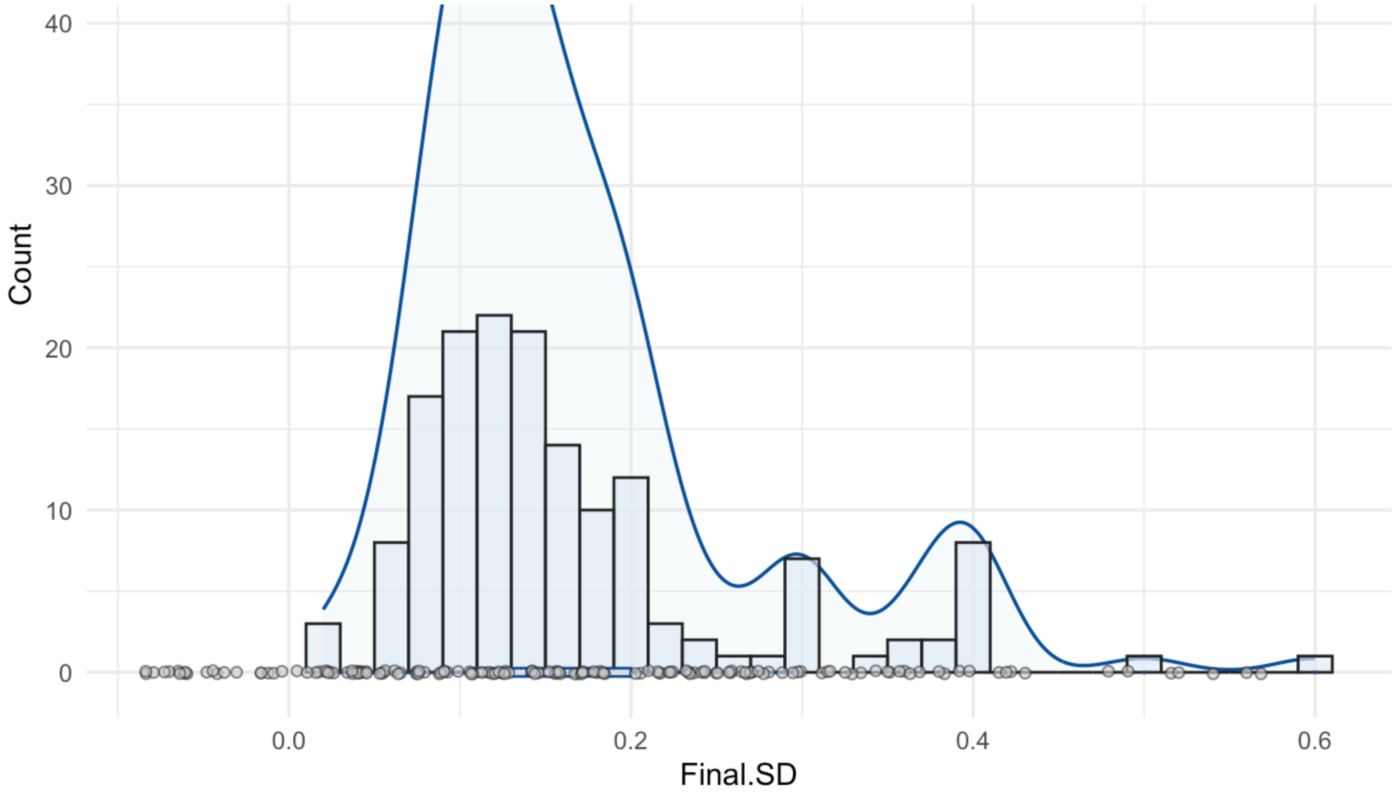

Using metafor we need to use the escalc() function to add yi (log-transformed SD) and vi (from n) columns

```
data_sd <- escalc(measure = "SDLN", # SDLN for meta analysis of SDs
               sdi = Final.SD, # final sd
               ni = Sample.Size, # sample size (n)
               data = data)  # specify the dataset

data_sd$ES.id = 1:nrow(data_sd) # add column with point etimate number
```

We use clubSandwich package to attain the covariance matrices considering the nested dataset (some studies have multiple groups, some groups provide multiple data points) assuming a correlation of r = 0.7

```
V_sd <- impute_covariance_matrix(vi = data_sd$vi,
                              cluster = data_sd$Group.id, # using groups following
J Pustejovsky discussion
                              r = .7,
                              smooth_vi = T)
```

We analyse the data with multilevel mixed effects meta analysis and robust estimate using the metafor package

```
rma_sd_model <- rma.mv(yi, # overall three level model
                    V_sd,
                    random = list(~ 1 | Covidence../Group.id/ES.id),
```

```
                    random = list(~ 1 | Covidence../Group.id/ES.id),
                    digits = 2,
                    data = data_sd,
                    method = "REML",
                    test = "t",
                    level = .9,
                    control=list(optimizer="optim", optmethod="Nelder-Mead"))

robust_sd_model <- robust.rma.mv(rma_sd_model,
                              cluster = data_sd$Covidence..,
                              adjust = T,
                              clubSandwich = T)
```

Model's summary

```
summ_robust_sd_model <- summary(robust_sd_model)
summ_robust_sd_model
```

```
##
## Multivariate Meta-Analysis Model (k = 157; method: REML)
##
##   logLik  Deviance       AIC       BIC      AICc
##   -70.27    140.53    148.53    160.73    148.80
##
```

```
## Variance Components:
##
##             estim  sqrt  nlvls  fixed                      factor
## sigma^2.1   0.19  0.44     58     no                 Covidence..
## sigma^2.2   0.03  0.17    107     no        Covidence../Group.id
## sigma^2.3   0.02  0.15    157     no  Covidence../Group.id/ES.id
##
## Test for Heterogeneity:
## Q(df = 156) = 866.29, p-val < .01
##
## Number of estimates:   157
## Number of clusters:    58
## Estimates per cluster: 1-14 (mean: 2.71, median: 2)
##
## Model Results:
##
## estimate    se¹    tval¹     df¹  pval¹  ci.lb¹  ci.ub¹
##    -1.92  0.07   -29.17   56.49   <.01   -2.03   -1.81   ***
##
## ---
## Signif. codes:  0 '***' 0.001 '**' 0.01 '*' 0.05 '.' 0.1 ' ' 1
##
## 1) results based on cluster-robust inference (var-cov estimator: CR2,
##    approx t-test and confidence interval, df: Satterthwaite approx)
```

Model's estimate, 90% confidence internals (90%CI) and prediction intervals (%90PI)

```
est_robust_sd_model <- predict.rma(robust_sd_model, digits = 2, transf = transf.ex
p.int, level = .9)
est_robust_sd_model
```

```
##
##  pred ci.lb ci.ub pi.lb pi.ub
##  0.15  0.13  0.17  0.06  0.34
```

meta-analysis results

Let's create a summary table of the meta-analysis results

```
overall_summary <- data.frame(final_estimate = round(est_robust_means_model$pred,
2),
                 ci = paste(round(est_robust_means_model$ci.lb,2), round(est_robust
_means_model$ci.ub,2),
                            sep = " to "),
                 pi = paste(round(est_robust_means_model$pi.lb,2), round(est_robust
[illegible]
```

```
_means_model$pi.ub,2),
                             sep = " to "),
                 blank = '',
                 sd = round(est_robust_sd_model$pred,2),
                 ci = paste(round(est_robust_sd_model$ci.lb,2), round(est_robust_sd
_model$ci.ub,2),
                            sep = " to "))

rownames(overall_summary) <- NULL

kable(
  overall_summary,
  caption = "A summary table of meta-analysis results",
  align = c("c","c","c","c","c","c"),
  col.names = c('Estimate',
              '90% CI',
              "90% PI",
              "",
              "SD",
              "90% CI")) %>%
  row_spec(0, bold = TRUE) %>%
 kable_styling(bootstrap_options = c("striped", "hover"), full_width = F) %>%
  column_spec(1:6, background = "", color = "#252525") %>%
  column_spec(4, width = "1.2cm") %>%  # Hide the empty column
  kable_styling(latex_options = "HOLD_position")
```

A summary table of meta-analysis results

Estimate	90% CI	90% PI		SD	90% CI
3.45	3.4 to 3.5	3.12 to 3.79		0.15	0.13 to 0.17

meta-regression of the means to assess performance in distinct performance tiers

We first explore the number of estimates, number of unique groups, and number of unique studies across tiers

```
data_means <- data_means %>%
  mutate(Tier.Mod = case_when(
    Tier.Mod == "Tier 4" ~ "Tier 4 and 5",
    Tier.Mod == "Tier 5" ~ "Tier 4 and 5",
    TRUE ~ Tier.Mod
  ))
```

```
data_sd <- data_sd %>%
  mutate(Tier.Mod = case_when(
    Tier.Mod == "Tier 4" ~ "Tier 4 and 5",
    Tier.Mod == "Tier 5" ~ "Tier 4 and 5",
    TRUE ~ Tier.Mod
  ))

tier_summary <- data %>%
  group_by(Tier.Mod) %>%
  summarise(
    ES = n(),
    Groups = n_distinct(Group.id),
    Studies = n_distinct(`Covidence.#`),
    overall_n = sum(Sample.Size))

kable(
  tier_summary,
  caption = "Summary table of sprint performance in different tiers",
  align = c("c","c","c","c","c","c"),
  col.names = c('Tier',
                'No. Estimates',
                'No. Groups',
                'No. Studies',
                'Overall N')) %>%
  row_spec(0, bold = T) %>%
  column_spec(1, bold = F, color = "#585858") %>%
  kable_styling(bootstrap_options = c("hover"), full_width = F)
```

Summary table of sprint performance in different tiers

**Tier**	**No. Estimates**	**No. Groups**	**No. Studies**	**Overall N**
Tier 2	31	15	10	464
Tier 3	96	66	37	1611
Tier 4	27	22	12	383
Tier 5	3	3	3	131

We analyse the data with a similar multilevel mixed effects model while adding tier as a moderator

```
rma_means_tier_model <- rma.mv(yi, # overall three level model
                    V_means,
                    mods = ~ Tier.Mod, # moderators using tiers
                    random = list(~ 1 | Covidence../Group.id/ES.id),
                    digits = 2,
                    data = data_means,
```

```
                    method = "REML",
                    test = "t",
                    level = .9,
                    control=list(optimizer="optim", optmethod="Nelder-Mead"))

robust_means_tier_model <- robust.rma.mv(rma_means_tier_model,
                              cluster = data_means$Covidence..,
                              adjust = T,
                              clubSandwich = T)
```

Model's summary

```
summ_robust_means_tier_model <- summary(robust_means_tier_model)
summ_robust_means_tier_model
```

```
##
## Multivariate Meta-Analysis Model (k = 157; method: REML)
##
##   logLik  Deviance       AIC       BIC      AICc
##    96.33   -192.66   -180.66   -162.44   -180.09
##
## Variance Components:
##
```

```
##             estim  sqrt  nlvls  fixed                      factor
## sigma^2.1    0.03  0.17     58     no                 Covidence..
## sigma^2.2    0.00  0.03    107     no        Covidence../Group.id
## sigma^2.3    0.00  0.06    157     no  Covidence../Group.id/ES.id
##
## Test for Residual Heterogeneity:
## QE(df = 154) = 6678.93, p-val < .01
##
## Number of estimates:   157
## Number of clusters:    58
## Estimates per cluster: 1-14 (mean: 2.71, median: 2)
##
## Test of Moderators (coefficients 2:3):¹
## F(df1 = 2, df2 = 4.97) = 14.15, p-val < .01
##
## Model Results:
##
##                        estimate    se¹   tval¹    df¹  pval¹  ci.lb¹  ci.ub¹
## intrcpt                    3.61  0.06   61.77    7.6   <.01    3.50    3.72
## Tier.ModTier 3            -0.17  0.06   -2.84   9.03   0.02   -0.28   -0.06
## Tier.ModTier 4 and 5      -0.22  0.06   -3.85   6.99   <.01   -0.32   -0.11
##
## intrcpt                ***
## Tier.ModTier 3           *
## Tier.ModTier 4 and 5    **
##
## ---
## Signif. codes:  0 '***' 0.001 '**' 0.01 '*' 0.05 '.' 0.1 ' ' 1
##
## 1) results based on cluster-robust inference (var-cov estimator: CR2,
##    approx t/F-tests and confidence intervals, df: Satterthwaite approx)
```

Model's estimate, 90% confidence internals (90%CI) and prediction intervals (%90PI)

```
est_robust_means_tier2_model <- predict.rma(robust_means_tier_model, newmods = c(0,
0), digits = 2, addx = T, level = .9) # tier 2
est_robust_means_tier2_model
```

```
##
##  pred   se ci.lb ci.ub pi.lb pi.ub X.intrcpt X.Tier.ModTier.3
##  3.61 0.06  3.47  3.74  3.24  3.97         1                0
##  X.Tier.ModTier.4.and.5
##                       0
```

```
est_robust_means_tier3_model <- predict.rma(robust_means_tier_model, newmods = c(1,
0), digits = 2, addx = T, level = .9) # tier 3
```

```
est_robust_means_tier3_model
```

```
##
##  pred   se ci.lb ci.ub pi.lb pi.ub X.intrcpt X.Tier.ModTier.3
##  3.43 0.03  3.38  3.49  3.11  3.75         1                1
##  X.Tier.ModTier.4.and.5
##                        0
```

```
est_robust_means_tier4and5_model <- predict.rma(robust_means_tier_model, newmods =
c(0,1), digits = 2, addx = T, level = .9) # tier 4 and 5
est_robust_means_tier4and5_model
```

```
##
##  pred   se ci.lb ci.ub pi.lb pi.ub X.intrcpt X.Tier.ModTier.3
##  3.39 0.02  3.34  3.44  3.05  3.73         1                0
##  X.Tier.ModTier.4.and.5
##                        1
```

Arrange the results to obtain dataset including individual estimates including id and weight/size

```
data_means_clean <- data_means %>% filter(Tier.Mod != "NA" & Final.Outcome != "NA")
# clean the data for missing values

data_long_tiers <- data.frame(covidence = data_means_clean$Covidence..,
                ref = paste(data_means_clean$Lead.Author, data_means_clean$Year, se
p = " "),
                 yi = data_means_clean$yi,
                 weight = weights.rma.mv(robust_means_tier_model),
                 n = data_means_clean$Sample.Size,
```

```
                  tier_ref = data_means_clean$Tier.Mod,
                 test = "20m")

pred_tier2 <- est_robust_means_tier2_model$pred[1] # tier 2
ci.lb_tier2 <-  est_robust_means_tier2_model$ci.lb[1]
ci.ub_tier2 <-  est_robust_means_tier2_model$ci.ub[1]
pi.lb_tier2 <- est_robust_means_tier2_model$pi.lb[1]
pi.ub_tier2 <- est_robust_means_tier2_model$pi.ub[1]

pred_tier3 <- est_robust_means_tier3_model$pred[1] # tier 3
ci.lb_tier3 <-  est_robust_means_tier3_model$ci.lb[1]
ci.ub_tier3 <-  est_robust_means_tier3_model$ci.ub[1]
pi.lb_tier3 <- est_robust_means_tier3_model$pi.lb[1]
pi.ub_tier3 <- est_robust_means_tier3_model$pi.ub[1]

pred_tier4and5 <- est_robust_means_tier4and5_model$pred[1] # tier 4 and 5
ci.lb_tier4and5 <-  est_robust_means_tier4and5_model$ci.lb[1]
ci.ub_tier4and5 <-  est_robust_means_tier4and5_model$ci.ub[1]
pi.lb_tier4and5 <- est_robust_means_tier4and5_model$pi.lb[1]
pi.ub_tier4and5 <- est_robust_means_tier4and5_model$pi.ub[1]
```

meta-regression of the SD in distinct performance tiers

We analyse the data with a similar multilevel mixed effects model while adding tier as a moderator

```
rma_sd_tier_model <- rma.mv(yi, # overall three level model
                    V_sd,
                    mods = ~ Tier.Mod, # moderators using tiers
                    random = list(~ 1 | Covidence../Group.id/ES.id),
                    digits = 2,
                    data = data_sd,
                    method = "REML",
                    test = "t",
                    level = .9,
                    [illegible]
```

```
                    control=list(optimizer="optim", optmethod="Nelder-Mead"))

robust_sd_tier_model <- robust.rma.mv(rma_sd_tier_model,
                              cluster = data_means$Covidence..,
                              adjust = T,
                              clubSandwich = T)
```

Model's summary

```
summ_robust_sd_tier_model <- summary(robust_sd_tier_model)
summ_robust_sd_tier_model
```

```
##
## Multivariate Meta-Analysis Model (k = 157; method: REML)
##
##   logLik  Deviance       AIC       BIC      AICc
##   -67.63    135.26    147.26    165.48    147.83
##
## Variance Components:
##
##            estim  sqrt  nlvls  fixed                     factor
## sigma^2.1   0.19  0.43     58     no                Covidence..
```

```
## sigma^2.2   0.03  0.17    107     no        Covidence../Group.id
## sigma^2.3   0.02  0.15    157     no  Covidence../Group.id/ES.id
##
## Test for Residual Heterogeneity:
## QE(df = 154) = 799.18, p-val < .01
##
## Number of estimates:   157
## Number of clusters:    58
## Estimates per cluster: 1-14 (mean: 2.71, median: 2)
##
## Test of Moderators (coefficients 2:3):¹
## F(df1 = 2, df2 = 9.29) = 1.18, p-val = 0.35
##
## Model Results:
##
##                        estimate    se¹   tval¹     df¹  pval¹  ci.lb¹  ci.ub¹
## intrcpt                   -1.71  0.22   -7.63     8.9   <.01   -2.12   -1.30
## Tier.ModTier 3            -0.23  0.23   -0.97   12.99   0.35   -0.64    0.19
## Tier.ModTier 4 and 5      -0.33  0.23   -1.42   12.73   0.18   -0.73    0.08
##
## intrcpt                ***
## Tier.ModTier 3
## Tier.ModTier 4 and 5
##
## ---
## Signif. codes:  0 '***' 0.001 '**' 0.01 '*' 0.05 '.' 0.1 ' ' 1
##
## 1) results based on cluster-robust inference (var-cov estimator: CR2,
##    approx t/F-tests and confidence intervals, df: Satterthwaite approx)
```

Model's estimate, 90% confidence internals (90%CI) and prediction intervals (%90PI)

```
est_robust_sd_tier2_model <- predict.rma(robust_sd_tier_model, newmods = c(0,0), tr
ansf = transf.exp.int, digits = 2, addx = T, level = .9) # tier 2
est_robust_sd_tier2_model
```

```
##
##  pred ci.lb ci.ub pi.lb pi.ub X.intrcpt X.Tier.ModTier.3 X.Tier.ModTier.4.and.5
##  0.18  0.11  0.30  0.07  0.49         1                0                      0
```

```
est_robust_sd_tier3_model <- predict.rma(robust_sd_tier_model, newmods = c(1,0), tr
ansf = transf.exp.int, digits = 2, addx = T, level = .9) # tier 3
est_robust_sd_tier3_model
```

```
##
```

```
##  pred ci.lb ci.ub pi.lb pi.ub X.intrcpt X.Tier.ModTier.3 X.Tier.ModTier.4.and.5
##  0.14  0.12  0.17  0.06  0.33         1                1                      0
```

```
est_robust_sd_tier4and5_model <- predict.rma(robust_sd_tier_model, newmods = c(0,
1), transf = transf.exp.int, digits = 2, addx = T, level = .9) # tier 4 and 5
est_robust_sd_tier4and5_model
```

```
##
##  pred ci.lb ci.ub pi.lb pi.ub X.intrcpt X.Tier.ModTier.3 X.Tier.ModTier.4.and.5
##  0.13  0.11  0.16  0.05  0.32         1                0                      1
```

Visualise the results using strip chart and bubbles indicating the weights of studies

```
ggplot(data_long_tiers, aes(x = tier_ref, y = yi)) +
  geom_point(aes(size = weight, text = paste("ref:", ref, "<br>test:", test)),
             shape = 21, color = "#d9d9d9", fill = "#f7f7f7", alpha = 0.8, position
= position_jitter(width = .08, height = 0)) +
  #scale_size_continuous(range = c(4,4)) +
  scale_y_continuous(limits = c(2.8,4.2),
                     breaks = seq(2.80,4.2,.2)) +
  labs(title = "",
       x = "",
       y = "Time (s)",
       size = "Size",
       color = "Tier") +
  geom_errorbar(aes(ymin = ci.lb_tier2, ymax = ci.lb_tier2), data = subset(data_lon
g_tiers, tier_ref == 'Tier 2'), size = .8, width = .05, color = "grey20") +
  geom_errorbar(aes(ymin = ci.ub_tier2, ymax = ci.ub_tier2), data = subset(data_lon
g_tiers, tier_ref == 'Tier 2'), size = .6, width = .05, color = "grey20") +
  geom_errorbar(aes(ymin = ci.lb_tier2, ymax = ci.ub_tier2), data = subset(data_lon
g_tiers, tier_ref == 'Tier 2'), size = .4, width = .05, color = "grey20", linetype
= "dotted") +

  geom_errorbar(aes(ymin = ci.lb_tier3, ymax = ci.lb_tier3), data = subset(data_lon
g_tiers, tier_ref == 'Tier 3'), size = .8, width = .05, color = "grey20") +
  geom_errorbar(aes(ymin = ci.ub_tier3, ymax = ci.ub_tier3), data = subset(data_lon

g_tiers, tier_ref == 'Tier 3'), size = .6, width = .05, color = "grey20") +
  geom_errorbar(aes(ymin = ci.lb_tier3, ymax = ci.ub_tier3), data = subset(data_lon
g_tiers, tier_ref == 'Tier 3'), size = .4, width = .05, color = "grey20", linetype
= "dotted") +

  geom_errorbar(aes(ymin = ci.lb_tier4and5, ymax = ci.lb_tier4and5), data = subset(
data_long_tiers, tier_ref == 'Tier 4 and 5'), size = .8, width = .05, color = "grey
20") +
  geom_errorbar(aes(ymin = ci.ub_tier4and5, ymax = ci.ub_tier4and5), data = subset(
data_long_tiers, tier_ref == 'Tier 4 and 5'), size = .6, width = .05, color = "grey
20") +
  geom_errorbar(aes(ymin = ci.lb_tier4and5, ymax = ci.ub_tier4and5), data = subset(
```

```
  geom_errorbar(aes(ymin = ci.lb_tier4and5, ymax = ci.ub_tier4and5), data = subset(
data_long_tiers, tier_ref == 'Tier 4 and 5'), size = .4, width = .05, color = "grey
20", linetype = "dotted") +

  geom_errorbar(aes(ymin = pred_tier2, ymax = pred_tier2), data = subset(data_long_
tiers, tier_ref == 'Tier 2'), size = 1, width = 0.1, color = "black") +
  geom_errorbar(aes(ymin = pred_tier3, ymax = pred_tier3), data = subset(data_long_
tiers, tier_ref == 'Tier 3'), size = 1, width = 0.1, color = "black") +
  geom_errorbar(aes(ymin = pred_tier4and5, ymax = pred_tier4and5), data = subset(da
ta_long_tiers, tier_ref == 'Tier 4 and 5'), size = 1, width = 0.1, color = "black")
+

  geom_errorbar(aes(ymin = pi.lb_tier2, ymax = pi.lb_tier2), data = subset(data_lon
g_tiers, tier_ref == 'Tier 2'), size = .4, width = .06, color = "grey25", linetype
= "dotdash") +
  geom_errorbar(aes(ymin = pi.lb_tier3, ymax = pi.lb_tier3), data = subset(data_lon
g_tiers, tier_ref == 'Tier 3'), size = .4, width = .06, color = "grey25", linetype
= "dotdash") +
  geom_errorbar(aes(ymin = pi.lb_tier4and5, ymax = pi.lb_tier4and5), data = subset(
data_long_tiers, tier_ref == 'Tier 4 and 5'), size = .4, width = .06, color = "grey
25", linetype = "dotdash") +

  geom_errorbar(aes(ymin = pi.ub_tier2, ymax = pi.ub_tier2), data = subset(data_lon
g_tiers, tier_ref == 'Tier 2'), size = .4, width = .06, color = "grey25", linetype
= "dotdash") +
  geom_errorbar(aes(ymin = pi.ub_tier3, ymax = pi.ub_tier3), data = subset(data_lon
g_tiers, tier_ref == 'Tier 3'), size = .4, width = .06, color = "grey25", linetype
= "dotdash") +
  geom_errorbar(aes(ymin = pi.ub_tier4and5, ymax = pi.ub_tier4and5), data = subset(
data_long_tiers, tier_ref == 'Tier 4 and 5'), size = .4, width = .06, color = "grey
25", linetype = "dotdash") +

  geom_text(aes(x = 1, y = max(data_long_tiers$yi)+.2, label = paste(round(pred_tie
r2,2), " ±", " ", round(est_robust_sd_tier2_model$pred,2), sep = "")), color = "#25
2525", size = 3.2, fontface = 2, family = "serif") +
  geom_text(aes(x = 1, y = max(data_long_tiers$yi)+.1, label = paste(" [", round(c
i.lb_tier2,2), " to ", round(ci.ub_tier2,2), "]", sep = "")), color = "#252525", si
ze = 3.2, fontface = 2, family = "serif") +

  geom_text(aes(x = 2, y = max(data_long_tiers$yi)+.2, label = paste(round(pred_tie
r3,2), " ±", " ", round(est_robust_sd_tier3_model$pred,2), sep = "")), color = "#25
2525", size = 3.2, fontface = 2, family = "serif") +
  geom_text(aes(x = 2, y = max(data_long_tiers$yi)+.1, label = paste(" [", round(c
i.lb_tier3,2), " to ", round(ci.ub_tier3,2), "]", sep = "")), color = "#252525", si
ze = 3.2, fontface = 2, family = "serif") +

  geom_text(aes(x = 3, y = max(data_long_tiers$yi)+.2, label = paste(round(pred_tie
r4and5,2), " ±", " ", round(est_robust_sd_tier4and5_model$pred,2), sep = "")), colo
r = "#252525", size = 3.2, fontface = 2, family = "serif") +
  geom_text(aes(x = 3, y = max(data_long_tiers$yi)+.1, label = paste(" [", round(c
i.lb_tier4and5,2), " to ", round(ci.ub_tier4and5,2), "]", sep = "")), color = "#252
525", size = 3.2, fontface = 2, family = "serif") +
```

```
  guides(x = "axis_truncated", y = "axis_truncated") +
  theme(axis.line = element_line(colour = "black"),
        panel.background = element_blank(),
        axis.text.x = element_text(face = "bold", size = 12),
        axis.text.y = element_text(face = "plain", size = 10),
        axis.ticks.x = element_blank(),
        plot.title = element_text(size = 14, face = 2, hjust = .05),
        legend.position = "none")
```

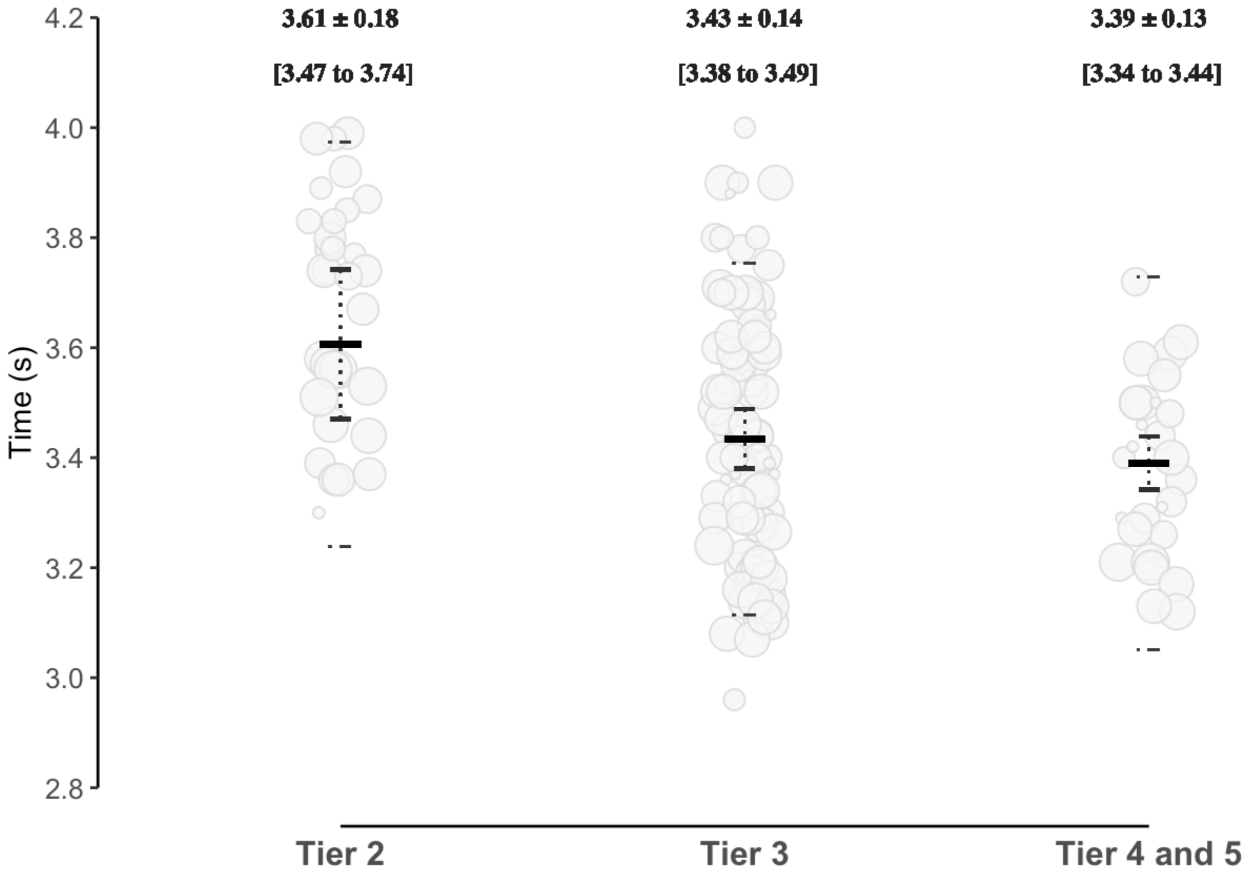

meta-regression results

Let's create a summary table of the meta-analysis results

```
overall_summary_tiers <- data.frame(
  Tier = c('Tier 2', 'Tier 3', 'Tier 4 and 5'),
  final_estimate = c(
    round(est_robust_means_tier2_model$pred, 2),  # estimate tier 2
    round(est_robust_means_tier3_model$pred, 2),  # estimate tier 3
    round(est_robust_means_tier4and5_model$pred, 2)   # estimate tier 4 and 5
  ),
  blank = c('', '', ''),
  sd = c(
    round(est_robust_sd_tier2_model$pred, 2),  # sd tier 2
```

```
    round(est_robust_sd_tier3_model$pred, 2),  # sd tier 3
    round(est_robust_sd_tier4and5_model$pred, 2)   # sd tier 4 and 5
  )
)

rownames(overall_summary) <- NULL

kable(
  overall_summary_tiers,
  caption = "A summary table of meta-regression main estimates",
  align = c("c","c","c","c","c","c"),
  col.names = c('Tier',
              'Estimate',
              "",
              "SD")) %>%
 row_spec(0, bold = TRUE) %>%
 kable_styling(bootstrap_options = c("striped", "hover"), full_width = F) %>%
  column_spec(1:3, background = "", color = "#252525") %>%
  column_spec(1:2, width = "2.8cm") %>%  # Hide the empty column
  column_spec(4, width = "2.8cm") %>%  # Hide the empty column
  kable_styling(latex_options = "HOLD_position")
```

A summary table of meta-regression main estimates

Tier	Estimate	SD
Tier 2	3.61	0.18
Tier 3	3.43	0.14
Tier 4 and 5	3.39	0.13

Write dataset for Shiny dashboard

```
write_data_meta_20m_overall <-

  data.frame(
 final.means = c(est_robust_means_model$pred, # pooled mean estimate overall meta
                pred_tier2, # pooled estimate overall meta tier 2
                pred_tier3, # pooled estimate overall meta tier 3
                pred_tier4and5), # pooled estimate overall meta tier 4 and 5
  means.lb.ci = c(est_robust_means_model$ci.lb, # lower bound ci overall meta
                ci.lb_tier2, # lower bound ci overall meta tier 2
                ci.lb_tier3, # lower bound ci overall meta tier 3
                ci.lb_tier4and5), # lower bound ci overall meta tier 4 and 5
  means.ub.ci = c(est_robust_means_model$ci.ub, # lower bound ci overall meta
                ci.ub_tier2, # lower bound ci overall meta tier 2
                ci.ub_tier3, # lower bound ci overall meta tier 3
                ci.ub_tier4and5), # lower bound ci overall meta tier 4 and 5
```

```
  means.lb.pi = c(est_robust_means_model$pi.lb, # upper bound pi overall meta
                  pi.lb_tier2, # lower bound pi overall meta tier 2
                  pi.lb_tier3, # lower bound pi overall meta tier 3
                  pi.lb_tier4and5), # lower bound pi overall meta tier 4 and 5
  means.ub.pi = c(est_robust_means_model$pi.ub, # upper bound ci overall meta
                  pi.ub_tier2, # upper bound ci overall meta tier 2
                  pi.ub_tier3, # upper bound ci overall meta tier 3
                  pi.ub_tier4and5), # upper bound ci overall meta tier 4 and 5
  final_sd = c(est_robust_sd_model$pred, # pooled sd estimate overall meta
                  est_robust_sd_tier2_model$pred, # pooled sd overall meta tier 2
                  est_robust_sd_tier3_model$pred, # pooled sd overall meta tier 3
                  est_robust_sd_tier4and5_model$pred), # pooled sd overall meta tie
r 4 and 5
  sd.lb.ci = c(est_robust_sd_model$ci.lb, # lower bound ci overall meta
                  est_robust_sd_tier2_model$ci.lb, # lower bound ci overall meta ti
er 2
                  est_robust_sd_tier3_model$ci.lb, # lower bound ci overall meta ti
er 3
                  est_robust_sd_tier4and5_model$ci.lb), # lower bound ci overall me
ta tier 4 and 5
  sd.ub.ci = c(est_robust_sd_model$ci.ub, # upper bound ci overall meta
                  est_robust_sd_tier2_model$ci.ub, # upper bound ci overall meta ti
er 2
                  est_robust_sd_tier3_model$ci.ub, # upper bound ci overall meta ti
er 3
                  est_robust_sd_tier4and5_model$ci.ub), # upper bound ci overall me
ta tier 4 and 5
  sd.lb.pi = c(est_robust_sd_model$pi.lb, # lower bound pi overall meta
                  est_robust_sd_tier2_model$pi.lb, # lower bound pi overall meta ti
er 2
                  est_robust_sd_tier3_model$pi.lb, # lower bound pi overall meta ti
er 3
                  est_robust_sd_tier4and5_model$pi.lb), # lower bound pi overall me
ta tier 4 and 5
  sd.ub.pi = c(est_robust_sd_model$pi.ub, # upper bound pi overall meta
                  est_robust_sd_tier2_model$pi.ub, # upper bound pi overall meta ti
er 2
                  est_robust_sd_tier3_model$pi.ub, # upper bound pi overall meta ti
er 3
                  est_robust_sd_tier4and5_model$pi.ub), # upper bound pi overall me
ta tier 4 and 5
  test = rep("20m", 4), # name test
  analysis = c("whole", "tier2", "tier3", "tier4and5")) %>% # name analysis
  view()

write_data_meta_20m_long <- data_long_tiers
```
