# Supplementary material for: Benchmarking the Physical Performance Qualities in Women’s Football: A Systematic Review and Meta-analysis Across the Performance Scale
Source: Sports Med. 2025 Sep 1;56(Suppl 1):127–55. doi: 10.1007/s40279-025-02251-0 (PMC13314896; doi:10.1007/s40279-025-02251-0)
Supplement: Supplementary file 6 — Supplementary file6 (DOCX 28 KB) [file 40279_2025_2251_MOESM6_ESM.docx]

**Title:** Benchmarking The Physical Performance Qualities in Women’s Football: A Systematic Review and Meta-Analysis Across the Performance Scale

**Authors:**

Heidi R. Compton^1,2,3^ - 0000-0002-5818-4450

Ric Lovell^3,4^ - 0000-0001-5859-0267

Dawn Scott^3^ - 0009-0000-6763-1235

Jo Clubb^3,5^ - 0000-0002-6509-7531

Tzlil Shushan^3,4^ - 0000-0002-0544-1986

**Affiliations:**

^1^ School of Biomedical Sciences and Pharmacy, University of Newcastle, Australia;

^2^ Applied Sport Science and Exercise Testing Laboratory, University of Newcastle, Ourimbah, Australia;

^3^ FIFA, Women’s Development Programme, Women’s Football Division, Zurich, Switzerland;

^4^ Faculty of Science, Medicine and Health, University of Wollongong, Australia;

^5^ Global Performance Insights Ltd, London, United Kingdom

**Corresponding author:**

Heidi Compton

[Heidi.compton@newcastle.edu.au](mailto:Heidi.thornton@newcastle.edu.au)

University of Newcastle

Callaghan, Australia

| **Table S5** meta-analyses heterogeneity and variance |
| --- |
| **Protocol and procedure Heterogeneity (*I^2^*) between… Variance (𝜎, 90% CI) between…**  **Studies Groups/Studies Estimates/Groups Studies Groups/Studies Estimates/Groups** |
| **Cardiorespiratory fitness** |

| $\dot{V}$O_2_ max | 86.6% | 0% | 11.4% | 5.2 | 0 | 1.9 |
| --- | --- | --- | --- | --- | --- | --- |
| YYIRL1 | 71.5% | 14.4% | 12.0% | 297 | 133 | 122 |
| V_IFT_ | 89.1% | 0% | 9.2% | 1.7 | 0 | 0.6 |
| _V_$\dot{V}$O_2_ max | 85.3% | 6.6% | 5.0% | 1.2 | 0.3 | 0.3 |
| YYIRL2 | 49.3% | 48.7% | 0.3% | 123 | 122 | 10 |
| Set distance/time trial | 92.6% | 3.1% | 1.9% | 1.4 | 0.2 | 0.2 |
| VAM-EVAL | – | – | – | – | – | – |
| **Acceleration** |  |  |  |  |  |  |
| 5 m | 91.4% | 0% | 7.9% | 0.13 | 0 | 0.04 |
| 10 m | 75.4% | 0% | 22.5% | 0.11 | 0 | 0.06 |
| 15 m | 84.2% | 1.8% | 13.2% | 0.25 | 0.04 | 0.10 |
| 5 yards | – | – | – | – | – | – |
| 10 yards | – | – | – | – | – | – |
| 15 yards | – | – | – | – | – | – |
| **Sprint** |  |  |  |  |  |  |
| 20 m | 84.6% | 3.4% | 10.1% | 0.18 | 0.04 | 0.06 |
| 25 m | 54.4% | 5.9% | 34.7% | 0.10 | 0.03 | 0.08 |
| 30 m | 80.6% | 2.9% | 14.6% | 0.26 | 0.05 | 0.11 |
| 40 m | 74.3% | 17.3% | 5.0% | 0.25 | 0.12 | 0.07 |
| 20 yards | 75.7% | 17.5% | 0% | 0.07 | 0.04 | 0 |
| 25 yards | – | – | – | – | – | – |
| 40 yards | 96.8% | 0% | 2.3% | 0.48 | 0 | 0.07 |
| **Change of direction** |  |  |  |  |  |  |
| 5-0-5 | 88.7% | 0.8% | 10.1% | 0.43 | 0.04 | 0.15 |

| Illinois |  | 77.9% | 15.1% | 5.3% | 0.80 | 0.35 | 0.21 |
| --- | --- | --- | --- | --- | --- | --- | --- |
| T-test |  | 54.6% | 0% | 44.1% | 0.90 | 0 | 0.81 |
| **Maximal velocity** |  |  |  |  |  |  |  |
| Measured |  | 87.9% | 4.0% | 6.9% | 2.3 | 0.5 | 0.6 |
| Observed |  | 81.9% | 14.6% | 1.7% | 2.3 | 1.0 | 0.3 |
| **Lower limb strength** |  |  |  |  |  |  |  |
| 1 RM back squat |  | 81.9% | 0% | 16.1% | 18.3 | 0 | 8.1 |
| 1 RM deadlift |  | – | – | – | – | – | – |
| **Lower limb power** |  |  |  |  |  |  |  |
| SJ Force plate | Restricted | 93.4% | 0% | 3.8% | 3.9 | 0 | 0.8 |
|  | Unrestricted | – | – | – | – | – | – |
| Optical/contact | Restricted | 83.2% | 6.4% | 7.0% | 4.2 | 1.2 | 1.2 |
|  | Unrestricted | – | – | – | – | – | – |
| CMJ Force plate | Restricted | 85.6% | 0.6% | 8.7% | 3.4 | 0.3 | 1.1 |
|  | Unrestricted | 57.0% | 0% | 36.0% | 2.4 | 0 | 1.9 |
| Optical/contact | Restricted | 92.1% | 3.0% | 4.2% | 6.4 | 1.2 | 1.4 |
|  | Unrestricted | 95.1% | 2.2% | 1.5% | 10.3 | 1.6 | 1.3 |
| BJ | Unrestricted | 96.2% | 2.0% | 0.9% | 28.3 | 4.1 | 2.7 |

$\dot{V}$O_2_ max: maximal oxygen uptake, YYIRL1: Yo-Yo Intermittent Recovery Test Level 1, YYIRL2: Yo-Yo Intermittent Recovery Test Level 2, V_IFT_: final velocity attained during 30-15 Intermittent Fitness Test, _V_$\dot{V}$O_2_ max: velocity attained during graded maximal exercise tests, 1 RM: one repetition maximum, SJ: squat jump, CMJ: countermovement jump, BJ: broad jump, CI: confidence intervals
