# Supplementary material for: Benchmarking the Physical Performance Qualities in Women’s Football: A Systematic Review and Meta-analysis Across the Performance Scale
Source: Sports Med. 2025 Sep 1;56(Suppl 1):127–55. doi: 10.1007/s40279-025-02251-0 (PMC13314896; doi:10.1007/s40279-025-02251-0)
Supplement: Supplementary file 7 — Supplementary file7 (DOCX 74923 KB) [file 40279_2025_2251_MOESM7_ESM.docx]

**Title:** Benchmarking The Physical Performance Qualities in Women’s Football: A Systematic Review and Meta-Analysis Across the Performance Scale

**Authors:**

Heidi R. Compton^1,2,3^ - 0000-0002-5818-4450

Ric Lovell^3,4^ - 0000-0001-5859-0267

Dawn Scott^3^ - 0009-0000-6763-1235

Jo Clubb^3,5^ - 0000-0002-6509-7531

Tzlil Shushan^3,4^ - 0000-0002-0544-1986

**Affiliations:**

^1^ School of Biomedical Sciences and Pharmacy, University of Newcastle, Australia;

^2^ Applied Sport Science and Exercise Testing Laboratory, University of Newcastle, Ourimbah, Australia;

^3^ FIFA, Women’s Development Programme, Women’s Football Division, Zurich, Switzerland;

^4^ Faculty of Science, Medicine and Health, University of Wollongong, Australia;

^5^ Global Performance Insights Ltd, London, United Kingdom

**Corresponding author:**

Heidi Compton

Heidi.compton@newcastle.edu.au

University of Newcastle

Callaghan, Australia

**Figure S1.** Funnel plots for small-study effects with confidence levels of 90% (white), 95% (dark grey) and 99% (light grey) for all protocols and procedures.
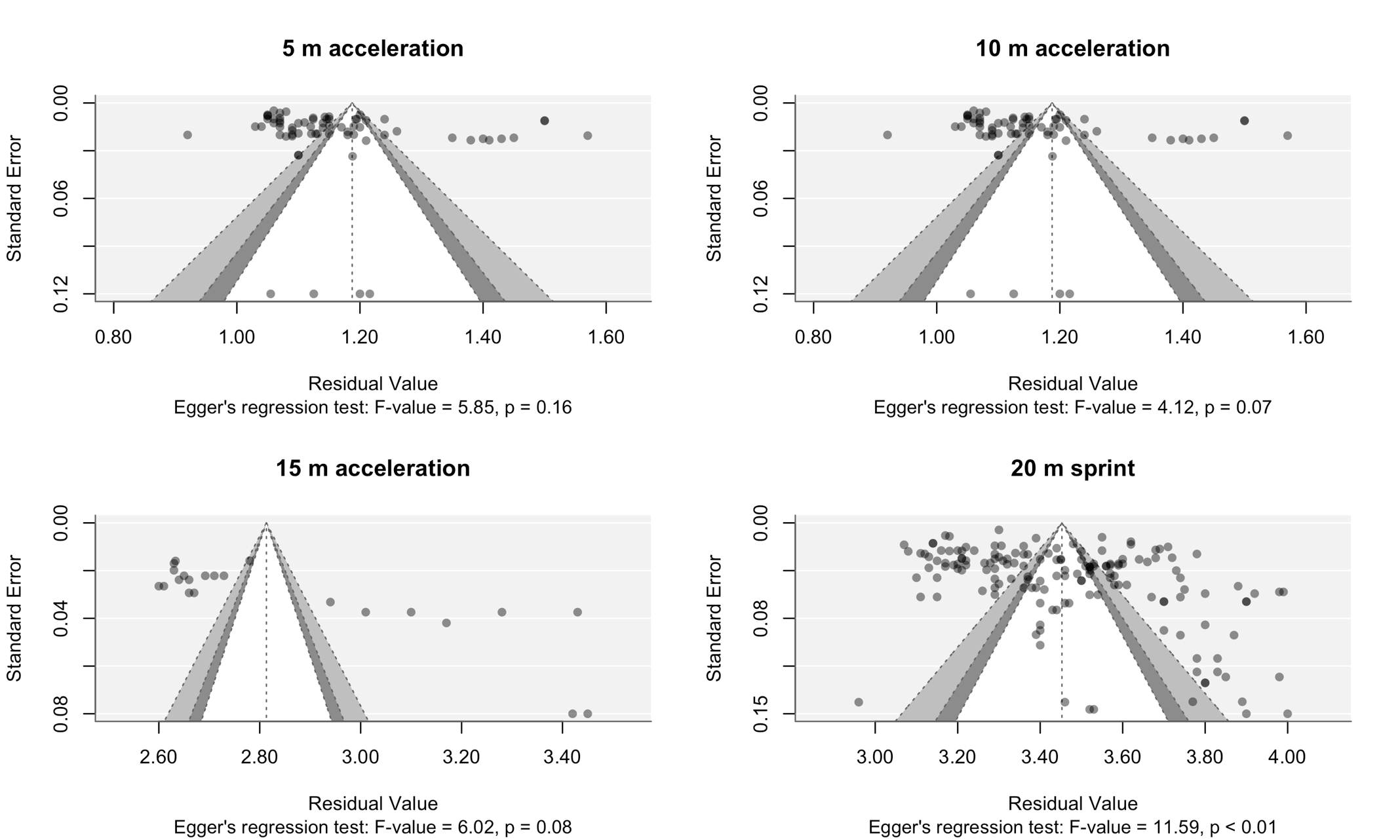


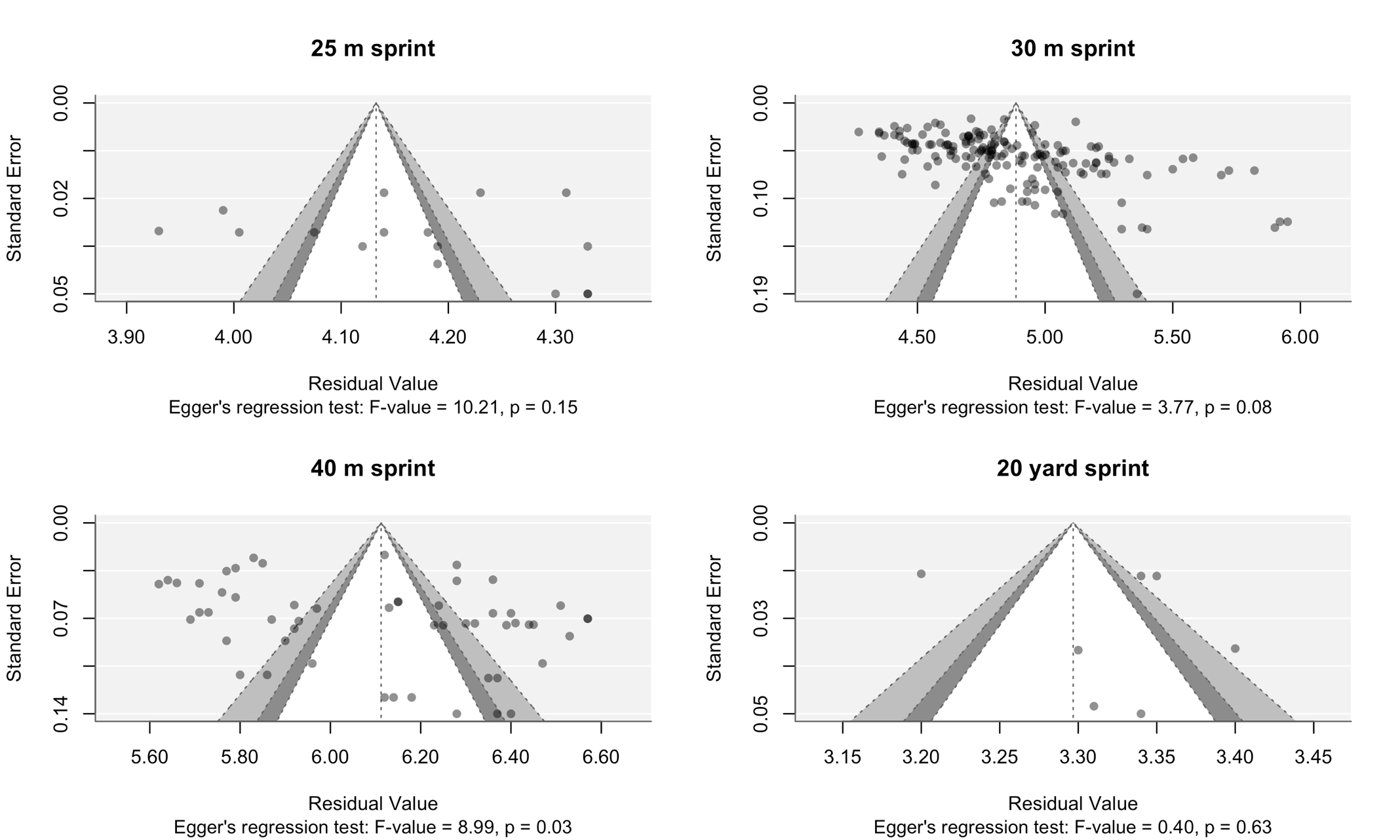


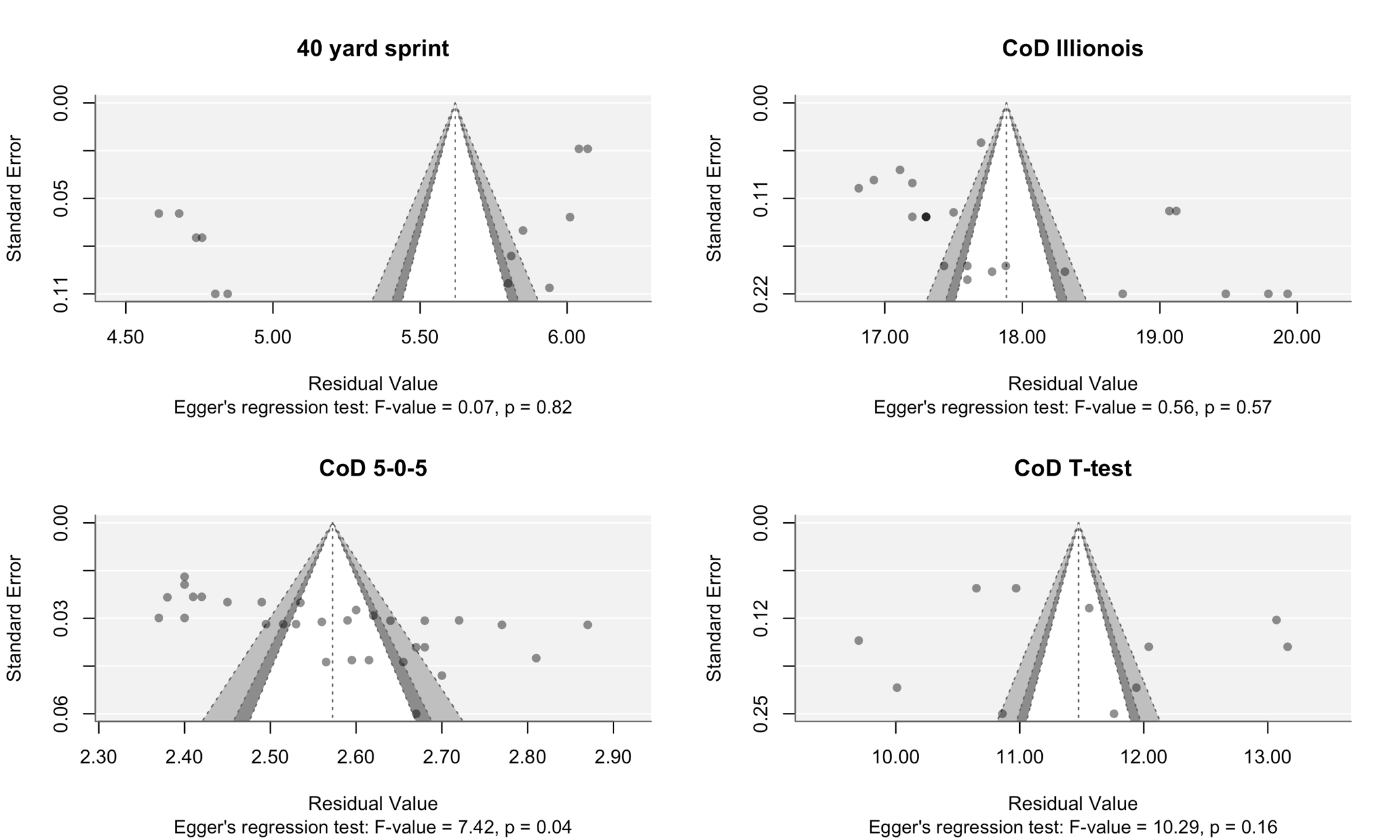


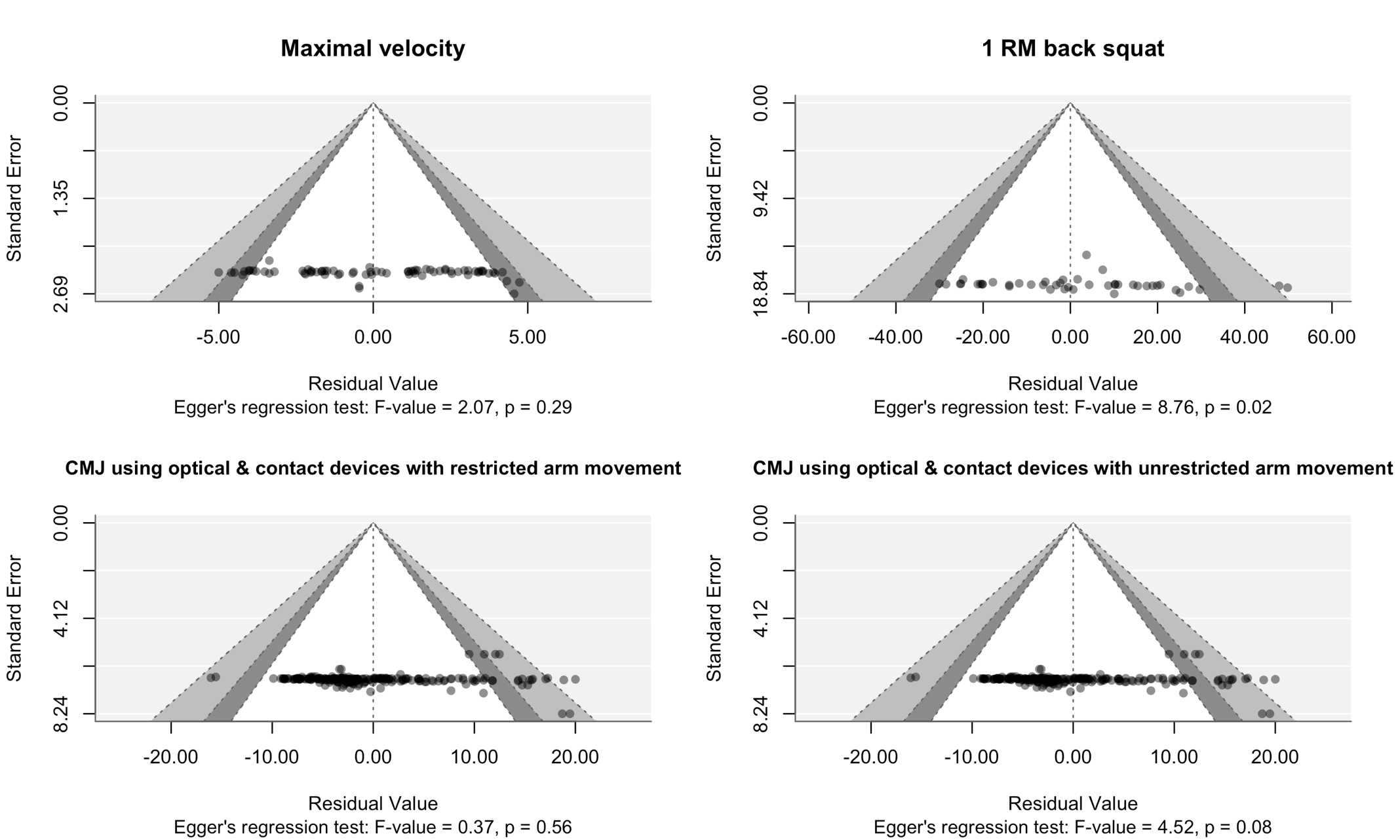


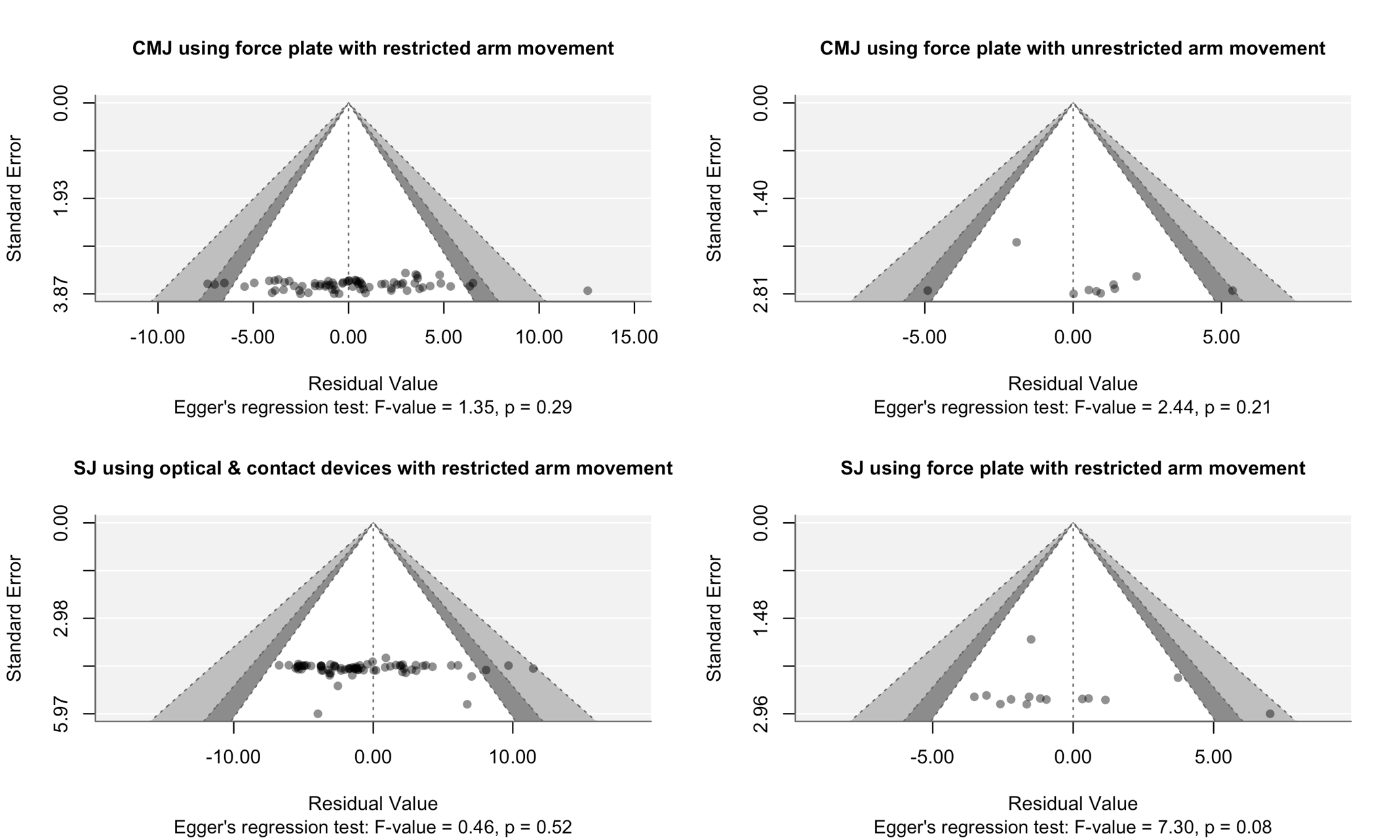


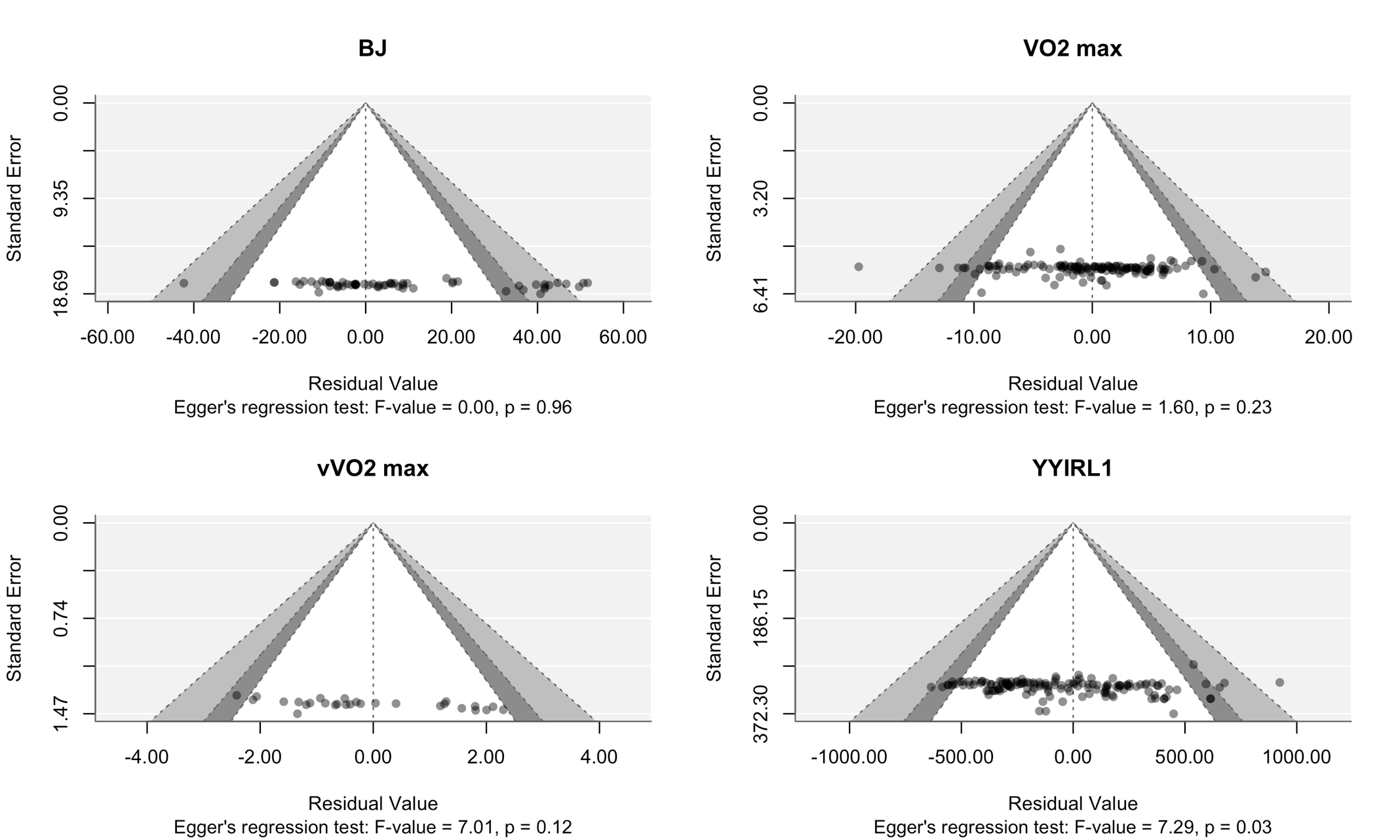


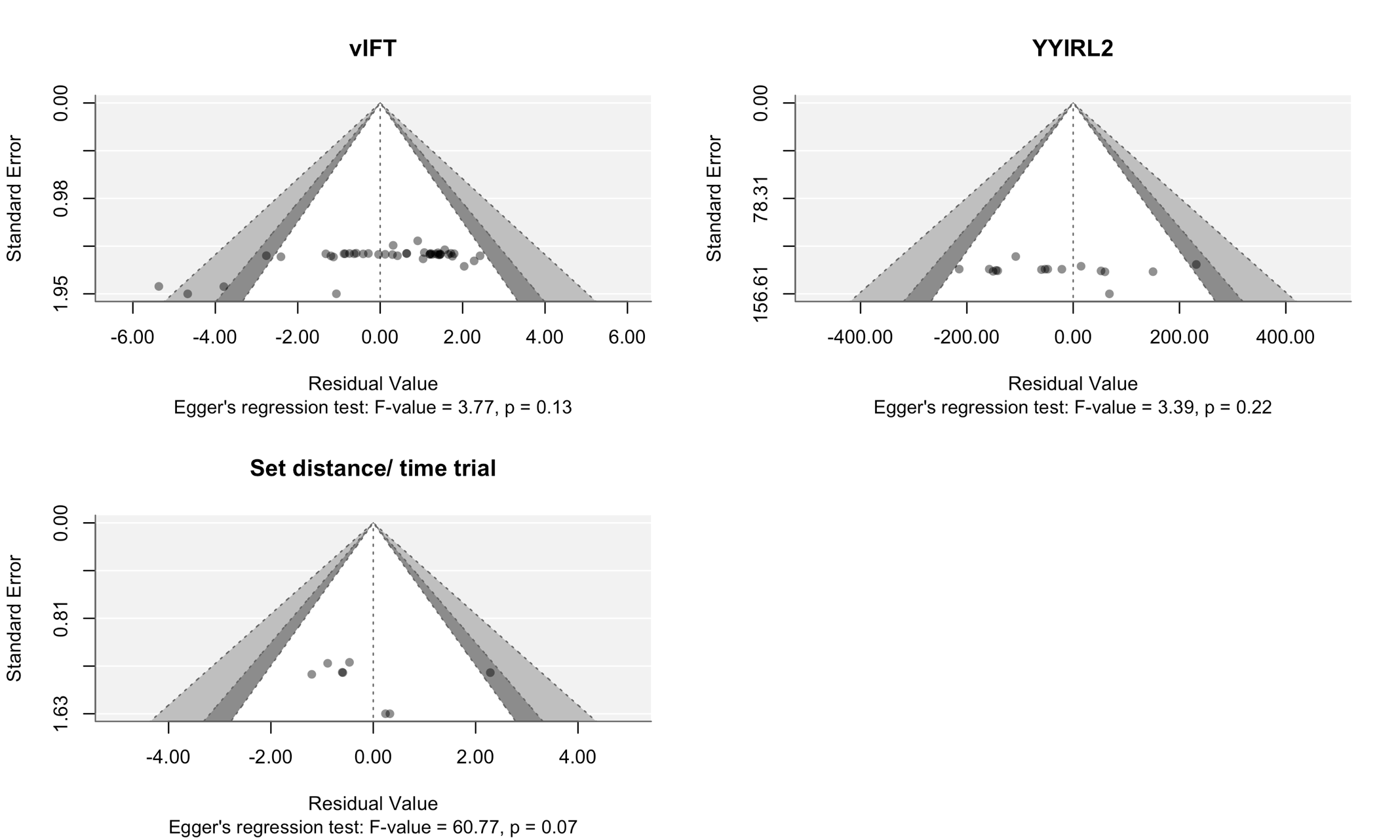


$\dot{V}$O_2_ max: maximal oxygen uptake, YYIRL1: Yo-Yo Intermittent Recovery Test Level 1, YYIRL2: Yo-Yo Intermittent Recovery Test Level 2, V_IFT_: final velocity attained during 30-15 Intermittent Fitness Test, _V_$\dot{V}$O_2_ max: velocity attained during graded maximal exercise tests, 1 RM: one repetition maximum, SJ: squat jump, CMJ: countermovement jump, BJ: broad jump, COD: change of direction
