# Supplementary material for: Benchmarking the Physical Performance Qualities in Women’s Football: A Systematic Review and Meta-analysis Across the Performance Scale
Source: Sports Med. 2025 Sep 1;56(Suppl 1):127–55. doi: 10.1007/s40279-025-02251-0 (PMC13314896; doi:10.1007/s40279-025-02251-0)
Supplement: Supplementary file 8 — Supplementary file8 (DOCX 31 KB) [file 40279_2025_2251_MOESM8_ESM.docx]

**Title:** Benchmarking The Physical Performance Qualities in Women’s Football: A Systematic Review and Meta-Analysis Across the Performance Scale

**Authors:**

Heidi R. Compton^1,2,3^ - 0000-0002-5818-4450

Ric Lovell^3,4^ - 0000-0001-5859-0267

Dawn Scott^3^ - 0009-0000-6763-1235

Jo Clubb^3,5^ - 0000-0002-6509-7531

Tzlil Shushan^3,4^ - 0000-0002-0544-1986

**Affiliations:**

^1^ School of Biomedical Sciences and Pharmacy, University of Newcastle, Australia;

^2^ Applied Sport Science and Exercise Testing Laboratory, University of Newcastle, Ourimbah, Australia;

^3^ FIFA, Women’s Development Programme, Zurich, Switzerland;

^4^ Faculty of Science, Medicine and Health, University of Wollongong, Australia;

^5^ Global Performance Insights Ltd, London, United Kingdom

**Corresponding author:**

Heidi Compton

Heidi.compton@newcastle.edu.au

University of Newcastle

Callaghan, Australia

| **Table S6.** Sensitivity analysis. | | | | |  |
| --- | --- | --- | --- | --- | --- |
| **Protocol and procedure** | **r values** | **Estimate** | **Lower 90% CI** | **Upper 90% CI** |  |
|  |  |  |  |  |  |
| 5 m acceleration | 0.3 | 1.19 | 1.14 | 1.23 |  |
| 5 m acceleration | 0.5 | 1.19 | 1.14 | 1.23 |  |
| 5 m acceleration | 0.7 | 1.19 | 1.14 | 1.23 |  |
| 5 m acceleration | 0.9 | 1.19 | 1.14 | 1.23 |  |
| 10 m acceleration | 0.3 | 1.99 | 1.96 | 2.01 |  |
| 10 m acceleration | 0.5 | 1.99 | 1.96 | 2.01 |  |
| 10 m acceleration | 0.7 | 1.99 | 1.96 | 2.01 |  |
| 10 m acceleration | 0.9 | 1.99 | 1.96 | 2.01 |  |
| 15 m acceleration | 0.3 | 2.81 | 2.64 | 2.99 |  |
| 15 m acceleration | 0.5 | 2.81 | 2.64 | 2.99 |  |
| 15 m acceleration | 0.7 | 2.81 | 2.64 | 2.99 |  |
| 15 m acceleration | 0.9 | 2.81 | 2.64 | 2.99 |  |
| 20 m sprint | 0.3 | 3.45 | 3.41 | 3.5 |  |
| 20 m sprint | 0.5 | 3.45 | 3.41 | 3.5 |  |
| 20 m sprint | 0.7 | 3.45 | 3.41 | 3.5 |  |
| 20 m sprint | 0.9 | 3.45 | 3.41 | 3.5 |  |
| 25 m sprint | 0.3 | 4.13 | 3.94 | 4.33 |  |
| 25 m sprint | 0.5 | 4.13 | 3.94 | 4.33 |  |
| 25 m sprint | 0.7 | 4.13 | 3.94 | 4.33 |  |
| 25 m sprint | 0.9 | 4.13 | 3.94 | 4.33 |  |
| 30 m sprint | 0.3 | 4.89 | 4.83 | 4.95 |  |
| 30 m sprint | 0.5 | 4.89 | 4.83 | 4.95 |  |
| 30 m sprint | 0.7 | 4.89 | 4.83 | 4.95 |  |
| 30 m sprint | 0.9 | 4.89 | 4.83 | 4.95 |  |
| 40 m sprint | 0.3 | 6.11 | 6.01 | 6.22 |  |
| 40 m sprint | 0.5 | 6.11 | 6.01 | 6.21 |  |
| 40 m sprint | 0.7 | 6.11 | 6.01 | 6.21 |  |
| 40 m sprint | 0.9 | 6.11 | 6.01 | 6.21 |  |
| 20 yard sprint | 0.3 | 3.3 | 3.16 | 3.43 |  |
| 20 yard sprint | 0.5 | 3.3 | 3.16 | 3.43 |  |
| 20 yard sprint | 0.7 | 3.3 | 3.16 | 3.43 |  |
| 20 yard sprint | 0.9 | 3.3 | 3.16 | 3.43 |  |
| 40 yard sprint | 0.3 | 5.62 | 4.9 | 6.34 |  |
| 40 yard sprint | 0.5 | 5.62 | 4.9 | 6.34 |  |
| 40 yard sprint | 0.7 | 5.62 | 4.9 | 6.34 |  |
| 40 yard sprint | 0.9 | 5.62 | 4.9 | 6.34 |  |
| illinois CoD | 0.3 | 17.88 | 17.33 | 18.43 |  |
| illinois CoD | 0.5 | 17.88 | 17.34 | 18.43 |  |
| illinois CoD | 0.7 | 17.88 | 17.34 | 18.43 |  |
| illinois CoD | 0.9 | 17.88 | 17.34 | 18.43 |  |
| 5-0-5 CoD | 0.3 | 2.57 | 2.52 | 2.63 |  |
| 5-0-5 CoD | 0.5 | 2.57 | 2.52 | 2.63 |  |
| 5-0-5 CoD | 0.7 | 2.57 | 2.52 | 2.63 |  |
| 5-0-5 CoD | 0.9 | 2.57 | 2.52 | 2.63 |  |
| T-test CoD | 0.3 | 11.47 | 10.55 | 12.4 |  |
| T-test CoD | 0.5 | 11.47 | 10.55 | 12.4 |  |
| T-test CoD | 0.7 | 11.47 | 10.55 | 12.4 |  |
| T-test CoD | 0.9 | 11.47 | 10.55 | 12.4 |  |
| Max velocity | 0.3 | 26.51 | 25.78 | 27.24 |  |
| Max velocity | 0.5 | 26.51 | 25.78 | 27.24 |  |
| Max velocity | 0.7 | 26.51 | 25.78 | 27.24 |  |
| Max velocity | 0.9 | 26.51 | 25.78 | 27.24 |  |
| 1 RM squat | 0.3 | 87.36 | 79.97 | 94.75 |  |
| 1 RM squat | 0.5 | 87.36 | 79.97 | 94.76 |  |
| 1 RM squat | 0.7 | 87.36 | 79.96 | 94.77 |  |
| 1 RM squat | 0.9 | 87.37 | 79.95 | 94.78 |  |
| CMJ optical restricted | 0.3 | 31.15 | 29.91 | 32.39 |  |
| CMJ optical restricted | 0.5 | 31.15 | 29.91 | 32.38 |  |
| CMJ optical restricted | 0.7 | 31.15 | 29.91 | 32.38 |  |
| CMJ optical restricted | 0.9 | 31.14 | 29.91 | 32.38 |  |
| CMJ optical unrestricted | 0.3 | 37.61 | 33.66 | 41.57 |  |
| CMJ optical unrestricted | 0.5 | 37.61 | 33.65 | 41.57 |  |
| CMJ optical unrestricted | 0.7 | 37.61 | 33.65 | 41.56 |  |
| CMJ optical unrestricted | 0.9 | 37.60 | 33.65 | 41.56 |  |
| CMJ force plate restricted | 0.3 | 28.27 | 27.24 | 29.29 |  |
| CMJ force plate restricted | 0.5 | 28.26 | 27.23 | 29.29 |  |
| CMJ force plate restricted | 0.7 | 28.26 | 27.23 | 29.29 |  |
| CMJ force plate restricted | 0.9 | 28.26 | 27.23 | 29.29 |  |
| CMJ force plate unrestricted | 0.3 | 32.96 | 30.45 | 35.47 |  |
| CMJ force plate unrestricted | 0.5 | 32.97 | 30.47 | 35.47 |  |
| CMJ force plate unrestricted | 0.7 | 32.98 | 30.48 | 35.48 |  |
| CMJ force plate unrestricted | 0.9 | 32.99 | 30.5 | 35.49 |  |
| SJ optical restricted | 0.3 | 28.88 | 27.49 | 30.27 |  |
| SJ optical restricted | 0.5 | 28.88 | 27.49 | 30.28 |  |
| SJ optical restricted | 0.7 | 28.88 | 27.49 | 30.28 |  |
| SJ optical restricted | 0.9 | 28.88 | 27.49 | 30.28 |  |
| SJ force plate restricted | 0.3 | 27.60 | 25.37 | 29.83 |  |
| SJ force plate restricted | 0.5 | 27.60 | 25.38 | 29.82 |  |
| SJ force plate restricted | 0.7 | 27.60 | 25.38 | 29.82 |  |
| SJ force plate restricted | 0.9 | 27.60 | 25.38 | 29.82 |  |
| BJ | 0.3 | 189.34 | 182.02 | 196.67 |  |
| BJ | 0.5 | 189.33 | 182 | 196.66 |  |
| BJ | 0.7 | 189.32 | 181.98 | 196.66 |  |
| BJ | 0.9 | 189.31 | 181.97 | 196.65 |  |
| $\dot{V}$O_2_ max | 0.3 | 49.00 | 47.84 | 50.15 |  |
| $\dot{V}$O_2_ max | 0.5 | 49.00 | 47.84 | 50.15 |  |
| $\dot{V}$O_2_ max | 0.7 | 49.00 | 47.84 | 50.15 |  |
| $\dot{V}$O_2_ max | 0.9 | 49.00 | 47.84 | 50.15 |  |
| _V_$\dot{V}$O_2_ max | 0.3 | 14.64 | 13.84 | 15.45 |  |
| _V_$\dot{V}$O_2_ max | 0.5 | 14.64 | 13.84 | 15.45 |  |
| _V_$\dot{V}$O_2_ max | 0.7 | 14.64 | 13.84 | 15.45 |  |
| vVO2 max | 0.9 | 14.64 | 13.84 | 15.45 |  |
| YYIRL1 | 0.3 | 1139.29 | 1068.8 | 1209.79 |  |
| YYIRL1 | 0.5 | 1139.00 | 1068.49 | 1209.51 |  |
| YYIRL1 | 0.7 | 1138.68 | 1068.16 | 1209.2 |  |
| YYIRL1 | 0.9 | 1138.36 | 1067.82 | 1208.89 |  |
| V_IFT_ | 0.3 | 17.98 | 16.98 | 18.98 |  |
| V_IFT_ | 0.5 | 17.98 | 16.99 | 18.98 |  |
| V_IFT_ | 0.7 | 17.99 | 17.00 | 18.98 |  |
| V_IFT_ | 0.9 | 17.99 | 17.02 | 18.97 |  |
| YYIRL2 | 0.3 | 445.46 | 363.21 | 527.71 |  |
| YYIRL2 | 0.5 | 445.59 | 363.34 | 527.84 |  |
| YYIRL2 | 0.7 | 445.65 | 363.41 | 527.89 |  |
| YYIRL2 | 0.9 | 445.69 | 363.46 | 527.92 |  |
| Set Time/Distance Trial | 0.3 | 12.68 | 11.32 | 14.05 |  |
| Set Time/Distance Trial | 0.5 | 12.69 | 11.31 | 14.06 |  |
| Set Time/Distance Trial | 0.7 | 12.69 | 11.31 | 14.07 |  |
| Set Time/Distance Trial | 0.9 | 12.69 | 11.30 | 14.08 |  |

$\dot{V}$O_2_ max: maximal oxygen uptake, YYIRL1: Yo-Yo Intermittent Recovery Test Level 1, YYIRL2: Yo-Yo Intermittent Recovery Test Level 2, V_IFT_: final velocity attained during 30-15 Intermittent Fitness Test, _V_$\dot{V}$O_2_ max: velocity attained during graded maximal exercise tests, 1 RM: one repetition maximum, SJ: squat jump, CMJ: countermovement jump, BJ: broad jump, CI: confidence intervals
