# Supplementary material for: Benchmarking the Physical Performance Qualities in Women’s Football: A Systematic Review and Meta-analysis Across the Performance Scale
Source: Sports Med. 2025 Sep 1;56(Suppl 1):127–55. doi: 10.1007/s40279-025-02251-0 (PMC13314896; doi:10.1007/s40279-025-02251-0)
Supplement: Supplementary file 9 — Supplementary file9 (DOCX 28 KB) [file 40279_2025_2251_MOESM9_ESM.docx]

**Title:** Benchmarking The Physical Performance Qualities in Women’s Football: A Systematic Review and Meta-Analysis Across the Performance Scale

**Authors:**

Heidi R. Compton^1,2,3^ - 0000-0002-5818-4450

Ric Lovell^3,4^ - 0000-0001-5859-0267

Dawn Scott^3,4^ - 0009-0000-6763-1235

Jo Clubb^3,5^ - 0000-0002-6509-7531

Tzlil Shushan^3,4^ - 0000-0002-0544-1986

**Affiliations:**

^1^ School of Biomedical Sciences and Pharmacy, University of Newcastle, Australia;

^2^ Applied Sport Science and Exercise Testing Laboratory, University of Newcastle, Ourimbah, Australia;

^3^ FIFA, Women’s Development Programme, Women’s Football Division, Zurich, Switzerland;

^4^ Faculty of Science, Medicine and Health, University of Wollongong, Australia;

^5^ Global Performance Insights Ltd, London, United Kingdom

**Corresponding author:**

Heidi Compton

Heidi.compton@newcastle.edu.au

University of Newcastle

Callaghan, Australia

| **Table S7.** A summary of meta-regression results. | | | | | | | | | | | |
| --- | --- | --- | --- | --- | --- | --- | --- | --- | --- | --- | --- |
| **Protocol and procedure** | | | | **Reference** | |  | **Difference** | |  | **Magnitude** | **Qualitative interpretation** |
|  |  |  |  | **Tier** | **Intercept (90% CIs)** |  | **Tier** | **Slope (90% CIs)** |  |  |  |
| **Cardiorespiratory fitness** | | | | | |  |  |  |  |  |  |
| $\dot{V}$O_2_ max | | |  | Tier 3 | 48.9 (47.3 to 50.6) |  | Tier 4 to 5 | 0.8 (–2.1 to 3.7) |  | Small | Inconclusive |
|  | | | | | | | | | | |  |
| YYIRL1 | | |  | Tier 2 | 921 (675 to 1167) |  | Tier 3 | 170 (–25 to 364) |  | Moderate | Trivial and substantial |
|  | | |  |  |  |  | Tier 4 to 5 | 354 (141 to 567) |  | Large | Substantial |
|  | | |  | Tier 3 | 1091 (991 to 1190) |  | Tier 4 to 5 | 184 (66 to 302) |  | Moderate | Substantial |
|  | | | | | | | | | | |  |
| vIFT | | |  | Tier 2 | 16.5 (14.1 to 18.9) |  | Tier 3 | 2.0 (–0.2 to 4.2) |  | Large | Trivial and substantial |
|  | | |  |  |  |  | Tier 4 to 5 | 2.5 (0.4 to 4.5) |  | Large | Substantial |
|  | | |  | Tier 3 | 18.5 (17.7 to 19.3) |  | Tier 4 to 5 | 0.5 (–1.9 to 2.9) |  | Small | Inconclusive |
|  | | | | | | | | | | |  |
| **Acceleration** | | | | | |  |  |  |  |  |  |
| 5 m | | |  | Tier 2 | 1.18 (1.03 to 1.33) |  | Tier 3 | 0.02 (–0.11 to 0.14) |  | Small | Inconclusive |
|  | | |  |  |  |  | Tier 4 to 5 | –0.01 (–0.14 to 0.13) |  | Trivial | Inconclusive |
|  | | |  | Tier 3 | 1.20 (1.13 to 1.26) |  | Tier 4 to 5 | –0.03 (–0.04 to 0.00) |  | Small | Trivial and substantial |
|  | | | | | | | | | | |  |
| 10 m | | |  | Tier 2 | 2.02 (1.94 to 2.11) |  | Tier 3 | –0.03 (–0.12 to 0.05) |  | Small | Inconclusive |
|  | | |  |  |  |  | Tier 4 to 5 | –0.06 (–0.14 to 0.02) |  | Moderate | Inconclusive |
|  | | |  | Tier 3 | 1.99 (1.96 to 2.02) |  | Tier 4 to 5 | –0.03 (–0.05 to 0.00) |  | Small | Trivial and substantial |
|  | | | | | | | | | | |  |
| **Sprint** | | | | | |  |  |  |  |  |  |
| 20 m | | |  | Tier 2 | 3.61 (3.50 to 3.72) |  | Tier 3 | –0.17 (–0.28 to –0.06) |  | Moderate | Substantial |
|  | | |  |  |  |  | Tier 4 to 5 | –0.22 (–0.32 to –0.11) |  | Large | Substantial |
|  | | |  | Tier 3 | 3.43 (3.39 to 3.48) |  | Tier 4 to 5 | –0.04 (–0.08 to –0.01) |  | Small | Substantial |
|  | | | | | | | | | | |  |
| 30 m | | |  | Tier 2 | 5.23 (5.08 to 5.38) |  | Tier 3 | –0.32 (–0.46 to –0.18) |  | Large | Substantial |
|  | | |  |  |  |  | Tier 4 to 5 | –0.47 (–0.60 to –0.34) |  | Large | Substantial |
|  | | |  | Tier 3 | 4.91 (4.82 to 5.00) |  | Tier 4 to 5 | –0.15 (–0.24 to –0.05) |  | Moderate | Substantial |
| C | | | | | | | | | | |  |
| **Change of direction** | | | | | |  |  |  |  |  |  |
| 5-0-5 | | |  | Tier 2 | 2.65 (2.51 to 2.80) |  | Tier 3 | –0.06 (–0.20 to 0.07) |  | Moderate | Inconclusive |
|  | | | | | | | | | | |  |
| **Maximal velocity** | | | | | |  |  |  |  |  |  |
| Measured and observed | | |  | Tier 3 | 26.3 (25.4 to 27.1) |  | Tier 4 and 5 | 0.8 (–0.1 to 0.7) |  | Small | Trivial and substantial |
|  | | | | | | | | | | |  |
| **Lower limb strength** | | | | | |  |  |  |  |  |  |
| 1 RM back squat | | |  | Tier 3 | 84.3 (69.0 to 99.5) |  | Tier 4 and 5 | –2.0 (–17.5 to 13.6) |  | Trivial | Inconclusive |
|  | | | | | | | | | | |  |
| **Lower limb power** | | | | | |  |  |  |  |  |  |
| SJ | Optical/contact | Restricted | | Tier 2 | 25.5 (22.9 to 28.1) |  | Tier 3 | 2.6 (–1.1 to 6.3) |  | Moderate | Inconclusive |
|  |  |  | |  |  |  | Tier 4 and 5 | 6.2 (2.7 to 9.7) |  | Large | Substantial |
|  |  |  | | Tier 3 | 28.1 (26.1 to 30.1) |  | Tier 4 and 5 | 3.6 (0.7 to 6.4) |  | Moderate | Substantial |
| CMJ | Force plate | Restricted | | Tier 3 | 28.5 (27.0 to 30.1) |  | Tier 4 and 5 | 0.2 (–3.1 to 3.4) |  | Trivial | Inconclusive |
|  | Optical/contact | Restricted | | Tier 2 | 30.0 (24.2 to 35.8) |  | Tier 3 | 0.01 (–4.9 to 4.9) |  | Trivial | Inconclusive |
|  |  |  | |  |  |  | Tier 4 and 5 | 3.3 (–3.6 to 10.2) |  | Moderate | Inconclusive |
|  |  |  | | Tier 3 | 30.0 (28.4 to 31.6) |  | Tier 4 and | 3.3 (1.3 to 5.4) |  | Moderate | Substantial |
|  |  | Unrestricted | | Tier 3 | 34.5 (31.3 to 37.8) |  | Tier 4 and | 8.8 (2.5 to 15.1) |  | Large | Substantial |
| BJ |  | Unrestricted | | Tier 2 | 189.7 (181.2 to 198.3) |  | Tier 3 | –0.7 (–6.2 to 4.9) |  | Trivial | Inconclusive |
|  |  |  | |  |  |  | Tier 4 and 5 | –0.1 (–5.7 to 5.5) |  | Trivial | Inconclusive |
|  |  |  | | Tier 3 | 189.1 (181.3 to 196.9) |  | Tier 4 and 5 | 0.5 (–6.2 to 7.2) |  | Trivial | Inconclusive |
|  | | | | | | | | | | |  |

$\dot{V}$O_2_ max: maximal oxygen uptake, YYIRL1: Yo-Yo Intermittent Recovery Test Level 1, _V_IFT = final velocity attained during 30-15 Intermittent Fitness Test, 1 RM: one repetition maximum, SJ: squat jump, CMJ: countermovement jump, BJ: broad jump, CI: confidence intervals.
