# Supplementary material for: Benchmarking the Physical Performance Qualities in Women’s Football: A Systematic Review and Meta-analysis Across the Performance Scale
Source: Sports Med. 2025 Sep 1;56(Suppl 1):127–55. doi: 10.1007/s40279-025-02251-0 (PMC13314896; doi:10.1007/s40279-025-02251-0)

Title: Benchmarking The Physical Performance Qualities in Women's Football: A Systematic Review and Meta-Analysis Across the Performance Scale

Authors:

Heidi R. Compton[1,2,3] - 0000-0002-5818-4450

Ric Lovell[3,4] - 0000-0001-5859-0267

Dawn Scott[3] - 0009-0000-6763-1235

Jo Clubb[3,5] - 0000-0002-6509-7531

Tzlil Shushan[3,4] - 0000-0002-0544-1986

Affiliations:

[1] School of Biomedical Sciences and Pharmacy, University of Newcastle, Australia;

[2] Applied Sport Science and Exercise Testing Laboratory, University of Newcastle, Ourimbah, Australia;

[3] FIFA, Women's Development Programme, Women's Football Division, Zurich, Switzerland;

[4] Faculty of Science, Medicine and Health, University of Wollongong, Australia;

[5] Global Performance Insights Ltd, London, United Kingdom

Corresponding author:

Heidi Compton

Heidi.compton@newcastle.edu.au

University of Newcastle

Callaghan, Australia

Figure S2. Distribution of risk of bias for each criteria.

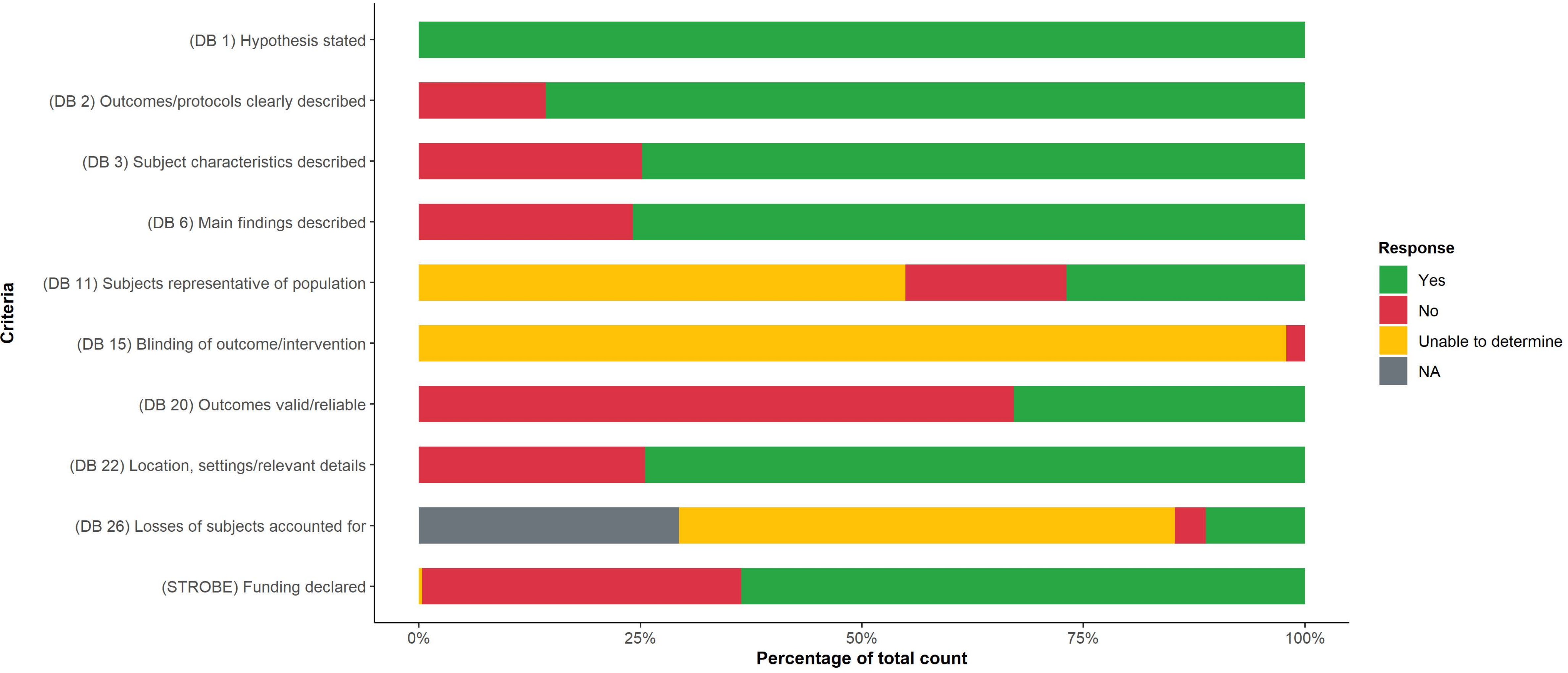

Supplement: Supplementary file 10 — Supplementary file10 (PDF 400 KB) [file 40279_2025_2251_MOESM10_ESM.pdf]
